# Graphene Oxide-Anchored Ru-dmso Catalyst for Sustainable Nitrile Hydrolysis in an Aqueous Medium

**DOI:** 10.1021/acs.inorgchem.6c02958

**Published:** 2026-09-03

**Authors:** Izan Roca, Nabila Mimouni, Syrine Affès, Xavier Fontrodona, Josep M. Luis, Isabel Romero

**Affiliations:** † Departament de Química and Serveis Tècnics de Recerca, 16738 Universitat de Girona, C/M. Aurèlia Capmany, 69, E-17003 Girona, Spain; ‡ Institut de Química Computacional I Catàlisi (IQCC) and Departament de Química, Universitat de Girona, E-17003 Girona, Catalonia, Spain

## Abstract

A ruthenium-dmso catalyst was heterogenized on graphene oxide (GO) for the first time, providing a sustainable system for nitrile hydrolysis in water. Three isomers of the [RuCl_2_(bpea-pyrene)­(dmso)] complex, *trans*,*mer*-**2a** (**2a**), *trans*,*fac*-**2b** (**2b**), and *cis*,*fac-* **2c** (**2c**) (bpea-pyrene = 1-[*bis*(pyridine-2-ylmethyl)­amino]­methylpyrene; dmso = dimethyl sulfoxide), were synthesized and characterized. Isomerization studies supported by DFT calculations confirmed that **2c** is the thermodynamically most stable isomer and elucidated the pathways connecting **2a**, **2b**, and **2c**. Photo- and thermochemically induced processes were investigated in various solvents, revealing distinct ligand substitution and oxidation pathways. Isomer **2c** was heterogenized on GO via π–π interactions between the pyrene unit and the support, representing the first example of a molecular Ru-dmso complex noncovalently anchored on GO. Both homogeneous **2c** and heterogeneous GO@**2c** efficiently catalyzed the transformation of nitriles to amides with high selectivity under aqueous conditions. The supported catalyst showed enhanced activity toward acrylonitrile, excellent reusability over five consecutive runs, negligible Ru leaching, and high structural stability. These findings establish GO@**2c** as an efficient, reusable, and sustainable catalyst for aqueous nitrile hydrolysis, and a plausible catalytic mechanism is proposed.

## Graphical abstract

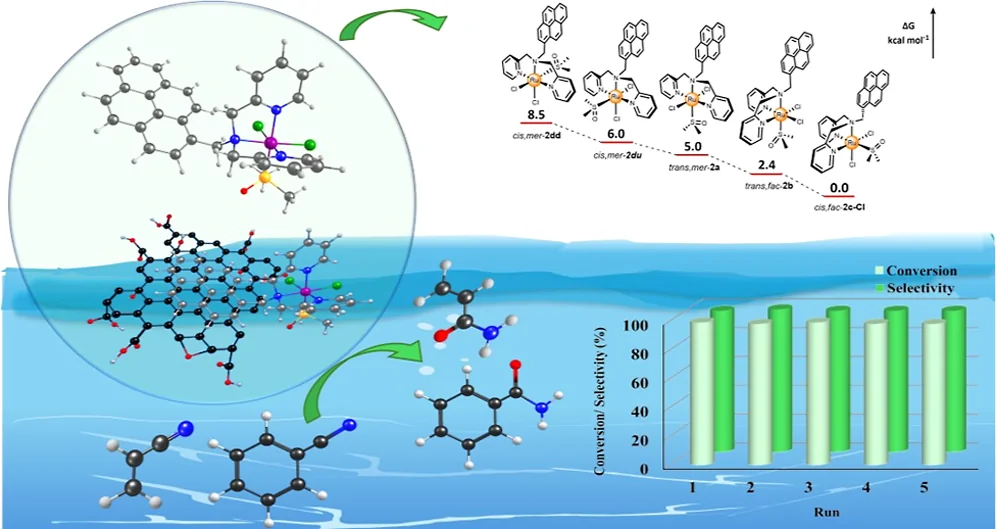

## Introduction

The development of sustainable protocols for synthetic transformations represents a major challenge in the chemical and pharmaceutical industries, where the use of catalysts is indispensable. Accordingly, the design and implementation of efficient, robust, environmentally benign, and recyclable catalytic systems are crucial to advancing the principles of green chemistry and addressing global sustainability challenges.

Amides constitute one of the most fundamental classes of organic compounds, as they are ubiquitous in biological systems, function as versatile intermediates in synthetic chemistry, and serve as essential raw materials in the polymer and pharmaceutical industries.^1–3^ The hydrolysis of nitriles to afford the corresponding amides is recognized as a desirable and atom-efficient method for amide synthesis, as it proceeds without the formation of toxic chemicals or byproducts. This transformation is also of biotechnological interest, given that nitrile hydratase enzymes are employed industrially for the large-scale production of important amides.^4–7^

Traditional approaches typically rely on strong acids or bases as catalysts; however, such harsh conditions are both environmentally and practically unsustainable.^8,9^ They often lead to overhydrolysis to carboxylic acids, reduced selectivity, and poor compatibility with sensitive functional groups.^10–12^ In contrast, transition metal-based catalysis has emerged as a more selective and milder alternative, particularly in homogeneous systems. Numerous efficient protocols employing metal complexes in solution have been developed, providing high selectivity under moderate reaction conditions.^13–19^

The principal advantages of homogeneous catalysis lie in its high selectivity and the ease with which the catalyst’s properties can be fine-tuned through ligand modification. However, a major drawback is the difficulty associated with separating the catalyst from the final reaction products. While trace amounts of residual metal are often tolerable in many industrial processes, they pose a critical issue in the pharmaceutical sector, where stringent purity standards must be met. Consequently, the development of robust and efficient heterogeneous catalysts is both appealing and essential, as such systems can be easily recovered and reused while providing a large surface area for catalytic activity.^20^ Recent advances in heterogeneous catalysis have also demonstrated the potential of supported transition-metal catalysts for nitrile transformations, including hydrogenation processes.^21–23^ These studies have highlighted the importance of metal–support interactions, which can significantly influence catalyst stability, activity, and selectivity. Such findings further emphasize the relevance of developing well-designed heterogeneous systems for efficient nitrile conversion processes. The most common strategy for immobilizing homogeneous catalysts onto solid supports involves the formation of covalent bonds between the ligand and the support.^24–26^ Nonetheless, this approach presents several drawbacks, including multistep synthetic procedures, high energy cost, and potential loss of catalytic activity. Various heterogeneous catalysts for nitrile hydrolysis have been reported in the literature, most of which rely on covalent anchoring of the catalyst to the corresponding support.^27–32^

Graphene oxide (GO) has emerged as an attractive alternative to conventional catalyst supports such as silica, metal oxides, and polymeric materials. Unlike conventional immobilization strategies based on covalent attachment, GO enables the immobilization of aromatic metal complexes through noncovalent π–π interactions^33–37^ between the GO surface and aromatic ligands, thereby avoiding ligand functionalization or covalent grafting, which may alter the catalyst structure or require multistep synthetic procedures. In addition, GO combines a high specific surface area, excellent thermal and mechanical stability, environmental friendliness, scalability, low cost and minimal mass transfer resistance, and outstanding dispersibility in aqueous media. These characteristics make GO an attractive support for heterogeneous catalysis while preserving the molecular integrity of the catalyst. Despite the growing interest in graphene-based catalytic materials, examples of well-defined ruthenium complexes immobilized on GO for nitrile hydrolysis remain scarce.^38^ Therefore, beyond the development of a recyclable catalytic system, the present work aims to further explore the potential of graphene oxide as a support for Ru-catalyzed nitrile hydrolysis. Several mechanisms for nitrile hydrolysis involve activating the CN bond toward nucleophilic attack, thereby enhancing the rate of hydrolysis and avoiding harsh reaction conditions.^39–41^ Among the mechanisms described in the literature, prominent pathways include the nucleophilic attack of a water molecule on the carbon atom of a nitrile coordinated to a transition metal. This process is typically facilitated either by polarization through hydrogen-bonding interactions^42–44^ or via a heteroatom on a ligand attached to the metal center.^45,46^

We have previously reported the first examples of ruthenium–dmso complexes, specifically [Ru^II^Cl_2_(N_2_)­(dmso-S)_2_] and [Ru^II^Cl_2_(N)­(dmso-S)_3_], where N_2_ and N denote a bidentate N-donor ligand and a monodentate N-donor ligand, respectively, which were successfully applied to this type of reaction in environmentally friendly media (notably water and glycerol).^47,48^ These complexes exhibited high efficiency and selectivity for the corresponding amide derivatives under mild conditions. Furthermore, we immobilized these compounds onto silica and nanoparticles to investigate their catalytic behavior.^27^ In the present work, we extend this strategy to graphene oxide, exploiting its ability to immobilize pyrene-functionalized Ru complexes through π–π interactions without covalent modification of the ligand framework. To our knowledge, complexes such as [Ru^II^Cl_2_(N_3_)­(dmso-S)], where N_3_ denotes a tridentate N-donor ligand, have not been previously reported in the literature as catalysts for nitrile hydrolysis, nor have any analogous graphene-supported ruthenium catalysts. Building upon established substitution processes for Ru-dmso complexes^49^ and their implications for catalytic mechanisms, we herein report the synthesis and characterization of three isomers of the [RuCl_2_(bpea-pyrene)­(dmso)] complex: *trans*,*mer*-**2a** (**2a**), *trans*,*fac-* **2b** (**2b**), and *cis*,*fac* **-2c** (**2c**). All three contain the N-tridentate, 1-[*bis*(pyridine-2-ylmethyl)­amino]­methylpyrene (bpea-pyrene).^37^ DFT calculations confirmed that **2c** is the thermodynamically stable product. Detailed thermal studies were conducted to elucidate the proposed mechanism by which these compounds promote nitrile hydrolysis. Additionally, the photoinduced behavior of isomers **2a** and **2c** was investigated in various solvents (CH_2_Cl_2_, H_2_O, and CH_3_CN). Furthermore, **2c** was successfully immobilized onto a graphene oxide (GO) support in water via noncovalent interactions, and the resulting heterogeneous system was fully characterized.

Finally, a comprehensive analysis of the catalytic performance of both the homogeneous **2c** and its heterogeneous counterpart, GO@**2c**, in the hydrolysis of representative nitriles in an aqueous medium is presented. Additionally, the recyclability of the GO@**2c** catalyst was evaluated, and a plausible mechanism is proposed to account for the observed reactivity.

## Experimental Section

### Materials

All reagents used in the present work were obtained from Sigma-Aldrich and used without further purification. Reagent-grade organic solvents were obtained from Carlo Erba, and high-purity deionized water was obtained by passing distilled water through a Nanopure Milli-Q water purification system.

### Instrumentation and Measurements

IR spectra were recorded on an Agilent Cary 630 FTIR spectrometer equipped with an ATR MK-II Golden Gate Single Reflection system. UV–vis spectroscopy was performed on a Cary 50 Scan (Varian) UV–vis spectrophotometer with 1 cm quartz cells. CV and DPV experiments were performed in an IJ-Cambria 660C potentiostat using a three-electrode cell. A GC electrode (3 mm diameter) from BAS was used as the working electrode, a platinum wire as the auxiliary electrode, and an SCE (saturated calomel electrode) as the reference electrode. All cyclic voltammograms presented in this work were recorded under a nitrogen atmosphere. The complexes were dissolved in solvents containing the necessary amount of *n*-Bu_4_NPF_6_ (TBAPF_6_) as the supporting electrolyte to yield a 0.1 M ionic strength solution. All *E*_1/2_ values reported in this work were estimated from cyclic voltammetry experiments as the average of the oxidative and reductive peak potentials (*E*_pa_ + *E*_pc_)/2, or directly from DPV. Unless explicitly mentioned, the concentration of the complexes was approximately 1 mM. NMR spectroscopy was performed on Bruker DPX 400 MHz spectrometers. Samples were recorded in CDCl_3_ or CD_2_Cl_2_. Elemental analyses were performed using a CHNS–O Elemental Analyzer EA-1108 from Fisons. ESI-MS experiments were performed on a Navigator LC/MS chromatograph from Thermo Quest Finnigan, using acetonitrile as the mobile phase. TEM studies were carried out using a JEOL JEM 1210 at 120 kV. Scanning electron SEM and EDX analysis were performed using the QUANTA FEI 200 FEG-ESEM and FESEM Hitachi S4100 devices. For metal content determination, a sequential inductively coupled plasma atomic emission spectrometer (ICP-AES), Agilent 7500c, Agilent Technologies, Tokyo, Japan, was used. Prior to measurements, samples were digested with HCl/H_2_O/HNO_3_ at room temperature. XPS measurements were performed at room temperature with a SPECS PHOIBOS 150 hemispherical analyzer (SPECS GmbH, Berlin, Germany) with a base pressure of 5 × 10^–10^ mbar using a monochromatic Al Kα radiation (1486.74 eV) operated at 300 W as an excitation source. The energy resolution measured by the fwhm of the Ag 3d_5/2_ peak for a sputtered silver foil was 0.62 eV.

### Gas Chromatography Studies

Gas chromatography was performed with an Agilent 7820A chromatograph equipped with an HP-5 column, 25 m × 0.20 mm (i.d); FID detector: 250 °C; carrier gas: He, rate: 1.51 mL min^–1^. The quantification was achieved from calibration curves. All the products and their corresponding substrates of catalysis were detected under the following conditions. Acrylonitrile: column temperature, 30 °C for 7 min, raising to 170 °C at a rate of 10 °C/min, holding 170 °C for 2 min. Benzonitrile, chlorobenzonitrile, methylbenzonitrile, and chloroacetonirile: column temperature, 40 °C for 5 min, raising to 170 °C at a rate of 10 °C/min, holding 170 °C for 2 min. In all the analyses, biphenyl was used as the internal standard.

### Crystallographic Data Collection and Structure Determination

The X-ray intensity data were measured on a Bruker D8 QUEST ECO three-circle diffractometer system equipped with a ceramic X-ray tube (Mo Kα, λ = 0.71076 Å) and a doubly curved silicon crystal Bruker Triumph monochromator, using the APEX3 software package.^50^ The frames were integrated with the Bruker SAINT.^51^ Data was corrected due to absorption effects using the Multi-Scan method (SADABS).^52^ The structures were solved and refined using the Bruker SHELXTL.^53^

### Computational Studies

The density functional theory (DFT) calculations were performed using the Gaussian 16 package.^54^ Three functionals were used: B3LYP,^55^ ωB97XD,^56^ and M06-2X,^57^ in combination with the def2-SVP and def2-TZP basis sets for H, C, N, O, and S; the same basis sets with the corresponding quasi-relativistic effective core pseudopotentials (ECPs) for Ru; and def2-SVPD and def2-TZVPD for Cl.^58^ For B3LYP calculations, Grimme’s D3 dispersion correction^59^ with Becke–Johnson damping GD3BJ^60^ was used, while for M06-2X calculations, GD3 correction was used. Diffuse orbitals from the def2-SVPD and def2-TZVPD basis sets were used to describe chlorine atoms due to their role as anionic ligands. In the geometry optimizations, performed at the B3LYP/def2-SVP­(H, C, N, O, S, Ru)/def2-SVPD­(Cl) level of theory, the initial geometries correspond to the crystallographic structures of the complexes. Geometry optimizations were followed by analytical frequency calculations to confirm the nature of the stationary points (minimum and transition states). IRC calculations were performed to confirm the connection between the transition states and the corresponding reactants and products. In the single-point calculations, solvent effects were included using the solvation model based on density (SMD), an implicit continuum solvent model, with ethanol as the solvent.^61^ Thermochemical corrections to enthalpy and entropy were applied using the quasi-harmonic approximation described by Grimme,^62^ introduced by the software GoodVibes.^63^ The quasi-harmonic correction was applied for the normal modes with frequencies lower than 100 cm^–1^, which typically correspond to rotational modes that can lead to significant error if the harmonic model is used. GoodVibes was used to further evaluate the thermochemical properties of the complexes at 80 °C (353 K) and a molarity of 0.024 M, corresponding to the experimental conditions of the complex synthesis. The total final Gibbs energy values (*G*) are given by

1 $$G=E_{sp}+G_{corr}$$

where *E*_sp_ represents the electronic energy of the single-point calculations on the equilibrium geometries, and *G*_corr_ is the Gibbs energy correction obtained from the thermodynamic analysis using the GoodVibes code.

A data set collection of computational results is available in the ioChem-BD repository and can be accessed at DOI: 10.19061/iochem-bd-6-657.

### Preparations

The ligand bpea-pyrene was prepared according to the methods described in the literature.^37^ All spectroscopic, electrochemical, and synthetic experiments were performed in the absence of light unless explicitly mentioned.

### Synthesis of Complexes

#### Synthesis of *trans*,*mer*-[Ru^II^Cl_2_(bpea-Pyrene)­(dmso)], *trans*,*mer*-2a (**2a**)

A 0.117 g (0.240 mmol) sample of [RuCl_2_(dmso)_4_] and 0.100 g (0.240 mmol) of the bpea-pyrene ligand dissolved in 10 mL of dry ethanol were refluxed for 30 min in the dark under a N_2_ atmosphere. After cooling to room temperature, a yellow solid, corresponding to the **2a** isomer, was precipitated. The solid was filtered and washed with cold ethanol and diethyl ether. Yield: 0.111 gr (69%). Suitable crystals of *trans,fac-*[Ru^II^Cl_2_(bpea-pyrene)­(dmso)], *trans,fac-* **2b** (**2b**), were grown as pale-orange plates after evaporation of the mother liquor of isomer **2a**. Yield: 0.035 gr (22%).

##### *trans*,*mer*-**2a**

^**1**^ **H NMR** (400 MHz, CD_2_Cl_2_): δ 8.96 (d, 2H, H1,H13 *J*_1,2_ = 6.5 Hz), 8.43–8.10 (m, 7H, H18–H31), 7.97 (d, 1H, H28, *J*_28,27_ = 9.3 Hz), 7.90 (d, 1H, H17, *J*_17,18_ = 7.7 Hz), 7.86 (t, 2H, H3, H11, *J*_3,2_ = *J*_3,4_ = *J*_11,12_ = *J*_11,10_ = 7.7 Hz), 7.48 (d, 2H, H4, H10, *J*_4,3_ = *J*_10,11_ = 7.7 Hz), 7.41 (dd, 2H, H2, H12, *J*_2,3_ = *J*_12,11_ = 7.7 Hz, *J*_2,1_ = *J*_12,13_ = 6.5 Hz), 5.56 (d, 2H, H6a,H8a, *J*_6b,6a_ = *J*_8b,8a_ = 15.0 Hz), 4.95 (s, 2H, H7), 4.59 (d, 2H, H6b, H8b, *J*_6a,6b_ = *J*_8b,8a_ = 15.0 Hz), 3.61 (s, 6H, H14,H15). ^**13**^ **C NMR** (400 MHz, CD_2_Cl_2_): δ 157 (C1, C13), 136 (C3, C11), 129 (C17), 128–126 (C18–C31), 124 (C2, C12), 124 (C28), 122 (C4, C10), 61 (C6, C8), 57 (C7), 48 (C14, C15). *E*_1/2_ **Ru** ^**III**^ **/Ru** ^**II**^(CH_2_Cl_2_ + 0.1 M TBAPF_6_) = 0.58 V vs SCE. **UV–vis** (CH_3_OH) [λ_max_, nm (ε, M^–1^ cm^–1^)]: 265 (17,304), 275 (24,725), 330 (13,660), 345 (16,505), 415 (2923). **IR** (*v*_max_, cm^–1^): 3026, 2921, 1606, 1457, 1434, 1303, 1241, 1047, 1006, 838, 760, 678.

##### *trans*,*fac*-**2b**

^**1**^ **H NMR** (400 MHz, CD_2_Cl_2_): δ 9.46 (d, 2H, H1, H13, *J*_1,2_ = *J*_13,12_ = 6.5 Hz), 8.72 (d, 1H, H17, *J*_17,18_ = 9.5 Hz), 8.43–8.19 (m, 8H, H18–H31), 7.46 (t, 2H, H3, H11, *J*_3,2_ = *J*_3,4_ = *J*_11,12_ = *J*_11,10_ = 7.7 Hz), 7.12 (dd, 1H, H2, H12, *J*_2,3_ = *J*_12,11__=_ 7.7 Hz, *J*_2,1_ = *J*_12,13_ = 6.5 Hz), 6.96 (d, 2H, H4, H10, *J*_4,3_ = *J*_10,11_ = 7.7 Hz), 6.11 (s, 2H, H7), 5.41 (d, 2H, H6b, H8b, *J*_6b,6a_ = *J*_8b,8a_ = 15.6 Hz), 3.76 (d, 2H, H6a, H8a, *J*_6a,6b_ = *J*_8a,8b_ = 15.6 Hz), 3.57 (s, 6H, H14, H15). ^**13**^ **C NMR** (400 MHz, CD_2_Cl_2_): δ 156 (C1, C13), 135 (C3, C11), 131–125 (C18–C31), 126 (C28), 124 (C17), 124 (C2, C12), 121 (C4, C10), 65 (C6, C8), 61 (C7), 47 (C14, C15). *E*_1/2_ **Ru** ^**III**^ **/Ru** ^**II**^ (CH_2_Cl_2_ + 0.1 M TBAPF_6_) = 0.60 V vs SCE. **UV–vis** (CH_3_OH) [λ_max_, nm (ε, M^–1^ cm^–1^)]: 265 (35,415), 275 (47,846), 325 (24,000), 344 (30,160), 410 (3451). **IR** (*v*_max_, cm^–1^): 3034, 2918, 1604, 1475, 1436, 1303, 1244, 1051, 1011, 849, 763, 679.

#### Synthesis of *cis*,*fac-*[Ru^II^Cl_2_(bpea-Pyrene)­(dmso)], *cis*,*fac-* **2c** (**2c**)

##### Method **1**

A 0.099 g (0.21 mmol) sample of [RuCl_2_(dmso)_4_] and 0.085 g (0.21 mmol) of the bpea-pyrene ligand dissolved in 10 mL of dry ethanol were refluxed for 12 h in the dark under a N_2_ atmosphere. After cooling to room temperature, a yellow solid corresponding to the **2c** isomer was precipitated. The solid was filtered and washed with cold ethanol and ethyl ether. Yield: 0.138 g (85%).

##### Method **2**

A solution of **2a** or **2b** in dry ethanol was refluxed for 12 h in the dark under a N_2_ atmosphere. After cooling to room temperature, the volume was reduced on the rotatory evaporator, and the solid corresponding to **2c** was filtered and washed with cold ethanol and diethyl ether.

^**1**^ **H NMR** (400 MHz, CD_2_Cl_2_): δ 9.79 (dd, 1H, H1, *J*_1,2_ = 6.8 Hz, *J*_1,3_ = 1.6 Hz), 9.31 (dd, 1H, H13, *J*_13,12_ = 6.8 Hz, *J*_13,11_ = 1.6 Hz), 8.86 (d, 1H, H17, *J*_17,18_ = 9.5 Hz), 8.37–8.16 (m, 7H, H18–H31), 8.11 (d, 1H, H28, *J*_28,27_ = 7.6 Hz), 7.58 (td, 1H, H3, *J*_3,4_ = *J*_3,2_ = 7.7 Hz, *J*_3,1_ = 1.6 Hz), 7.41 (td, 1H, H11, *J*_11,10_ = *J*_11,12_ = 7.7, *J*_11,13_ = 1.6 Hz), 7.29 (dd, 1H, H2, *J*_2,3_ = 7.7 Hz, *J*_2,1_ = 6.8 Hz), 7.06 (dd, 1H, H12, *J*_12,11_ = 7.7 Hz, *J*_12,13_ = 6.8 Hz), 6.99 (d, 1H, H4, *J*_4,3_ = 7.7 Hz), 6.88 (d, 1H, H10, *J*_10,11_ = 7.7 Hz), 5.98 (d, 1H, H7a, *J*_7a,7b_ = 14.3 Hz), 5.93 (d, 1H, H7b, *J*_7b,7a_ = 14.3 Hz), 5.20 (d, 1H, H8b, *J*_8b,8a_ = 15.3 Hz), 4.92 (d, 1H, H6b, *J*_6b,6a_ = 15.7 Hz), 3.72 (s, 3H, H15), 3.67 (d, 1H, H8a, *J*_8a,8b_ = 15.3 Hz), 3.37 (d, 1H, H6a, *J*_6a,6b_ = 15.7 Hz), 3.17 (s, 3H, H14). ^**13**^ **C NMR** (400 MHz, CD_2_Cl_2_): δ 154 (C13), 152 (C12), 136 (C3), 135 (C11), 128–126, (C18–C31), 126 (C28), 123 (C17, C2, C4),120 (C3), 120 (C11), 69 (C8), 67 (C6), 63 (C7), 44 (C14), 44 (C15). *E*_1/2_ **Ru** ^**III**^ **/Ru** ^**II**^(CH_2_Cl_2_ + 0.1 M TBAPF_6_) = 0.61 V vs SCE. **UV–vis** (CH_3_OH) [λ_max_, nm (ε, M^–1^ cm^–1^)]: 265 (29,816), 275 (43,672), 325 (30,586), 345 (39,997), 385 (4728). **IR** (*v*_max_, cm^–1^): 3027, 2961, 2915, 1602, 1474, 1432, 1302, 1067, 1010, 839.1, 773, 681.

**Anal. Found** (Calcd) for RuC_31_H_29_N_3_SOCl_2_·2.5H_2_O·CH_2_Cl_2_: C, 48.11 (48.43); H, 4.21 (4.40); N, 5.60 (6.33) %.

#### Synthesis of *cis*,*fac*-[Ru^II^Cl­(bpea-Pyrene)­(CH_3_CN)­(dmso)]­Cl, *cis*,*fac-* **5s**

A solution of 50 mg (0.10 mmol) of the isomer *cis*,*fac*-**2c** and 1 mL of CH_3_CN (19 mmol) was refluxed in 20 mL of absolute ethanol for 24 h in the dark. After cooling to room temperature, the volume of the mixture was reduced using a rotary evaporator. The concentrated solution was treated with diethyl ether, inducing the precipitation of an orange solid, which was collected by filtration and washed with cold diethyl ether. **Yield**: 0.034 g (68%) **IR** (*v*_max_, cm^–1^): 3026, 2921, 2280, 1435, 1315, 1047, 1006, 838, 760. **UV–vis** (MeCN) ([λ_max_, nm [ε, L·mol^–1^·cm^–1^]): 345 (17,300), 295 (17,990), 290 (25,170), 285 (29,930). *E*_1/2_ **Ru** ^**III**^ **/Ru** ^**II**^ (MeCN +0.1 M TBAPF_6_) = 0.86 V vs SCE. **ESI-MS (** *m*/*z* **)**: 669.10 [M–Cl]^+^, 628.08 [M–Cl–CH_3_CN]^+^, 591.09 [M–Cl–dmso]^+^, 278.06 [M–dmso–2Cl]^2+^, 257.55 [M–dmso–MeCN–2Cl]^2+^.

### Functionalization of GO with Complex *cis*,*fac-* **2c**

#### GO@*cis*,*fac-*[Ru^II^Cl_2_(bpea-Pyrene)­(dmso)], GO@*cis*,*fac*-**2c** (GO@**2c**)

50 mg of GO suspended in 15 mL of water/acetone (9:1) was sonicated for 30 min. Then, 10 mg of the **2c** complex was added. The suspension was stirred for 24 h in the dark at room temperature. The black solid was filtered and washed with water. The corresponding solid was analyzed by ICP–MS to calculate the amount of the anchored complex. *E*_1/2_ Ru^III^/Ru^II^(CH_2_Cl_2_ + 0.1 M TBAPF_6_) = 0.59 V vs SCE.

#### Homogeneous Catalysis

The nitrile substrate (1 mmol) and ruthenium catalyst (0.01 mmol) were suspended in 2.5 mL of water and sealed in a reaction tube. The reaction mixture was stirred in the dark at 100 °C for 20 h. The resulting solution, containing the remaining nitrile, was extracted with a solution of biphenyl as an internal standard prepared in chloroform (4 × 2 mL). The aqueous phase, containing the amide, was dried on a watch glass for subsequent NMR analysis. The organic phase was analyzed by GC.

#### Heterogeneous Catalysis

Nitrile (0.16 mmol) and the heterogenized ruthenium catalyst (1.6 × 10^–3^ or 0.008 mmol, calculated considering the quantity of complex supported on GO) were suspended in 2.5 mL of water in a sealed reaction tube, and the reaction mixture was stirred at 100 °C for 6 or 20 h. After completion, the solution was filtered to isolate the catalyst, which was washed with water (4 × 2 mL). The resulting solution, containing the remaining nitrile, was extracted with a solution of biphenyl as an internal standard prepared in chloroform (4 × 2 mL). The aqueous phase was dried in a watch glass to analyze it by NMR, and the organic phase was analyzed by GC.

#### Recycling Experiments

The reaction conditions indicated above were used in these experiments. For every run and after 6 or 20 h, the catalyst was recovered from the reaction mixture by filtration, washed with UPW and diethyl ether, and dried. Afterward, the solid was exposed to a new load of substrate under the same experimental conditions.

#### Safety Statement

No uncommon hazards were noted.

## Results and Discussion

### Synthesis and Characterization

#### Synthesis of Molecular Complexes and Structural Characterization

The bpea-pyrene ligand was obtained following the literature procedures.^37^ The synthetic preparation of the complexes is shown in Scheme 1. The addition of equimolecular amounts of ligand bpea-pyrene to the [RuCl_2_(dmso)_4_] complex in dry ethanol at reflux for 30 min, under a nitrogen atmosphere in the dark, leads to the precipitation of the *trans*,*mer*-[Ru^II^Cl_2_(bpea-pyrene)­(dmso)] **2a** isomer, while the more soluble *trans*,*fac-*[Ru^II^Cl_2_(bpea-pyrene)­(dmso)] **2b** isomer is formed in the mother liquor in lower yield. Extending the reflux time to 12 h, using the same starting materials, yields the *cis*,*fac-*[Ru^II^Cl_2_(bpea-pyrene)­(dmso)] **2c** isomer. This species can also be prepared by heating **2a** and **2b** at reflux for 12 h. These observations demonstrate that **2a** and **2b** are kinetic products, whereas **2c** is a thermodynamically controlled product.

**Scheme 1:**
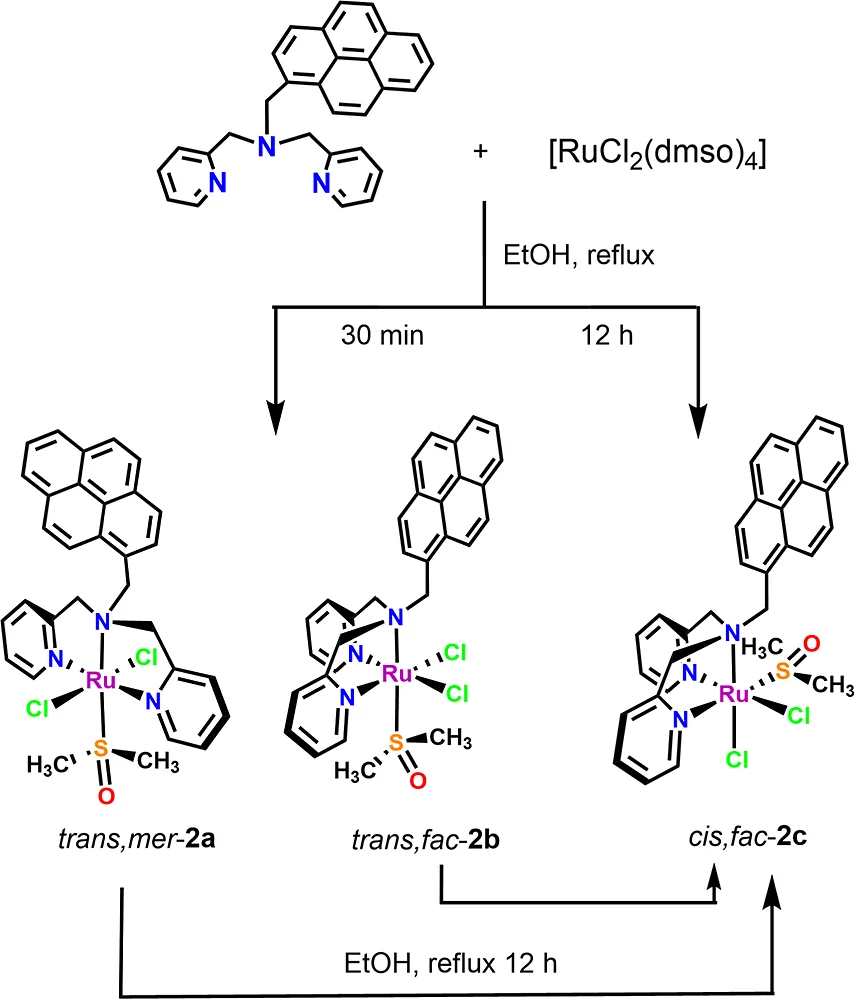
Synthetic Strategy for the Preparation of **2a**, **2b**, and **2c**

In these complexes, *fac*-/*mer*-nomenclature describes the coordination mode of the bpea-pyrene ligand, while *trans*- and *cis*- refer to the position of the dmso ligand relative to the aliphatic nitrogen (*N*_al_) of the tridentate scaffold. We have isolated three of the five possible isomers (Scheme S1), which were characterized by single-crystal X-ray diffraction. Figure 1 displays their molecular structures, while Tables S1 and S2 provide the main crystallographic data, along with selected bond distances and angles. The description of the structures is displayed in the Supporting Information (Figures S1 and S2).

**Figure 1:**
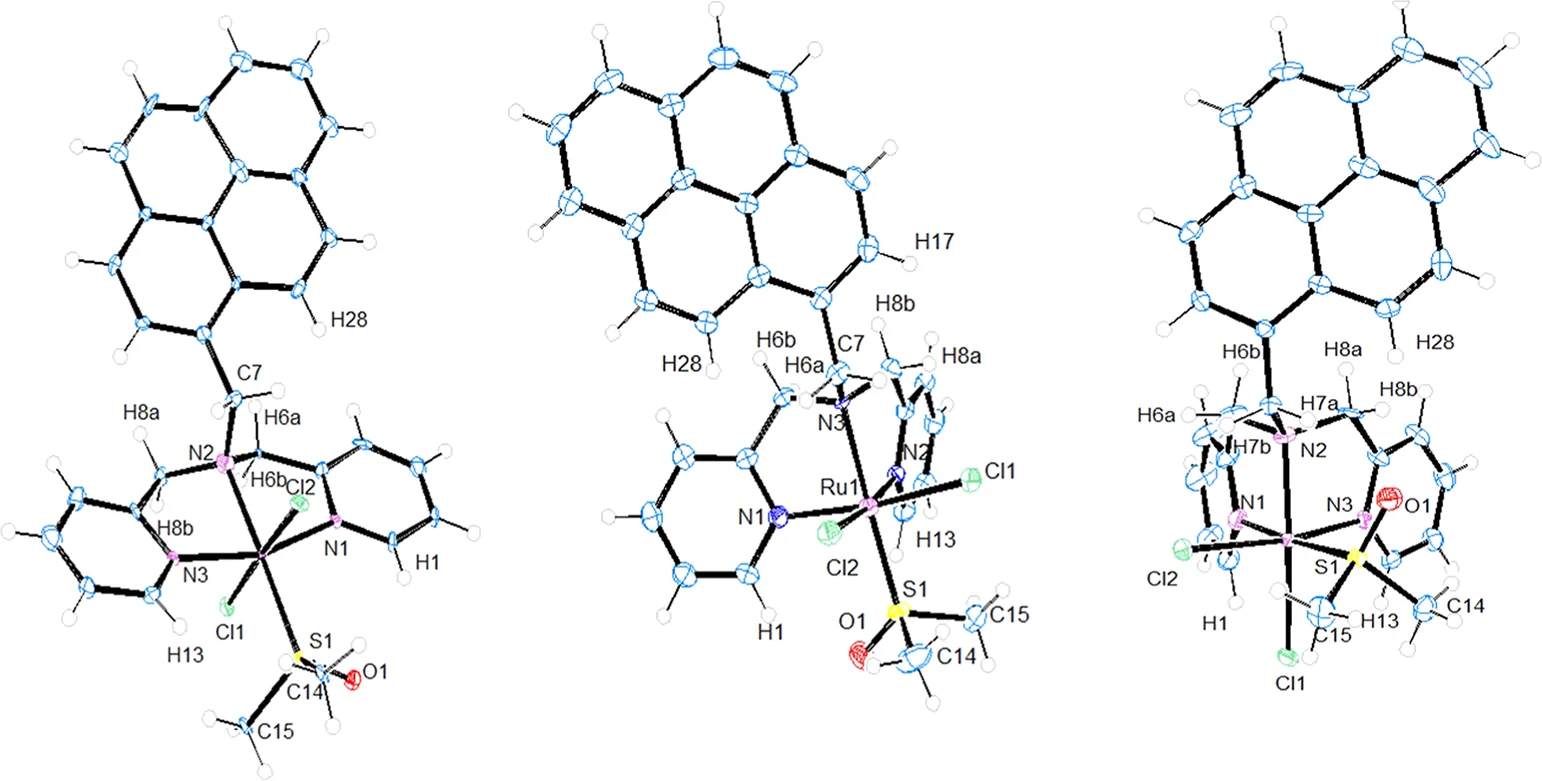
Molecular structure and labeling schemes for (a) **2a**, (b) **2b**, and (c)**2c**.

### Spectroscopic Characterization

The spectroscopic characterization of the three isomers was performed using IR and both one-dimensional (1D) and two-dimensional (2D) NMR spectroscopy (Figures S3–S6). The IR spectra of the three isomers exhibit nearly identical patterns. As shown in Figure S3, the bands in the 3100–2900 and 1600–1450 cm^–1^ regions are assigned to the ν_N–H_, ν_=C–H_, and ν_C=N_ stretching modes of the polypyridyl ligands. The bands observed around 1050–1100 cm^–1^ are attributed to the ν_s=o_ stretching vibration of the sulfur-coordinated dmso ligand. The ν­(S=O) stretching frequencies are very similar for the three isomers, in agreement with their closely related coordination environments, and are consistent with those reported for analogous sulfur-bound Ru–dmso complexes.^49^

The ^1^H NMR spectra of the isomers show two sets of resonances. The first, in the aromatic region, corresponds to the pyrene and pyridyl protons of the bpea-pyrene ligand. The second set, located in the aliphatic region, includes the methylene protons of the N–CH_2_–pyridyl and the N–CH_2_–pyrene groups, respectively, of the bpea-pyrene ligand, as well as the methyl groups of the coordinated dmso ligands. The observed resonances are consistent with the presence of the corresponding isomers and align with the solid-state structures. In both *trans*,*mer*- and *trans*,*fac-*isomers, the coordination of the dmso ligand *trans*- to the aliphatic nitrogen of the ligand renders the two pyridyl rings and the methyl groups of the dmso ligands magnetically equivalent, with the methyl groups appearing as a singlet in **2a** and **2b** due to the symmetry of both complexes, regardless of whether the tridentate ligand adopts a meridional or facial coordination. In both cases, the molecular geometry features a plane defined by the Ru center, the dmso ligand, and the aliphatic nitrogen atom. The pyridyl protons H1 and H13 appear significantly further downfield in **2b** (δ = 9.46 ppm, Figure S5a) than in **2a** (δ = 8.96 ppm, Figure S4a). This difference is consistent with the different spatial arrangement of the coordinated chlorido and dmso ligands, which modifies the local magnetic environment around these protons. The crystal structures reveal different Cl···H and O­(dmso)···H contacts in the two isomers, which are consistent with distinct local environments that likely contribute to the observed deshielding. In both isomers, the methylene protons (H6a, H6b and H8a, H8b) appear as two distinct doublets due to their nonequivalent environments, with H6a and H8a resonating further downfield in the *fac-*isomer. The methylene protons of the N–CH_2_–pyrene group (H7) appear as singlets, indicating a similar magnetic environment for both protons; these signals are slightly upfield-shifted in the *mer-*isomer. For **2c** (Figure 2), the asymmetry of the molecule leads to an increase in the number of resonances, due to the nonequivalence of the protons. This includes the diastereotopic methylene protons (H7a,b), which now appear as two distinct doublets. Similarly, the methylene protons (H6a, H6b and H8a, H8b) give rise to four separate doublets. The methyl groups of the dmso ligand also appear as two singlets, reflecting their chemical nonequivalence.

**Figure 2:**
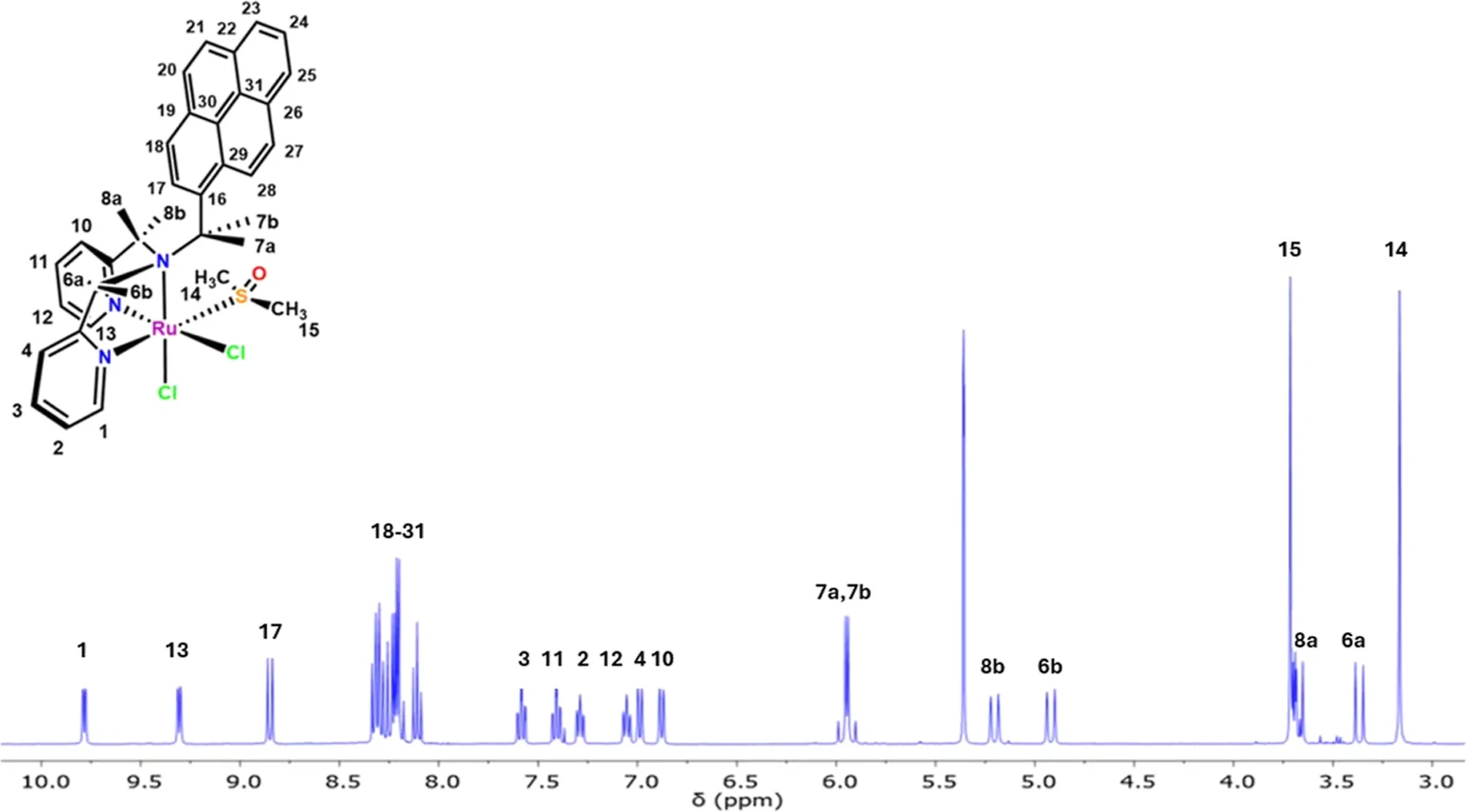
^1^H NMR spectrum of the **2c** isomer in CD_2_Cl_2_.

The 2D NOESY spectrum (Figure S6d) exhibits NOE correlations between the methyl protons of the DMSO ligand (δ = 3.17 ppm) and the H13 proton of the bpea-pyrene ligand (δ = 9.3 ppm). These interactions facilitated the assignment of the individual methyl groups of the DMSO ligand in this isomer.

The UV–vis spectra recorded in MeOH (Figure S7) display intense ligand-centered (LC) π–π transitions in the 250–350 nm region, which are mainly associated with the bpea-pyrene ligand, as confirmed by comparison with the spectrum of the free ligand (Figure S7, Supporting Information). At longer wavelengths, the spectra exhibit metal-to-ligand charge-transfer (MLCT) transitions^64^ involving Ru dπ orbitals and ligand π orbitals. For complexes **2a** and **2b**, the MLCT bands are observed at 415 and 410 nm, respectively, whereas for **2c**, the corresponding transition appears as a weak shoulder at ca. 385 nm, partially overlapped by the intense ligand-centered absorption. The absorptions in the visible region are assigned to MLCT transitions, in agreement with related Ru–dmso complexes reported in the literature.^49^

It is worth mentioning that **2a** exhibited a red shift in the MLCT transition compared to **2b** and **2c**, displaying a distinct pattern of MLCT absorption bands. This can be interpreted as the *trans*- arrangement of the chlorido ligands leading to a relative destabilization of the donor dπ­(Ru) orbitals, which is in full agreement with the increases in the HOMO energy level. These values also agree with the redox potentials observed for the different isomers (vide infra).

### Electrochemical Properties

Electrochemical measurements of the isomers were performed in CH_2_Cl_2_ + 0.1 M tetrabutylammonium hexafluorophosphate (TBAPF_6_), using glassy carbon electrodes as working electrodes. Figure S8a shows the cyclic voltammetry (CV) experiments. The three isomers show a quasi-reversible oxidation wave assigned to the Ru­(III)/Ru­(II) redox couple at *E*_1/2_ = 0.58 V vs SCE for **2a**, 0.60 V for **2b**, and 0.61 V for **2c**. All compounds display an irreversible oxidation peak around 1.40 V corresponding to the electro-oxidation of the pyrene monomer to its cationic radical.^65^

An anodic shift of 30 mV for **2c** and 20 mV for **2b** was observed relative to the **2a** isomer, which is consistent with the UV–vis spectra and the higher thermal stability of **2c**. Notably, unlike the other two isomers, **2a** undergoes rapid dmso linkage isomerization upon oxidation of the Ru^II^(dmso-S) species to Ru^III^(dmso-S), forming Ru^III^(dmso-O). This species is then reduced to Ru^II^(dmso-O) upon back-scanning, with a cathodic peak at *E*_pc_ = 0.15 V. The shift toward lower potentials for the O-coordinated dmso complex is due to the lower electron-withdrawing ability of the oxygen atom, which stabilizes the higher oxidation states of Ru. By varying the equilibration time during CV and starting the scan at *E*_in_ = 1.20 V (cathodic scan), further evidence of linkage isomerization was obtained. Figure S8b shows the CV of **2a** at equilibration times of 2 and 20 s; as this time increases, the isomerization process becomes more pronounced as more Ru^III^(dmso-O) is formed. The fact that linkage isomerization occurs exclusively for the least thermodynamically stable mer isomer (**2a**), while it is not observed for the isomers (**2b** and **2c**), suggests that the lower stability of **2a** facilitates structural rearrangement upon oxidation. Furthermore, the absence of linkage isomerization in the analogous complex lacking the pyrene substituent^49^ suggests that the pyrene moiety may also contribute to this behavior. Although the origin of this effect cannot be established from the present results, it may arise from subtle electronic and/or conformational changes induced by the pyrene substituent, which could slightly modify the activation barrier for dmso linkage isomerization. Further mechanistic studies are required to clarify its role.

### Theoretical Calculations

Theoretical analysis based on DFT calculations was carried out to assess the stability of the synthesized complex and its possible geometrical isomers. By considering the bpea-pyrene ligand arranged in both meridional and facial coordination modes, as well as different dispositions of the dmso ligand on the molecule, five isomers are differentiated: *cis*,*mer*-**2dd** (**2dd**), *cis*,*mer*-**2du** (**2du**), *trans*,*mer*-**2a** (**2a**), *trans*,*fac*-**2b** (**2b**), and *cis*,*fac*-**2c** (**2c**) (Scheme S1). Here, we report the results obtained at the ωB97X-D/def2-TZVP­(H,C,N,O,S,Ru)/def2-TZVPD­(Cl)/B3LYP/def2-SVP­(H,C,N,O,S,Ru)/def2-SVPD­(Cl) level of theory, including SMD solvation (ethanol) corrections and quasi-harmonic enthalpic and entropic corrections at 353.15 K to mimic the experimental conditions. Several other functional/basis set combinations were benchmarked (Table S3). For the **2dd**, **2du**, **2a**, and **2b** isomers, the orientation of the pyrene does not affect the overall energy because the chemical environment is identical to their mirror image. However, for the **2c** isomer, there are two energetically distinct conformers depending on whether the pyrene is closer to the chlorido or the sulfoxide ligand. This holds true for any intermediate lacking symmetry. Nevertheless, the transition states corresponding to the rotation of the pyrene moiety between these conformers are not considered, as this rotation is essentially barrierless at the reaction temperature. This barrierless rotation of the pyrene is demonstrated by the NMR spectra of **2a** and **2b** (Figures S4 and S5), as both isomers present equivalent proton pairs at room temperature. Consequently, two conformers were optimized: *cis*,*fac*-**2c**-Cl (**2c-Cl**), where the pyrene is closer to the chlorido ligand, and *cis*,*fac*-**2c**-S (**2c-S**), where it is located closer to the sulfoxide ligand. The relative stability of these conformers is highly solvent-dependent. In ethanol, the **2c**-Cl conformer is 0.3 kcal mol^–1^ more stable than **2c**-S, whereas in dichloromethane, this energy difference narrows to 0.06 kcal mol^–1^. This is further evidenced by the crystallographic structure of **2c** in dichloromethane, where the conformer **2c-S** is predominant in the crystal (Figure 1c). The solvent dependence can be rationalized by the strong capacity of the sulfoxide ligand to act as an H-bond acceptor species. The steric hindrance exerted by the pyrene prevents the formation of H-bonds between the sulfoxide ligand and the solvent, which destabilizes the molecule.

The calculated Gibbs free energy of the isomers is consistent with experimental observations (Figure 3). The **2a** isomer is formed after 30 min of reaction, whereas the more stable **2b** isomer is isolated from the mother liquor. During the synthesis, it was observed that extending the reflux time beyond 30 min leads to the formation of a mixture of **2a** and **2b** in solution, supporting the isomerization of **2a** into **2b**. The **2c** isomer is confirmed as the most thermodynamically favorable species, representing the final product of the equilibrium. Both *cis*,*mer*-isomers share the same nomenclature; however, **2dd** is 2.5 kcal mol^–1^ higher in energy. This difference is attributed to steric effects, as a bulkier sulfoxide ligand presents higher steric hindrance from the pyrene moiety of the bpea-pyrene ligand, resulting in molecule destabilization. The **2a** isomer structure presents both chlorido and aromatic nitrogen ligands *trans* to each other. This disposition is electronically less favorable than that of **2b**, in which σ-donor and π-acceptor ligands are *trans* to each other, stabilizing the molecule. The lower stability of **2b** relative to **2c** can be attributed to the axial position of the sulfoxide ligand, which is much more stabilized in the equatorial plane of the octahedral geometry due to its strong π-acceptor character.

**Figure 3:**
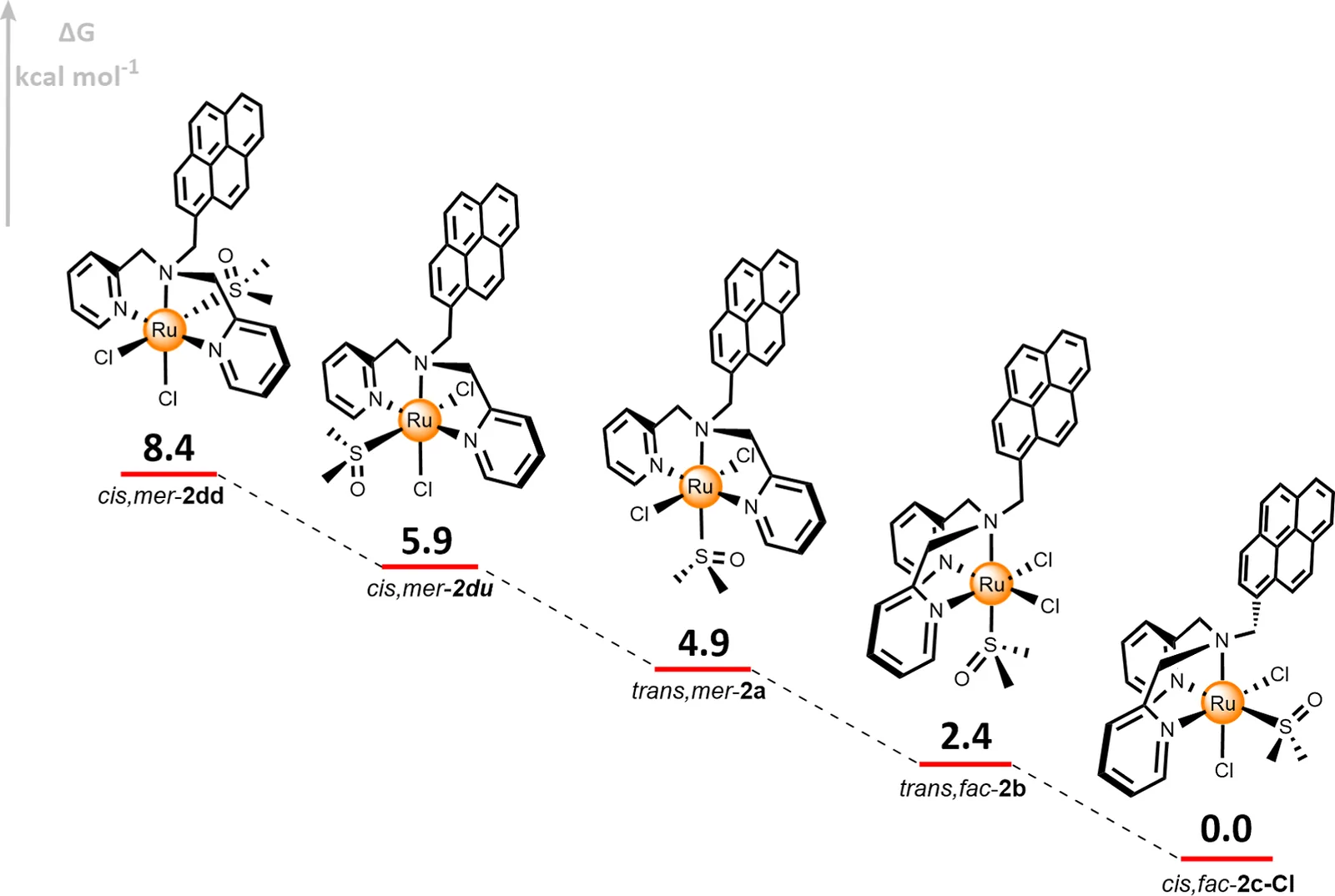
Gibbs free energy diagram of the isomers computed at the ωB97XD/def2-TZVP­(H,C,N,O,S,Ru)/def2-TZVPD­(Cl)//B3LYP/def2-SVP­(H,C,N,O,S,Ru)/def2-SVPD­(Cl) level, including solvent effects (ethanol) and quasi-harmonic enthalpic and entropic corrections at 353.15 K (in kcal mol^–1^).

Different possible isomerization pathways of **2a** to **2b**, and these into the **2c** isomer, were computed. The first pathway begins with the dissociation of the chlorido ligand farther from the pyrene moiety, generating the Int1-**2ab-cd** intermediate, which lies 9.3 kcal mol^–1^ higher in energy (Figure 4a). The successive rotation of the pyridine below the pyrene onto its fac conformation leads to the formation of TS1-**2ab-cd**. Along this rearrangement pathway, the bpea-pyrene ligand undergoes a marked change in its N5–Ru1–N6–N7 dihedral angle, shifting from 178° in **2a** to 143° in the transition state, which lies at 25.6 kcal mol^–1^. Further rotation decreases the dihedral angle to 91°, positioning the rotated aromatic ring *trans* to the remaining chlorido ligand, while the vacant site is trans to the other aromatic ring, generating the Int2-**2ba-cd** intermediate, which lies at 7.5 kcal mol^–1^. Finally, the coordination of the previously dissociated chlorido ligand at the vacant site yields **2b**, which possesses a dihedral angle of 94°, being 10 and 2.5 kcal mol^–1^ more stable than the Int2-**2ba-cd** intermediate and **2a**, respectively.

**Figure 4:**
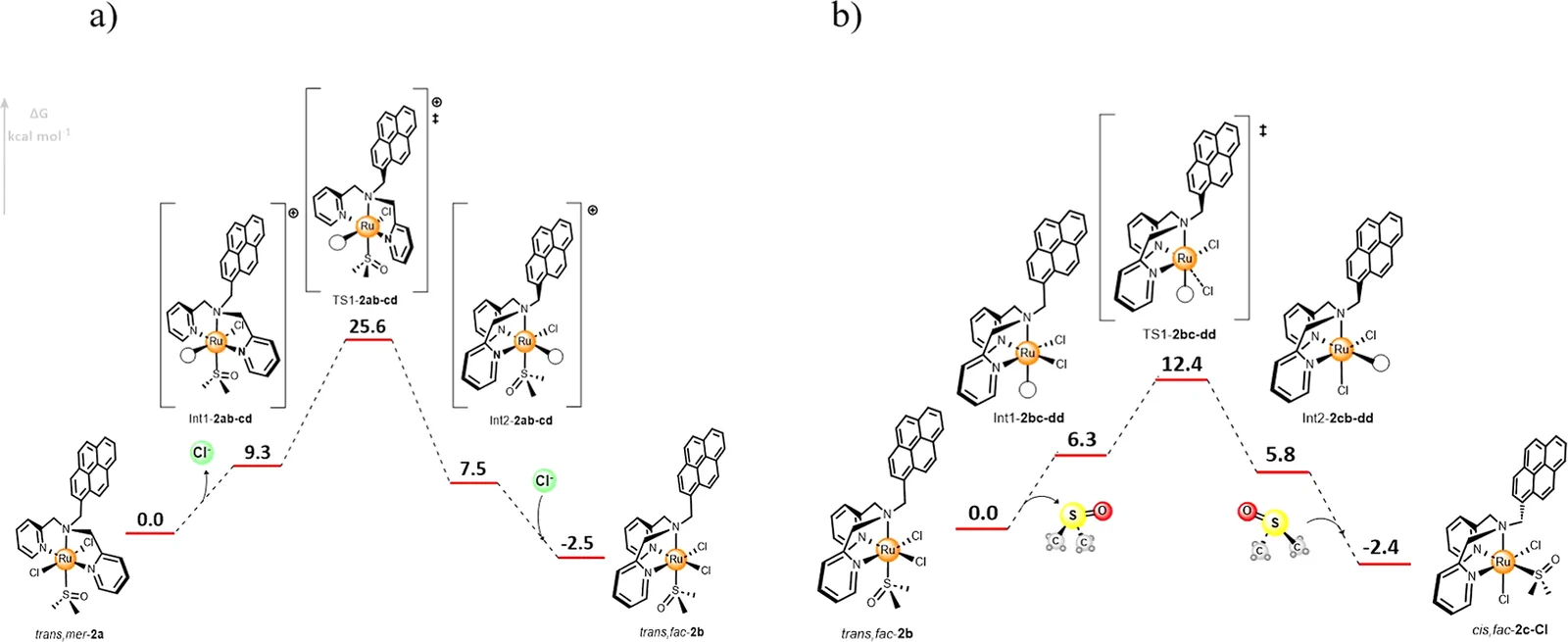
(a) Gibbs free energy profile of the proposed mechanism for the isomerization of *trans*,*mer*-**2a** into the *trans*,*fac*-**2b** isomer via chloride dissociation. (b) Gibbs free energy profile of the proposed mechanism for the isomerization of *trans*,*fac*-**2b** into the *cis*,*fac*-**2c** isomer via sulfoxide dissociation; both Gibbs free energy profiles are computed at the ωB97XD/def2-TZVP­(H,C,N,O,S,Ru)/def2-TZVPD­(Cl)//B3LYP/def2-SVP­(H,C,N,O,S,Ru)/def2-SVPD­(Cl) level including solvent effects (ethanol) and quasi-harmonic enthalpic and entropic corrections at 353.15 K (in kcal mol^–1^).

The second pathway begins with the dissociation of the sulfoxide ligand (Figure S9). This pathway leads to the formation of **2c**, without requiring the formation of **2b**. Although the DFT barrier for this pathway is slightly lower (23.7 kcal mol^–1^), the experimental evidence indicating the formation of the **2b** isomer supports the chlorido dissociation mechanism. To evaluate the isomerization of **2b** into **2c**, the dissociation of each ligand other than the bpea-pyrene ligand was computed.

The intermediate Int1-**2bc-dd** was generated upon sulfoxide dissociation, which lies 6.3 kcal mol^–1^ higher in energy than **2b** (Figure 4b). The vacant site generated on the axial position enables the displacement of a chlorido into this coordination site. The transition state TS1-**2bc-dd** is reached when the angle between the aliphatic nitrogen and the migrating chlorido ligand is 139°, with a Gibbs free energy of 12.4 kcal mol^–1^. Subsequent rotation of the chlorido ligand to the axial position leads to the formation of the intermediate Int2-**2cb-dd**, which possesses an angle of 175° and lies at 5.8 kcal mol^–1^. Finally, recoordination of the sulfoxide ligand at the equatorial vacant site yields the **2c-Cl** isomer, which is 5.8 and 2.4 kcal mol^–1^ more stable than the intermediate Int2-**2cb-dd** and **2b**, respectively. This sulfoxide dissociation reaction pathway is more favored than the chlorido dissociation mechanism (see Figure S10).

### Photo- and Thermochemically Induced Processes

It is well established that ruthenium complexes containing dmso ligands undergo photo- and thermally induced transformations, mediated by the solvent and experimental conditions. Given their potential application in catalysis, evaluating the behavior of these compounds under such stimuli is of particular interest. Consequently, we investigated the response of isomers **2a** and **2c** to light exposure and thermal treatment across various media, including CH_2_Cl_2_, H_2_O, and CH_3_CN.

### Photochemical Studies

The photoinduced transformations of isomers **2a** and **2c** in the solvents mentioned above are displayed in Scheme 2. To the best of our knowledge, there are no reported studies on the photochemical behavior of [Ru^II^Cl_2_(N_3_)­(dmso-S)] complexes, where N_3_ denotes a tridentate nitrogen-donor ligand, in either noncoordinating solvents such as CH_2_Cl_2_ or coordinating solvents such as H_2_O or CH_3_CN. All the solutions were irradiated at room temperature using a 150 W xenon lamp (Hamamatsu L8253), equipped with a broadband 400–700 nm filter (λ = 400–700 nm). The evolution of the samples was monitored by UV–vis and/or CV experiments.

**Scheme 2:**
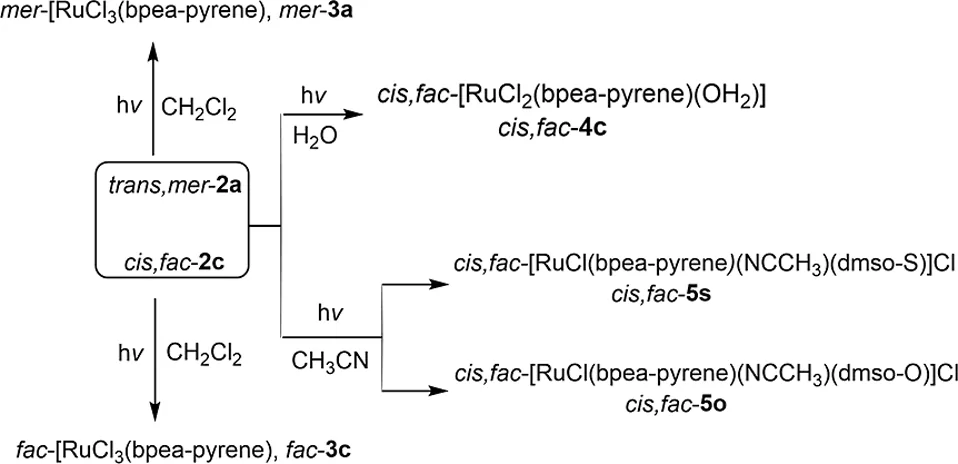
Photochemical Transformations of **2a** and **2c** in Different Solvents

Upon exposure to light, the colors of the solutions of **2a** and **2c** (0.1 mM) in CH_2_Cl_2_ change from light yellow to brown (for **2a**) or green (for **2c**), indicating the occurrence of light-induced processes. Their evolutions have been monitored through UV–vis experiments (Figure 5). For **2a**, after 15 min, the disappearance of the band at 430 nm corresponding to the MLCT occurred, with the subsequent emergence of two isosbestic points at 410 and 440 nm, indicating the formation of a new species (Figure 5a). For **2c**, isosbestic points were observed at 360 and 454 nm (Figure 5b). The UV–vis spectra of **2a** showed the appearance of new bands around 495 nm, consistent with the LMCT (Clpπ–Rudπ*) transition (Figure 5a).^66^ This band is less pronounced for **2c** due to the slower kinetics of the process (Figure 5b). This fact seems to indicate the substitution of dmso by a chlorido ligand and the subsequent oxidation of Ru­(II) to Ru­(III) with the formation of new complexes such as *mer*-[Ru^III^Cl_3_(bpea-pyrene)] *mer-* **3a** and *fac*-[Ru^III^Cl_3_(bpea-pyrene)] *fac-* **3c**. The formation of *mer-* **3a** was corroborated by the obtention of suitable crystals that were solved through X-ray diffraction analysis (Figure S11 and Table S1). It is worth noting that the oxidation and substitution process observed in **2a** preserves the *mer-* configuration without undergoing any type of isomerization. For both compounds, CV preformed after irradiation displayed a decrease of around 90 mV of the Ru­(III)/Ru­(II) redox couple (*E*_1/2_ around −0.30 V) with respect to the initial value, in accordance with the presence of a new electron-donating Cl ligand (see Figure S12).

**Figure 5:**
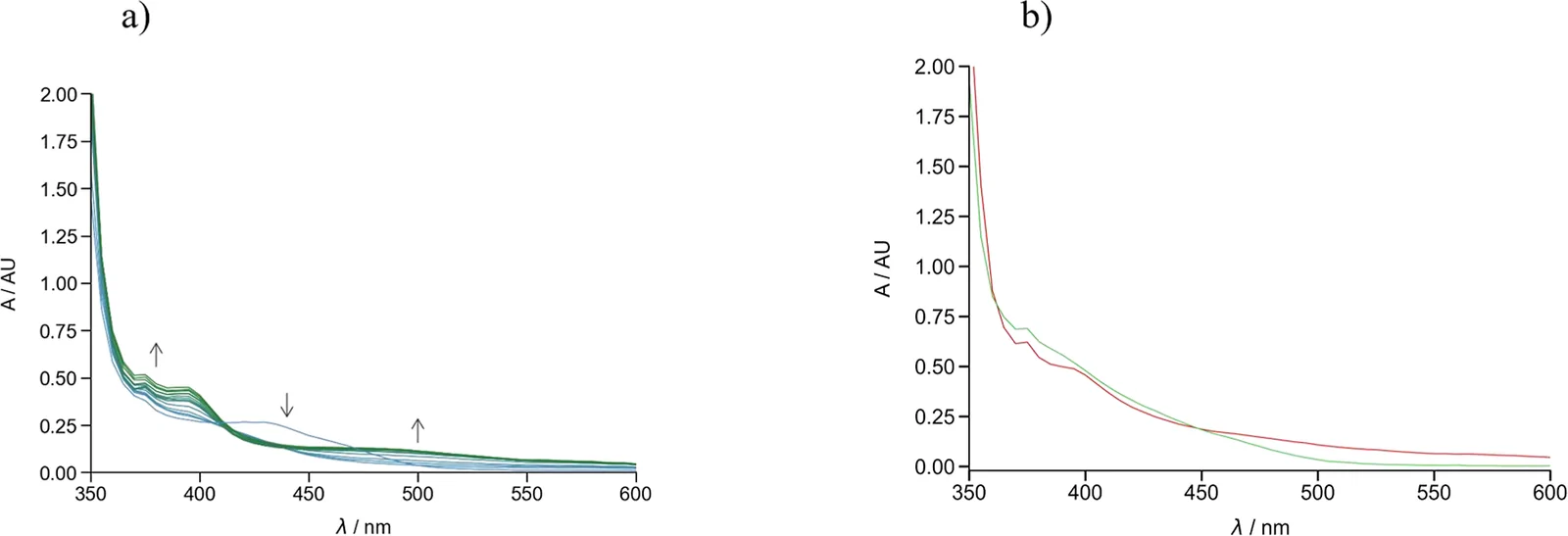
UV–vis spectra corresponding to the photochemical transformation of (a) *trans*,*mer*-**2a** (0.1 mM) and (b) *cis*,*fac-* **2c** (0.1 mM) (green *t* = 0 h; red *t* = 3 h) after 3 h upon light irradiation in CH_2_Cl_2_.

The UV–vis spectra recorded in solutions of **2a** and **2c** (0.1 mM) in H_2_O/acetone after irradiation are displayed in Figure S13. For **2a**, the spectrum shows the decrease and disappearance of the initial MLCT band at 410 nm after 10 min, accompanied by the emergence of a new band around 380 nm and the appearance of isosbestic points around 360, 395, and 440 nm, indicating the formation of a new species. For **2c**, the appearance of two isosbestic points around 370 and 480 nm, together with a new band around 380 nm, indicates the formation of the same compound as for **2a** (Figure S13b). This species presumably corresponds to the substitution of the dmso ligand by water, leading to the formation in both cases of *fac-*[Ru^II^Cl_2_(bpea-pyrene)­(OH_2_)], *cis,fac-* **4c** (Scheme 2). The electrochemical behavior of the water solution (phosphate buffer pH = 7) of **2a** after irradiation showed the formation of a new wave for the Ru­(III)/Ru­(II) couple around 0.00 V. This fact is in accordance with the substitution of a dmso ligand by one less electron-withdrawing water ligand.

Photoinduced processes were also investigated in the CH_3_CN solvent. The evolution of the CV of **2c** in CH_3_CN + 0.1 M TBAPF_6_ is shown in Figure 6. After 10 min of irradiation, the intensity of the wave at *E*_1/2_ = 0.60 V decreased slightly, accompanied by the emergence of new redox processes at *E*_1/2_ = 0.25 V, *E*_1/2_ = 0.85 V, and *E*_pa_ = 1.08 V. The potential at 0.85 V is consistent with the substitution of a Cl ligand by CH_3_CN, as the replacement of a dmso ligand would result in lower potential values. The new wave observed at 1.08 V corresponds to the oxidation of the chlorido ligand,^67^ in agreement with the formation of *cis*,*fac*-[RuCl­(bpea-pyrene)­(CH_3_CN)­(dmso-S)]­Cl, *cis*,*fac*-**5s**. The lower potential process observed at 0.25 V is consistent with the linkage isomerization of the dmso ligand and the formation of *cis*,*fac-*[Ru^II^Cl_2_(bpea-pyrene)­(dmso-O)], which may exist in equilibrium with the **2c** isomer. This isomerization is further supported by the UV–vis spectrum (Figure S14a), where a new band appears at 425 nm after 5 min, alongside the initial band at 400 nm. Coordination of the dmso ligand through oxygen leads to greater destabilization of the ruthenium d orbitals; consequently, a new band appears at longer wavelengths compared to the sulfur-bound analogue. Upon prolonged irradiation, the waves at *E*_1/2_ = 0.60 V and *E*_1/2_ = 0.25 V decrease, with a simultaneous growth of the wave at 0.85 V and the appearance of an additional wave at *E*_1/2_ = 0.76 V. This latter likely corresponds to the formation of the oxygen-coordinated complex *cis*,*fac*-[RuCl­(bpea-pyrene)­(NCCH_3_)­(dmso-O)]­Cl, *cis*,*fac*-**5o**. In both cases, the substitution of a chlorido ligand with acetonitrile takes place. A similar behavior was observed when **2a** was irradiated (Figure S14b).

**Figure 6:**
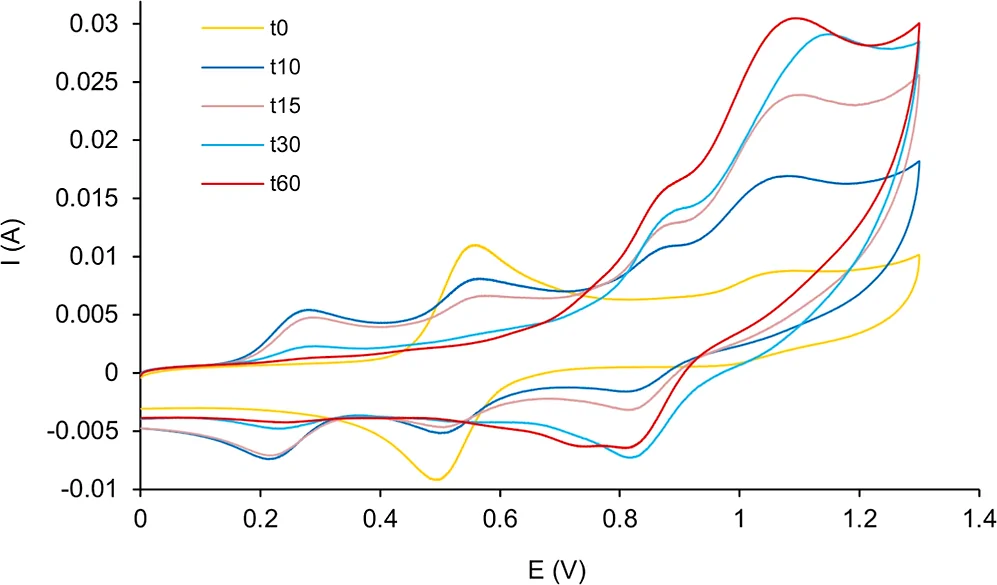
CV experiments corresponding to the photoinduced transformation of **2c** at RT in CH_3_CN + 0.1 M TBAPF_6_ vs SCE after 60 min.

### Thermal Reactivity Studies

Based on prior research, it is established that complexes containing the bpea ligand in the meridional configuration do not undergo thermal substitution in methanol or dmso. In contrast, these substitution processes are observed when the ligand is coordinated in a facial geometry.^49^ Consequently, we investigated the thermal behavior of isomers **2a** and **2c** in H_2_O and acetonitrile, in the dark; the resulting products are illustrated in Scheme 3. Figure 7a presents the spectral changes of a 0.1 mM solution of **2a** at 60 °C. The UV–vis spectra exhibit an MLCT band at 410 nm that progressively decreases over time, accompanied by the appearance of a new band at approximately 380 nm. After 4 h, the final spectrum matches the profile obtained for **2c** in water after irradiation (Figure S15b). These results suggest that *mer-*to *fac-*isomerization occurs, followed by the substitution of the dmso ligand by an aqua ligand to yield *cis*,*fac-* **4c**.

**Scheme 3:**
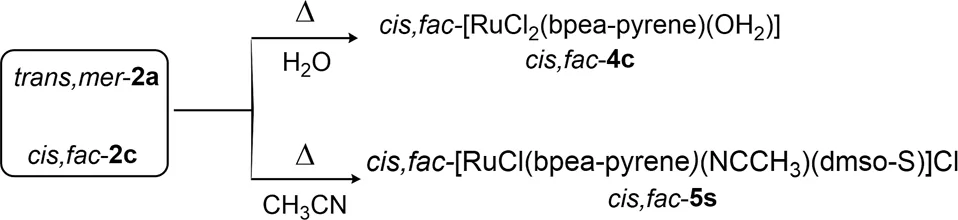
Thermoinduced Transformations of **2a** and **2c** in Different Solvents

**Figure 7:**
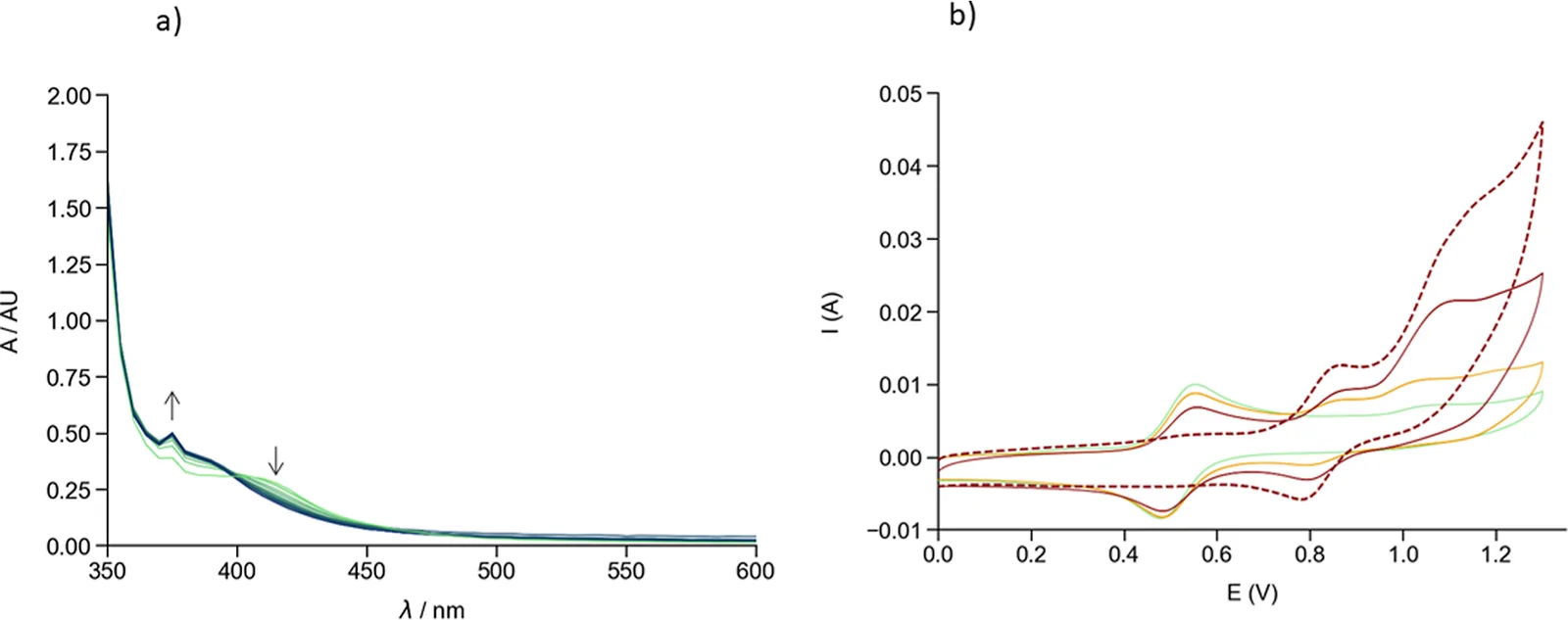
(a) UV–vis spectra corresponding to the thermal transformation of **2a** at 60 °C in H_2_O after 4 h; (b) CV experiments corresponding to the thermal transformation of **2c** at 60 °C in CH_3_CN + 0.1 M TBAPF_6_ vs SCE, after 4 h 30 min (*t* = 0 h green, 2 h orange, 4 h brown) and **2a** after 4 h (dashed brown).

Subsequently, the thermal behavior of **2c** was monitored at 60 °C in H_2_O over 6 h. As shown in Figure S15a, the spectral evolution is characterized by the emergence of a band at approximately 390 nm, indicating the substitution of the dmso ligand by an aqua ligand and the formation of *cis*,*fac-* **4c**. These observations suggest that the substitution process occurs more rapidly for the *mer-* isomer. The presence of free dmso in the reaction medium was also confirmed by NMR spectroscopy following the catalytic experiments (vide infra).

Thermoinduced processes were also monitored in CH_3_CN at 60 °C via CV experiments. As shown in Figure 7b, the intensity of the peak at *E*_1/2_ = 0.60 V for **2c** progressively decreases over time, accompanied by the emergence of a new redox process at *E*_1/2_ = 0.85 V. This shift at higher potential is consistent with the substitution of an anionic Cl ligand for CH_3_CN, since the replacement of a dmso ligand would lead to lower potential values. The new anodic wave observed at 1.08 V is tentatively assigned to the oxidation of the chloride ions released into solution upon substitution of the coordinated chlorido ligand by CH_3_CN,^67^ in accordance with the formation of *cis*,*fac*-[RuCl­(bpea-pyrene)­(NCCH_3_)­(dmso-S)]­Cl, *cis*,*fac*-**5s**. This assignment is supported by previous electrochemical studies in related coordination systems, in which the intensity of this wave increased upon addition of chloride ions to the electrolyte. Notably, similar behavior was observed for **2a** (Figure 9, dashed line); however, in this case, complete substitution of the Cl ligand occurs within 4 h, whereas the process is significantly slower for **2c**. Complex *cis*,*fac*-**5s** was successfully isolated, and its characterization is in full agreement with the species generated during this thermal process (Figure S16).

### Synthesis and Characterization of the Hybrid Material

#### Functionalization of GO with *cis*,*fac-* **2c**

The thermodynamically stable isomer **2c** was supported on graphene oxide (GO) in one step as shown in Scheme 4. The synthetic strategy consisted of the preparation of a dispersion of 50 mg of GO in water/acetone 9:1 that was sonicated for 30 min; afterward, 10 mg of **2c** was added. The dispersion was stirred for 24 h in the dark at RT to afford the immobilized complex GO@*cis*,*fac*-**2c** (GO@**2c**), which was filtered, washed with water, and dried under vacuum. The amount of ruthenium complex anchored onto GO, measured by inductively coupled plasma-atomic emission spectrometry (ICP-AES), was 8 μmol/100 mg of the catalyst. The immobilization of **2c** on GO is expected to be mainly driven by π–π interactions between the pyrene moiety and the GO surface. Although intermolecular π–π stacking between pyrene units cannot be completely ruled out, the relatively low amount of complex with respect to the available GO surface area, together with the extensive washing procedure, is expected to favor interactions of the pyrene moieties onto GO rather than pyrene–pyrene aggregation. To the best of our knowledge, this is the first example of a molecular dmso ruthenium complex anchored on GO support through noncovalent interactions using water as the solvent.

**Scheme 4:**
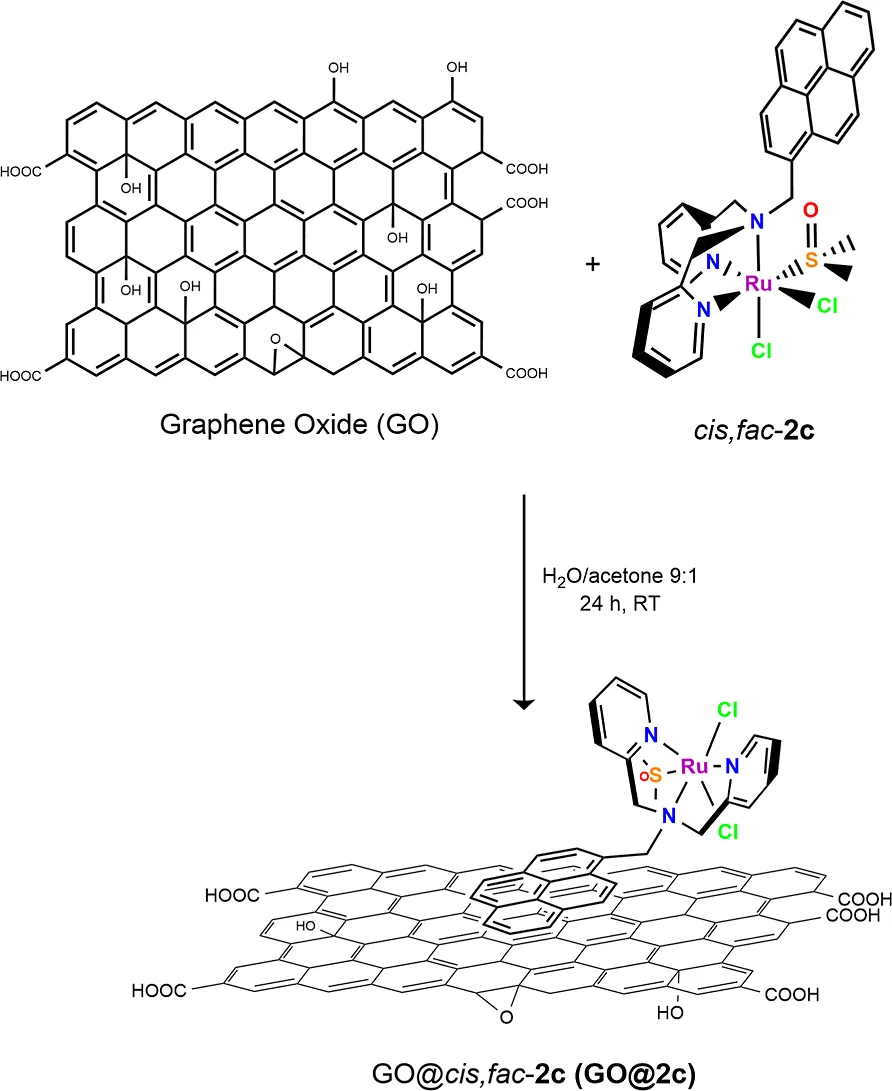
Synthetic Strategy Used for the Obtention of GO@**2c**

The hybrid material GO@**2c** was characterized using various techniques, such as thermogravimetric analysis (TGA), transmission electron microscopy (TEM), scanning electron microscopy (SEM), X-ray photoelectron spectroscopy (XPS), and differential pulse voltammetry (DPV), to further validate the anchorage.

TGA was performed for the naked GO support and for the anchored Ru complex (Figure S17). The support shows thermoxidative degradation with a total weight loss between 450 and 700 °C. For the functionalized support, we can observe a weight loss around 11% between 380 and 420 °C that corresponds to the evaporation of the adsorbed solvent and release of the ruthenium complex. A subsequent decomposition of around 55% between 420 and 700 °C corresponds to the degradation of the support, then leading to a stability phase above 700 °C, with a residue of 27%.

SEM and TEM images of GO@**2c** (Figures 8 and S18) reveal the morphology of the composite material, showing that the hybrid material consists of structures with rough surfaces and irregular shapes. Furthermore, backscattered electron (BSE) imaging (Figure 8b) confirms the presence of ruthenium on the GO, manifested as bright spots.

**Figure 8:**
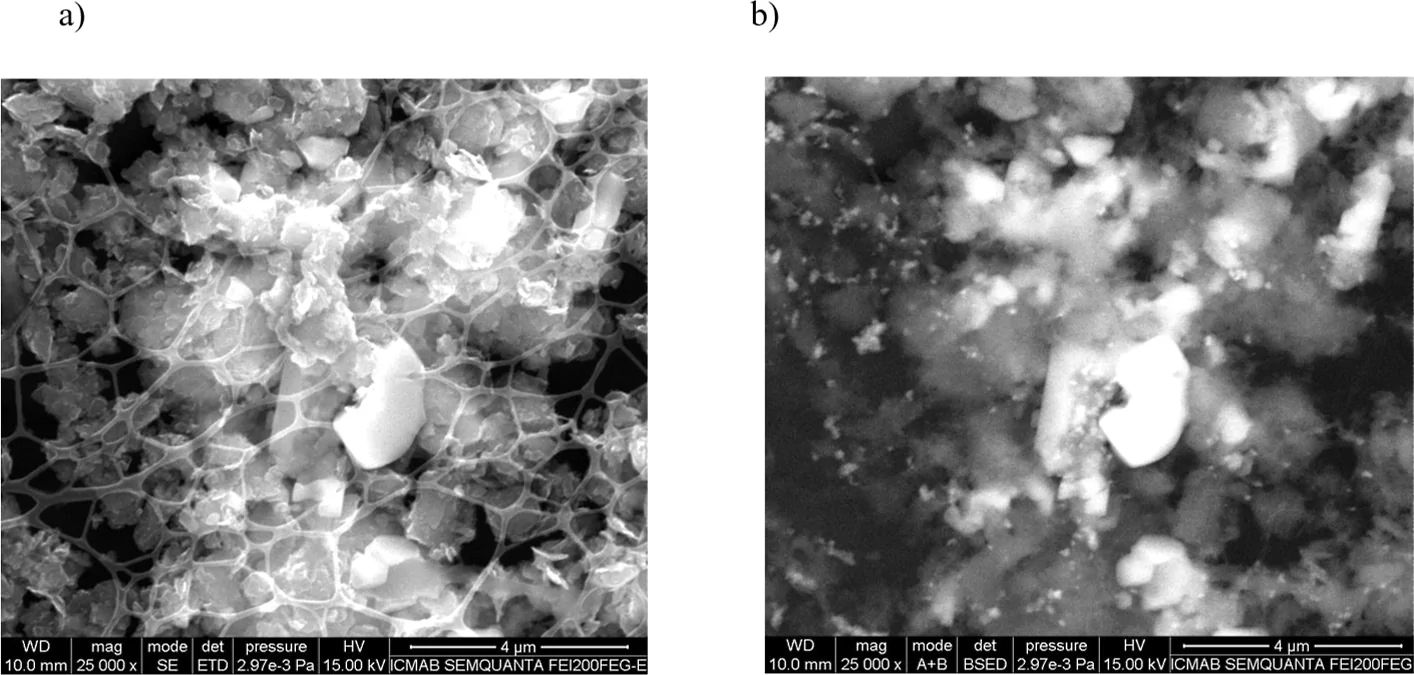
SEM images (a) using an ET detector and (b) using a BSE detector of GO@**2c**.

XPS analysis revealed that ruthenium, carbon, nitrogen, oxygen, chlorine, and sulfur are present on the surface of the GO@**2c** catalyst. These last two peaks show poor resolution because they are close to the detection limit of the instrument (Figure S19). The spectra display the strong peak of C 1s at ∼284 eV that corresponds to C=C, C–C, C–O moieties present in both support and complex. The small peak at ∼281 eV observed next to the carbon signal is attributable to Ru­(3d_5/2_).^68^ The peak of O 1s present in both the GO and complex appears at 532 eV, while the peak of N 1s present in the complex appears at ∼400 eV.

The electrochemical behavior of GO@**2c** was investigated via DPV, conducted on a suspension of the hybrid material in CH_2_Cl_2_ (0.1 M TBAPF_6_). DPV curves for the GO@**2c** hybrid alongside those of the homogeneous **2c** complex display a similar profile (Figure S20). GO@**2c** exhibits a redox wave at approximately 0.59 V vs SCE, assigned to the Ru­(III/II) couple. This potential reflects a 20 mV cathodic shift relative to that of the free **2c** complex. The observed cathodic shift is consistent with some degree of electronic interaction between the Ru complex and the GO. However, additional electronic structure characterization is required to determine whether charge transfer occurs. Furthermore, these results confirm the successful immobilization of **2c** on the GO surface.

### Catalytic Hydrolysis of Nitriles

The catalytic performance of the molecular complex **2c** and the hybrid heterogeneous GO@**2c** was evaluated in the hydrolysis of specific nitriles in water. For both catalysts, experiments were conducted using 2.5 mL of an aqueous solution containing the substrate and a catalyst loading of either 1 mol % or 5 mol %. Reactions involving complex **2c** were carried out at 100 °C for 20 h. After this period, the residual substrate was extracted with chloroform (using biphenyl as an internal standard) and quantified by means of GC analysis. The aqueous phase, containing the amide product, was further analyzed through ^1^H NMR spectroscopy (see Supporting Information). Table 1 summarizes the conversion, amide yield, and TOF values obtained for the different substrates. Overall, high conversions and excellent selectivity toward the corresponding amides were obtained. In all cases, the selectivity exceeded >98%. These selectivity values were confirmed by ^1^H NMR spectroscopy. Control experiments demonstrated that no significant hydrolysis occurred in the absence of the catalyst, under the same conditions, with the exception of chloroacetonitrile, for which, in the absence of a catalyst, around 40% conversion was observed.^27^

**Table 1:**
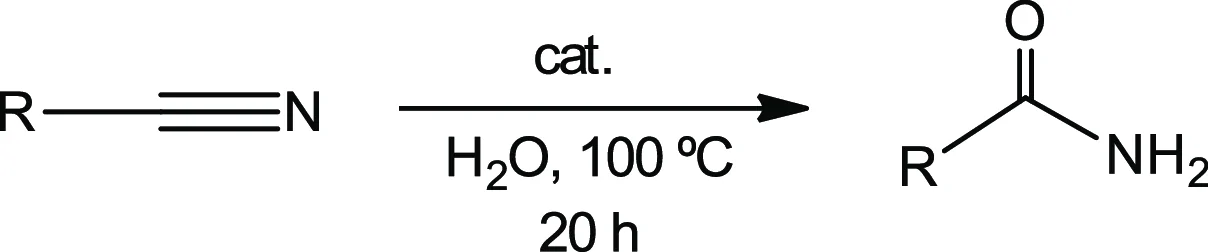

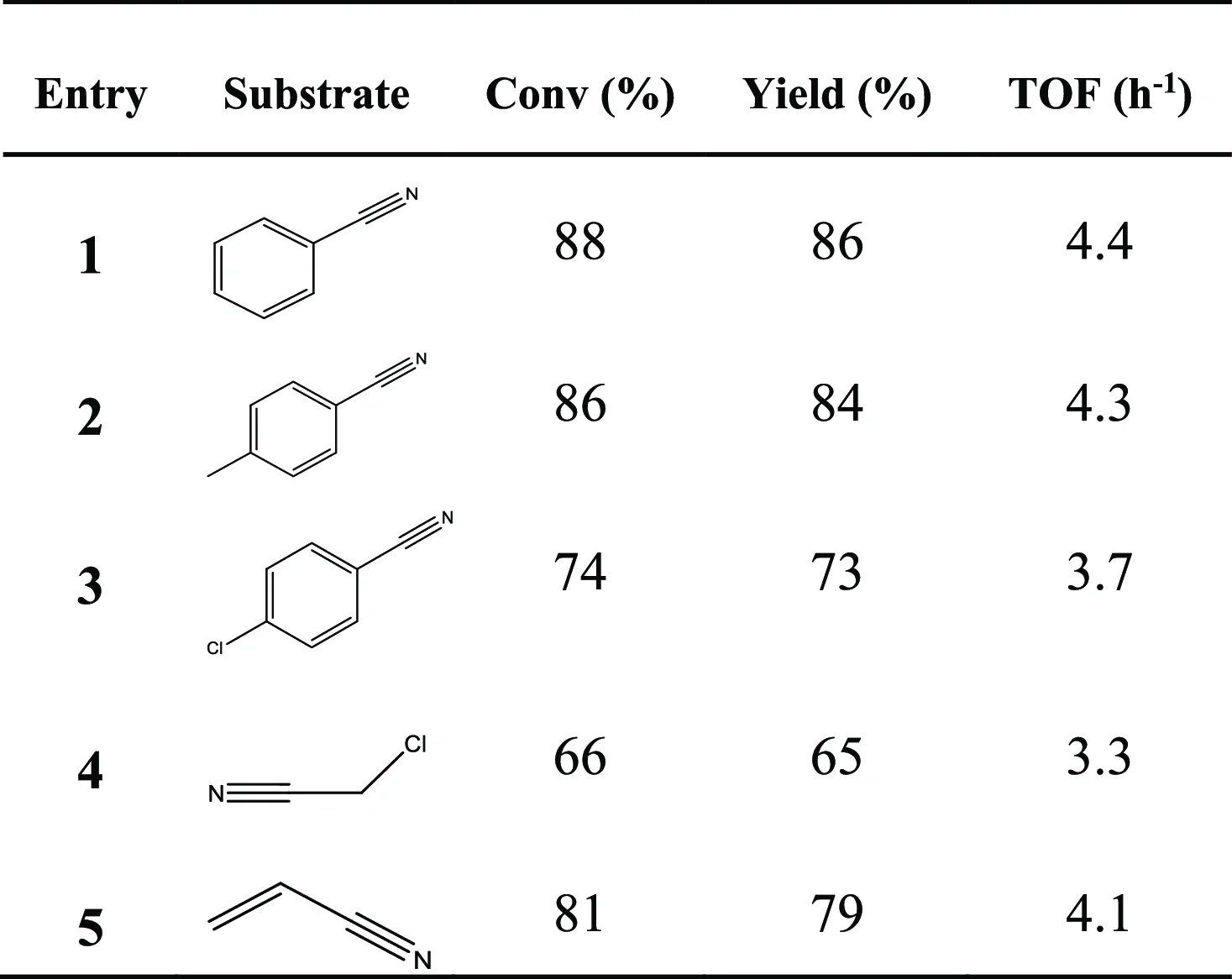
Ru-Catalyzed Hydrolysis of Nitriles to Amides in Water Using **2c** as the Catalyst^a^ ^,^ ^b^

a Conditions: reactions performed at 100 °C in the dark with a [substrate]/[Ru] ratio = 100:1.

b Selectivity = (amide yield/substrate conversion) × 100, in all cases >98.

The highest catalytic performance was observed in the hydrolysis of aromatic substrates, such as benzonitrile. This is likely due to the electron-withdrawing nature of the aromatic ring compared with aliphatic substrates like chloroacetonitrile and acrylonitrile, which exhibit weaker electron-withdrawing character and, consequently, lower yields. This electronic effect aligns with the established mechanism for nitrile hydrolysis, in which a ligand is replaced by the substrate in the metal coordination sphere, followed by a nucleophilic attack of water (or hydroxoanions) on the nitrile carbon atom. Lower conversions were noted for benzonitrile derivatives bearing either the *para*-electron-donating Me group or the *para*-electron-withdrawing Cl substituent. In the case of the latter, resonance delocalization of the lone pairs through the aromatic ring may account for the decreased activity. In comparison with the conversion and selectivity values reported for other [RuCl_2_N_2_(dmso)_2_] complexes,^27^ compound **2c** exhibited superior catalytic performance. This enhancement can be ascribed to improved substrate accessibility to the active site, facilitated by the lower steric profile of **2c** relative to that of analogues containing bulkier substituents near the ruthenium center.

The catalytic activity of the heterogeneous anchored system GO@**2c** was also investigated for the hydrolysis of benzonitrile and acrylonitrile in water. Using the same experimental conditions as the homogeneous system (1 mol % catalyst, 100 °C), benzonitrile reached a moderate conversion of 51%, whereas a significantly higher conversion was observed for acrylonitrile (92%). In both cases, high selectivity was maintained (>98%). Simultaneously, control experiments using pristine GO yielded conversions of 30% for benzonitrile and 39% for acrylonitrile. This background activity can be attributed to the presence of various oxygenated functional groups on the GO surface, such as hydroxyl (C–OH) and carboxylic acid (–COOH) groups, which act as nucleophiles toward the nitrile. Nevertheless, these results clearly demonstrate the significant catalytic enhancement of the GO support upon functionalization with our complex.

For acrylonitrile, an excellent conversion was achieved, significantly surpassing the results obtained in the homogeneous phase under identical conditions. In contrast, the conversion of benzonitrile was lower.

To investigate whether this difference could arise from preferential substrate adsorption on the graphene oxide surface, control adsorption experiments were performed using aqueous solutions of both nitriles and analyzed by UV–vis spectroscopy. No significant changes in substrate concentration were observed after equilibration with GO and GO@**2c**, suggesting that neither support exhibits appreciable adsorption of benzonitrile or acrylonitrile under the studied conditions (data not shown). Therefore, the lower conversion of benzonitrile is unlikely to be related to preferential adsorption on the graphene oxide surface. Although the origin of this difference remains to be fully elucidated, it may be related to the more sterically demanding approach of benzonitrile to the immobilized Ru active site compared to the smaller acrylonitrile molecule.

To optimize the efficiency of the catalytic process, the catalyst loading was increased to 5 mol % while keeping all other reaction parameters constant. The results are summarized in Table 2.

**Table 2:**
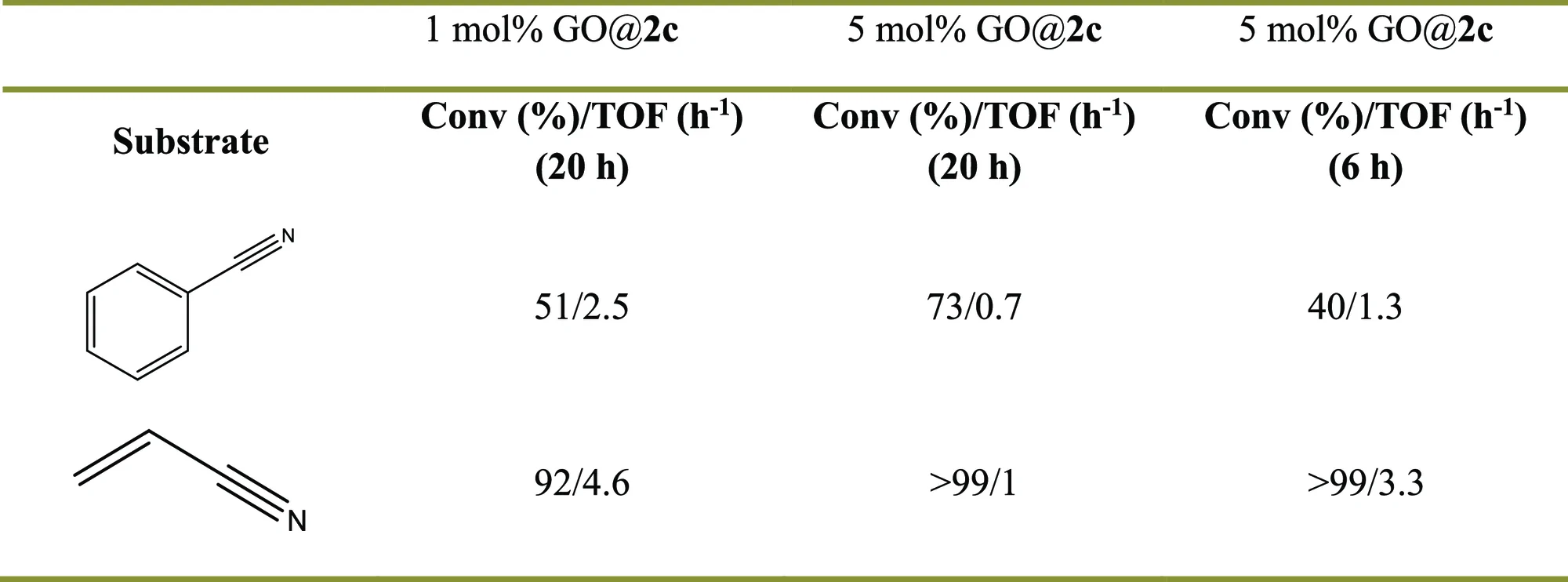
Ru-Catalyzed Hydrolysis of Nitriles to Amides in Water at 100 °C Using the Systems GO@**2c**

We can observe that a higher amount of catalyst leads to higher conversion values for both substrates, maintaining the selectivity values (>98%). Nevertheless, even at higher catalyst loadings, the conversion of benzonitrile remained lower than that achieved in the homogeneous phase.

Studies conducted at 5 mol % loading over a 6 h reaction time confirmed the same trend, showing high conversions for acrylonitrile but only moderate conversion for benzonitrile. These results indicate that the difference in catalytic performance between the two substrates persists under all of the conditions investigated. Since adsorption experiments rule out significant substrate adsorption on the support, further mechanistic studies will be required to establish the origin of this behavior.

As expected, the average TOF values calculated after 20 h are lower than those obtained after 6 h, reflecting the progressive decrease in the average reaction rate as the reaction proceeds, probably due to substrate consumption. On the basis of catalytic and thermal substitution studies, we propose a reaction mechanism (Figure 9) consistent with literature reports.^45^ The process begins with the activation of the ruthenium precursor in aqueous solution: complex **2c** undergoes the labilization and substitution of a dmso ligand by water, as evidenced by the detection of free dmso via NMR spectroscopy after catalysis (Figure S23a), followed by the replacement of a chlorido ligand by the nitrile substrate. Once coordinated, the metal center acts as a Lewis acid, polarizing the CN bond and enhancing its susceptibility to nucleophilic attack. Under neutral conditions, the coordinated water molecule plays a crucial role by organizing incoming solvent water molecules through hydrogen bonding, thereby facilitating the nucleophilic addition of water to an activated nitrile. This process yields a coordinated iminol intermediate, which subsequently undergoes dissociation and tautomerization to afford the final primary amide while regenerating the active catalytic species.

**Figure 9:**
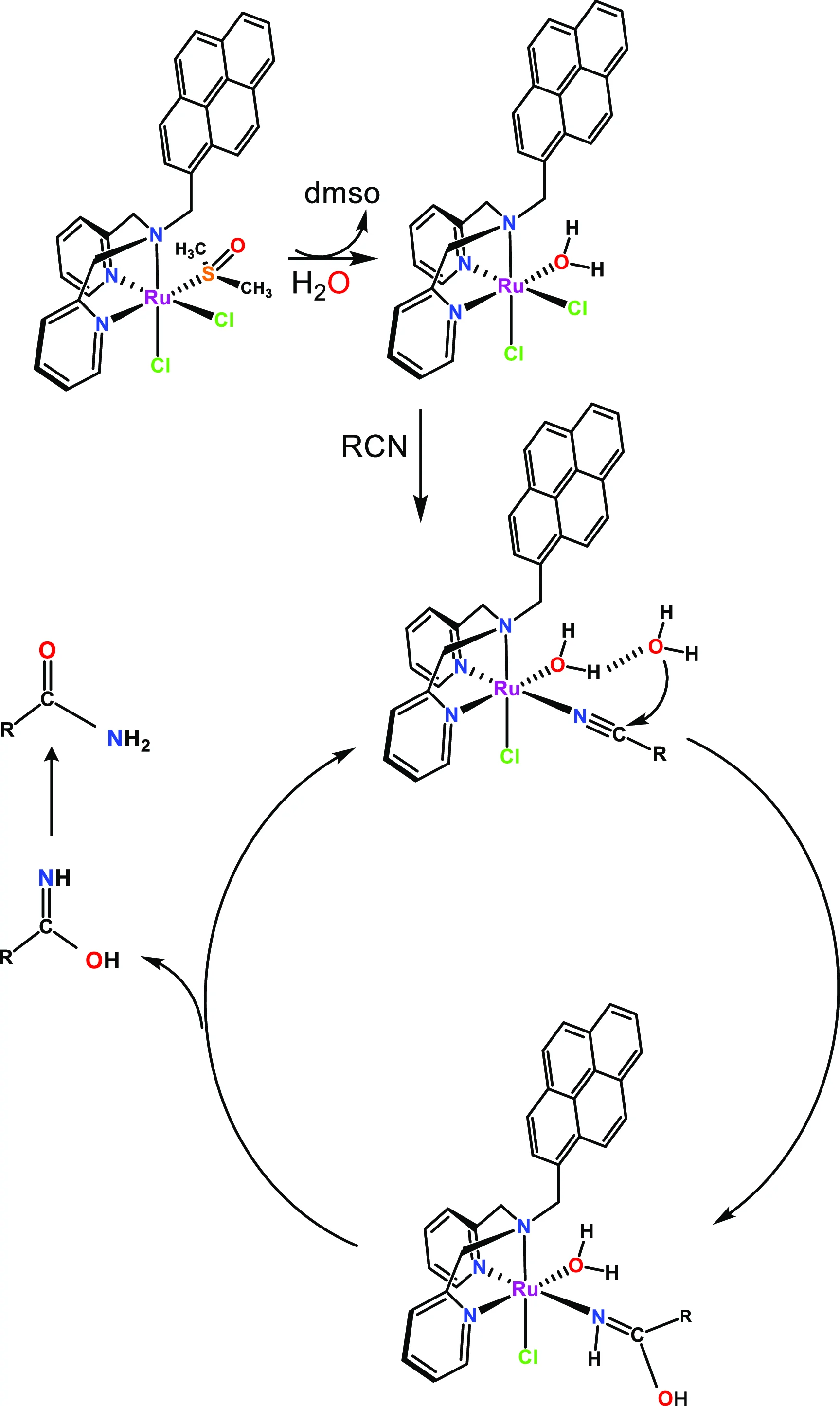
Suggested mechanism of nitrile hydrolysis by **2c**.

The reusability and structural stability of GO@**2c** were evaluated over five consecutive cycles for the hydrolysis of both substrates. Experiments were conducted in water using 5 mol % catalyst with reaction times of 6 and 20 h for benzonitrile and 6 h for acrylonitrile. After each run, the reaction mixture was cooled, the catalyst was recovered by centrifugation, washed with water, dried, and reused under identical conditions. As shown in Figures 10 and S21, the catalyst retained high hydrolysis effectiveness, conversion, and selectivity throughout all cycles. The lack of significant variation in conversion suggests that ruthenium leaching is negligible, confirming that the hydrolysis process proceeds via a strictly heterogeneous pathway.

**Figure 10:**
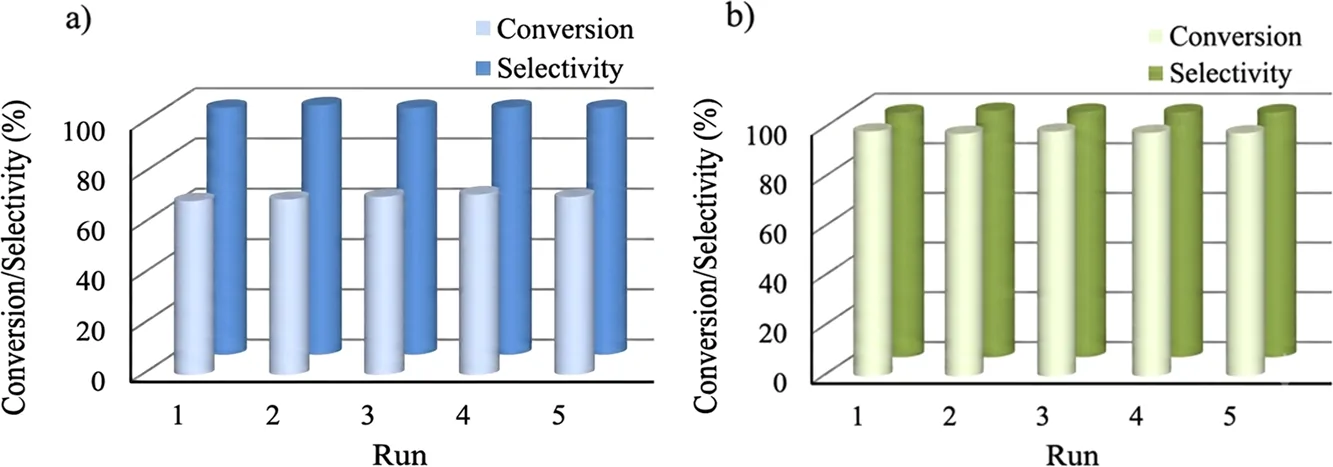
Conversion and selectivity values obtained throughout five consecutive reuses of GO@**2c** in the hydrolysis in water of (a) benzonitrile after 20 h and (b) acrylonitrile after 6 h.

To assess its structural integrity, the catalyst was examined by SEM and TEM following five cycles of benzonitrile hydrolysis (Figure S22). The micrographs showed no significant morphological changes, confirming that the material’s structure remained intact during repeated use. Furthermore, ICP analysis of the reaction supernatants after successive reuses showed no detectable traces of ruthenium, corroborating the absence of metal leaching. These results confirm that GO@**2c** is a robust and reusable catalyst. Its sustained performance, structural resilience, and negligible metal loss position it as a promising candidate for the sustainable hydrolysis of nitriles in aqueous media.

## Conclusions

In summary, we have successfully synthesized and performed a total spectroscopic, structural, and electrochemical characterization of three distinct Ru­(II)-dmso isomers, *trans*,*mer*-**2a** (**2a**), *trans*,*fac*-**2b** (**2b**), and *cis*,*fac*-**2c** (**2c**), derived from the [Ru­(II)­Cl_2_(bpea-pyrene)­(dmso)] complex. All characterized species incorporate the N-tridentate ligand 1-[*bis*(pyridine-2-ylmethyl)­amino]­methylpyrene (bpea-pyrene). Isomerization studies and DFT calculations confirm that **2c** is the thermodynamically stable isomer, in accordance with the experimental results. Beyond thermodynamic stability, DFT calculations elucidate the full isomerization network, identifying energetically feasible pathways involving sequential ligand dissociation and rearrangement steps that rationalize the experimental formation of **2b** and its subsequent conversion to **2c**.

The **2a** and **2c** isomers exhibit distinct photo- and thermoinduced reactivity dictated by their stereochemistry and the solvent medium. Irradiation triggers ligand exchange and, specifically in dichloromethane, Ru-oxidation. In contrast, thermal activation requires a preliminary *mer*-to-*fac-*isomerization. Solvent coordination properties further modulate the substitution pathways: water facilitates the replacement of dmso by H_2_O, while MeCN induces Cl^–^ substitution and, under light irradiation, dmso isomerization. The results highlight the configurational stability and selective reactivity of these complexes, providing insights that are relevant to their potential catalytic applications. The **2c** isomer was successfully immobilized onto graphene oxide (GO) through noncovalent interactions. GO@**2c** underwent comprehensive characterization using both spectroscopic and electrochemical methods.

Molecular complex **2c** and its heterogeneous counterpart GO@**2c** have proven to be efficient catalysts for the hydrolysis of nitriles in water, achieving high conversion and selectivity toward the corresponding amides. The catalytic activity is strongly influenced by the electronic nature of the substrates, with aromatic nitriles generally showing better performance.

GO@**2c** exhibits robust catalytic activity, particularly toward acrylonitrile, outperforming its homogeneous analogue under identical reaction conditions. Increasing the catalyst loading to 5 mol % further enhanced conversion rates without compromising selectivity. Furthermore, the heterogeneous catalyst demonstrated excellent reusability and structural integrity over five consecutive cycles. These findings underscore the potential of GO@**2c** as a stable and sustainable catalyst for the aqueous hydrolysis of nitriles, effectively combining high efficiency with long-term durability. Future work will further explore its applicability to a broader range of aromatic and aliphatic nitriles. Finally, a reaction mechanism is proposed on the basis of catalytic and thermal substitution studies.

## Supplementary Material



## Data Availability

A data set collection of computational results is available in the ioChem-BD repository and can be accessed at DOI: 10.19061/iochem-bd-6-657.^69^

## Notes

Experimental procedures; crystal and structure determination data; FT-IR, UV–vis, NMR, XPS, and ESI mass spectra; TEM and SEM images; TGA; and electrochemical, catalytic, and computational data (PDF)
