# Supplementary material for: Graphene Oxide-Anchored Ru-dmso Catalyst for Sustainable Nitrile Hydrolysis in an Aqueous Medium
Source: Inorg Chem. 2026 Sep 3;65(37):21700–15. doi: 10.1021/acs.inorgchem.6c02958 (PMC13599385; doi:10.1021/acs.inorgchem.6c02958)
Supplement: Supplementary file 1 [file ic6c02958_si_001.pdf]

SUPPORTING INFORMATION

Graphene Oxide-Anchored Ru-dmso Catalyst for Sustainable Nitrile Hydrolysis in an Aqueous Medium

Izan Roca,[a] Nabila Mimouni,[a] Syrine Affès,[a] Xavier Fontrodona,[a] Josep M.Luis[b] and Isabel Romero[a] *

[a] Departament de Química and Serveis Tècnics de Recerca, Universitat de Girona, C/ M. Aurèlia Capmany, 69, E-17003 Girona, Spain.
[b] Institut de Química Computacional I Catàlisi (IQCC) and Departament de Química, Universitat de Girona, E-17003 Girona, Catalonia, Spain.

*Authors to whom correspondence should be addressed. I.R.: marisa.romero@udg.edu;

Structural characterization

Table S1. Crystallographic data for the X-ray diffraction of **2a**, **2b**, **2c** and *mer*-**3a**.

Table S2. Selected bond lengths (Å) and angles (°) for **2a**, **2b**, **2c** and *mer*-**3a**.

Table S3. Absolute electronic and Gibbs free energies and relative Gibbs free energies for all the isomers evaluated with different DFT functionals at 353.15 K, incorporating quasi-harmonic corrections.

Scheme S1. Possible isomers for [$Ru^{II}Cl_2$(bpea-pyrene)(dmso)].

Figure S1. a) Molecular structures of the enantiomers in the isomer **2c**; b) Hydrogen bond interactions for **2a** (1); **2b** (2) and **2c** (3). Circles in grey correspond to C atoms; in yellow to H atoms, in green to Cl atoms, in orange to S atoms, in red to O atoms, in blue to N atoms and, in greenish blue to Ru atom.

Figure S2. Packing arrangement for a) **2a** along axis a (1), b (2) and c (3); b) **2b** along axis a (4), b (5) and c (6) and c) **2c** along axis a (7) and c (8).

Figure S3. IR spectra of a) **2a** (blue); **2b** (red) and **2c** (green) and b) *cis,fac*-**5s**.

Figure S4. NMR spectra of **2a**, 400 MHz, CD_2Cl_2: a) ^{1}H-NMR; b) COSY; c) NOESY; d) ^{1}H-^{13}C HSQC.

Figure S5. NMR spectra of **2b**, 400 MHz, CD_2Cl_2: a) ^{1}H-NMR; b) COSY; c) ^{13}C-NMR; d)^{1}H-^{13}C HSQC.

Figure S6. NMR spectra of **2c**, 400 MHz, CD_2Cl_2: a) ^{1}H-NMR; b) ^{13}C-NMR; c) COSY; d) NOESY; e) ^{1}H-^{13}C HSQC; f) ^{1}H-^{13}C HMBC.

Figure S7. a) UV-vis spectra of **2a** (0.1 mM, blue line), **2b** (0.05 mM red line) and **2c** (0.05 mM green line) in MeOH. b) UV-vis spectrum of *fac*-**5s** (0.05 mM). c) UV-Vis spectrum of bpea-pyrene ligand in MeOH (0.05 mM).

Figure S8. a) CVs for complexes **2a,** (red), **2b** (blue) and **2c** (yellow) in CH_2Cl_2 + 0.1 M $TBAPF_6$ *vs* SCE (reference electrode). Glassy carbon electrode (3 mm diameter) was used as working electrode. b) Cyclic voltammetry for the isomer **2a** in CH_2Cl_2 + 0.1M $TBAPF_6$ vs. SCE, starting the scanning at E_{init} = 1.20 V applying an equilibration time of 2 s (red) and 20 s (blue).

Figure S9. Gibbs free energy profile of the proposed mechanism for the isomerization of *trans,mer*-**2a** to *trans,fac*-**2b** via sulfoxide dissociation computed at ωB97XD/def2-TZVP(H,C,N,O,S,Ru)/def2-TZVPD(Cl)//B3LYP/def2-SVP(H,C,N,O,S,Ru)/def2-SVPD(Cl) level of theory, including solvent effects (ethanol) and quasi-harmonic enthalpic and entropic corrections at 353.15 K (in kcal mol^{-1}).

Figure S10. Gibbs free energy profile of the proposed mechanism for the isomerization of *trans,fac*-**2b** to *cis,fac*-**2c** isomer via chloride dissociation computed at ωB97XD/def2-TZVPD(H,C,N,O,S,Ru)/def2-TZVPD(Cl)//B3LYP/def2-SVP(H,C,N,O,S,Ru)/def2-SVPD(Cl) level, including solvent effects (ethanol) and quasi-harmonic enthalpic and entropic corrections at 353.15 K (in kcal/mol^{-1}).

Figure S11. Molecular structure and labelling schemes for *mer*-**3a**.

Figure S12. Cyclic voltammetry preformed after irradiation of a) **2a** and b) **2c** in CH_2Cl_2 + 0.1 M $TBAPF_6$ vs. SCE, starting the scanning at E_{init} = 1.20 V.

Figure S13. a) UV-vis spectra corresponding to the photochemical transformation of **2a** upon 3 h of light irradiation and b) **2c** after 2.5 h (green t= 0 h; violet t= 2.5 h)**,** in H_2O; c) CV corresponding to the photochemical transformation **2a** after 3 h of irraciation in phosphate tampon pH 7(t= 0 h green line; t= 4 h blue line).

Figure S14. a) UV-vis experiment corresponding to the photoinduced transformation of **2a** at RT in CH_3CN after 60 min; b) CV corresponding to the photoinduced transformation of **2a** at RT in CH_3CN + 0.1 M $TBAPF_6$ vs. SCE, after 60 min.

Figure S15. a) UV-vis spectra corresponding to the thermal transformation of **2c** in H_2O at 60 ºC after 6 h; b) Superposition of UV-vis spectra resulting from photo and thermo substitution in water of **2c** and **2a**, respectively, leading to the formation of the compound *cis,fac*-[$Ru^{II}Cl_2$(bpea-pyrene)(OH_2)], *cis,fac*-**4c**.

Figure S16. CV experiment in CH_3CN+ 0.1 M $TBAPF_6$ vs. SCE and ESI-MS of *cis,fac*-**5s**.

Figure S17. Thermogravimetric analysis of naked GO (black line) and GO@**2c** (yellow line).

Figure S18. TEM images of GO@**2c**. The bars indicate a length of 100 nm.

Figure S19. a) XPS spectra of GO@**2c**; Magnification of the XPS spectrum in b) the O1s, c) the N1s and d) the C1s regions.

Figure S20. DPV of a) **2c** (blue line), GO@**2c** (red line) and GO (green line) and b) GO@**2c** (red line) and GO (green line) in CH_2Cl_2 +$TBAPF_6$ 0.1 M *vs* SCE (reference electrode). Glassy carbon electrode (3 mm diameter) was used as working electrode.

Figure S21. Conversion and selectivity values obtained throughout five consecutive reuses of GO@**2c** in the hydrolysis of benzonitrile in water after 6 h.

Figure S22. a) TEM and b) SEM images of GO@**2c** in the hydrolysis of benzonitrile after five reuses. The bars indicate a length of a) 200 nm; b) 10 µm.

Figure S23. Representative NMR spectra in $(CD_3)_2CO$ of the obtained amides in the hydrolysis of nitriles in water, a) benzamide; b) methylbenzamide; c) chlorobenzamide; d) acrylamide; e) chloroacetonitrile.

Structural characterization

Isomers **2a** and **2c** crystallize in the monoclinic system (space group P1 21/c1 and P 1 21 1, respectively) whereas **2b** crystallizes in the triclinic system (space group P-1). The **2c** isomer forms a racemic crystal, containing both enantiomers (Figure S1a). All three structures feature octahedrally coordinated Ru(II) centers bound to a N-tridentate bpea-pyrene ligand, two monodentate chlorido ligands and one monodentate dmso ligand. In **2a** the bpea-pyrene ligand adopts a meridional coordination mode, with the aliphatic nitrogen, N_{al}, *trans*- to the dmso ligand and the two aromatic nitrogens, N_{ar} arranged *trans*- to each other. The chlorido ligands are also positioned trans to one another, confirming the solid-state formation of the **2a** isomer. In **2b** and **2c** the bpea-pyrene ligand adopts a facial coordination mode, with both Cl ligands located *cis*- to each other. In **2b** the Cl ligands are located *trans*- to the N_{ar}, while the dmso ligand is *trans*- to the N_{al}. In contrast, in **2c** one Cl is *trans*- to one N_{ar} and the other is *trans*- to the N_{al}, placing the dmso ligand *trans*- to the remaining N_{ar}. All isomers exhibit shorter Ru-N_{ar} bond distances compared to Ru-N_{al} bonds, consistent with the differing electronic nature of the nitrogen atoms.[1] For example, in **2b** the Ru–N bond lengths are: Ru-N1, 2.075 Å, Ru-N2, 2.080 Å and Ru-N3, 2.171 Å. These bond lengths are within the expected range for similar ruthenium complexes reported in the literature.[2] Hydrogen bond interactions have been observed (Figure S1b). In **2a** and **2b** hydrogen bonds are formed between the O1 atom of the dmso ligand and the H1 atom of the pyridine ring of the bpea-pyrene ligand, with H1-O1 distances of 2.319 Å and 2.291 Å, respectively. In **2c**, a strong hydrogen bond is observed between O1 and the methylene hydrogen H7A (H7A-O1, 2.246 Å). Additional weak hydrogen bonds are found between Cl2 and the methylene hydrogen atoms of the bpea-pyrene ligand: H7A-Cl2, 2.671 Å in **2a**; H7B-Cl2, 2.616 Å in **2b** and H7B-Cl2, 2.698 Å in **2c**. In **2b**, interactions are also observed between Cl1 and the methylene hydrogen atom H8A (H8A-Cl1, 2.705 Å) and between a methyl hydrogen from the dmso ligand (H15A-Cl1, 2.649 Å). For **2c**, additional hydrogen bonds are observed between Cl1 and pyridine ring hydrogens H1 and H13 (H1-Cl1, 2.708 Å; H13-Cl1, 2.740 Å), as well as with H14F of the dmso methyl group (H14F-Cl1, 2.728 Å). The three-dimensional molecular arrangements of the isomers display intermolecular π-stacking interactions involving the pyrene substituents of neighboring bpea-pyrene ligands. These packing arrangements differ for each isomer (Figure S2).

Table S1. Crystallographic data for the X-ray diffraction of **2a**, **2b** complex, **2c** and *mer*-**3a**.

	trans,mer-**2a**	*trans-fac*-**2b**	*cis-fac*-**2c**	*mer*-**3a**
Empirical formula	$C_{31}H_{29}Cl_2N_3ORuS.CH_2Cl_2$	$C_{31}H_{29}Cl_2N_3ORuS$	$C_{31}H_{29}Cl_2N_3ORuS.CH_2Cl_2$	$C_{29}H_{23}Cl_3N_3Ru.CHCl_3$
Formula weight	748.53 g/mol	663.60 g/mol	748.53 g/mol	740.29 g/mol
Crystal system	Monoclinic	Triclinic	Monoclinic	Triclinic
Space group	P 1 21/c1	P-1	P 1 21 1	P-1
a[Ǻ]	22.417(3)	7.4598(6)	9.417(10)	7.922(5)
b[Ǻ]	8.634(14)	13.2501(11)	24.478(2)	13.489(9)
c[Ǻ]	15.517(2)	14.6411(12)	13.663(15)	14.339(10)
α[º]	90	108.860(2)	90	76.871(2)
β[º]	97.114(5)	95.049(2)	97.544(4)	80.979(2)
γ[º]	90	94.570(2)	90	84.561(2)
V [$Ǻ^3$]	2980.2(8)	1355.16(19)	3122.0(6)	1470.90(17)
Formula Units/ cell	4	2	4	2
Temp. [K]	100(2)	100(2)	100(2)	100(2)
ρ_{calc}, [g/cm^{-3}]	1.668	1.626	1.592	1.671
μ[mm^{-1}]	0.988	0.885	0.943	1.105
Final R indices, [I>2σ(I)]	R_1 = 0.1807<br>wR_2 = 0.3470	R_1 = 0.0482<br>wR_2 = 0.1065	R_1 = 0.0558<br>wR_2 = 0.1083	R_1 = 0.0522<br>wR_2 = 0.1192
R indices [all data]	R_1 = 0.1997<br>wR_2 = 0.3556	R_1 = 0.0795<br>wR_2 = 0.1284	R_1 = 0.0602<br>wR_2 = 0.1098	R_1 = 0.0774<br>wR_2 = 0.1350

Table S2. Selected bond lengths (Å) and angles (°) for **2a**, **2b**, **2c** and *mer*-**3a**.

	trans,mer-**2a**	*trans,fac*-**2b**	*cis,fac*-**2c**	*mer*-***3a***	
Ru(1)-N(1)	2.082(13)	2.075(4)	2.100(7) /2.105(7)	Ru(1)-N(5)	2.055(4)
Ru(1)-N(2)	2.124(13)	2.080(4)	2.132(6) /2.129(6)	Ru(1)-N(12)	2.118(4)
Ru(1)-N(3)	2.086(13)	2.171(3)	2.053(6) /2.054(7)	Ru(1)-N(19)	2.062(4)
Ru(1)-S(1)	2.243(4)	2.2154(12)	2.223(2) /2.220(2)	Ru(1)-Cl(2)	2.3353(11)
Ru(1)-Cl(1)	2.422(4)	2.4300 (12)	2.439(2) /2.425(19)	Ru(1)-Cl(3)	2.3709(11)
Ru(1)-Cl(2)	2.405(4)	2.4172(11)	2.414(2) /2.409(19)	Ru(1)-Cl(4)	2.3707(11)
N(1)-Ru(1)-N(2)	81.4(5)	94.63(14)	81.7(3)/81.0(3)	N(5)-Ru(1)-N(19)	163.14(15)
N(1)-Ru(1)-N(3)	161.4(5)	81.09(13)	80.9(3)/82.5(3)	N(19)-Ru(1)-N(12)	81.86(14)
N(2)-Ru(1)-N(3)	80.0(5)	78.51(13)	82.8(3)/82.9(3)	N(5)-Ru(1)-N(12)	81.37(14)
N(1)-Ru(1)-S(1)	96.6(4)	99.60(11)	172.8(2)/175.1(2)	N(5)-Ru(1)-Cl(2)	89.44(11)
N(2)-Ru(1)-S(1)	174.2(4)	96.81(10)	96.16(18)/96.95(19)	N(19)-Ru(1)-Cl(2)	90.47(10)
N(3)-Ru(1)-S(1)	102.0(4)	175.32(9)	92.0(2)/92.9(2)	N(5)-Ru(1)-Cl(4)	97.58(11)
N(1)-Ru(1)-Cl(2)	91.5(4)	83.76(10)	90.1(2)/88.6(2)	N(12)-Ru(1)-Cl(4)	177.01(10)
N(2)-Ru(1)-Cl(2)	94.9(4)	172.96(10)	90.33(19)/90.80(19)	N(5)-Ru(1)-Cl(3)	90.60(11)
N(3)-Ru(1)-Cl(2)	88.9(4)	94.47(9)	169.36(19)/169.8(2)	N(12)-Ru(1)-Cl(3)	89.55(10)
S(1)-Ru(1)-Cl(2)	90.68(13)	90.21(4)	96.86(8)/95.87(7)	Cl(4)-Ru(1)-Cl(3)	87.66(4)
N(1)-Ru(1)-Cl(1)	88.8(4)	169.49(11)	92.6(2)/92.82(19)	N(12)-Ru(1)-Cl(2)	95.60(10)
N(2)-Ru(1)-Cl(1)	87.5(4)	90.51(10)	174.29(18)/173.75(19)	N(19)-Ru(1)-Cl(4)	99.26(11)
N(3)-Ru(1)-Cl(1)	91.5(4)	90.99(9)	95.40(18)/95.3(2)	Cl(2)-Ru(1)-Cl(4)	87.18(4)
S(1)-Ru(1)-Cl(1)	86.97(14)	88.86(4)	89.31(8)/89.11(7)	N(19)-Ru(1)-Cl(3)	91.00(10)
Cl(2)-Ru(1)-Cl(1)	177.65(14)	90.03(4)	90.64(7)/90.10(7)	Cl(2)-Ru(1)-Cl(3)	174.80(4)

Table S3. Absolute electronic and Gibbs free energies and relative Gibbs free energies for all the isomers evaluated with different DFT functionals/basis sets at 353.15 K, incorporating quasi-harmonic enthalpic and entropic corrections.

	B3LYP/def2-SVP(H,C,N,O,S,Ru)/def2-SVPD(Cl)			B3LYP/def2-SVP(H,C,N,O,S,Ru)/def2-SVPD(Cl) Quasiharmonic corrections at 353.15 K		
**Isomer**	**E (a.u)**	**G (a.u)**	**ΔG (kcal mol^{-1})**	**E (a.u)**	**G (a.u)**	**ΔG (kcal mol^{-1})**
cis,mer-**2dd**	-2850.738814	-2850.26429	11.4	-2850.738814	-2850.2825	11.5
cis,mer-**2du**	-2850.747834	-2850.27254	6.2	-2850.747834	-2850.290964	6.2
trans,mer-**2a**	-2850.748278	-2850.27355	5.6	-2850.74828	-2850.292081	5.5
trans,fac-**2b**	-2850.753379	-2850.27915	2.0	-2850.753379	-2850.297295	2.2
cis,fac-**2c-S**	-2850.756668	-2850.28232	0.1	-2850.756668	-2850.300799	0.0
cis,fac-**2c-Cl**	-2850.755546	-2850.28242	0.0	-2850.755546	-2850.300576	0.1

	ωB97X-D/def2-SVP(H,C,N,O,S,Ru)/def2-SVPD(Cl)//B3LYP/def2-SVP(H,C,N,O,S,Ru)/def2-SVPD(Cl)			M06-2X/def2-SVP(H,C,N,O,S,Ru)/def2-SVPD(Cl)//B3LYP/def2-SVP(H,C,N,O,S,Ru)/def2-SVPD(Cl)		
**Isomer**	**E (a.u)**	**G (a.u)**	**ΔG (kcal mol^{-1})**	**E (a.u)**	**G (a.u)**	**ΔG (kcal mol^{-1})**
cis,mer-**2dd**	-2850.027782	-2849.571515	12.3	-2849.690527	-2849.234260	13.0
cis,mer-**2du**	-2850.037161	-2849.580291	6.8	-2849.700503	-2849.243633	7.1
trans,mer-**2a**	-2850.037737	-2849.581538	6.0	-2849.702221	-2849.246022	5.6
trans,fac-**2b**	-2850.042613	-2849.586529	2.9	-2849.706597	-2849.250513	2.8
cis,fac-**2c-S**	-2850.047013	-2849.591144	0.0	-2849.710873	-2849.255004	0.0
cis,fac-**2c-Cl**	-2850.045917	-2849.590946	0.1	-2849.709248	-2849.254277	0.5

	B3LYP/def2-TZVP(H,C,N,O,S,Ru)/def2-TZVPD(Cl) //B3LYP/def2-SVP(H,C,N,O,S,Ru)/def2-SVPD(Cl) SMD			ωB97X-D/def2-TZVP(H,C,N,O,S,Ru)/def2-TZVPD(Cl)//B3LYP/def2-SVP(H,C,N,O,S,Ru)/def2-SVPD(Cl) SMD			M06-2X/def2-TZVP(H,C,N,O,S,Ru)/def2-TZVPD(Cl)//B3LYP/def2-SVP(H,C,N,O,S,Ru)/def2-SVPD(Cl)		
**Isomer**	**E (a.u)**	**G (a.u)**	**ΔG (kcal mol^{-1})**	**E (a.u)**	**G (a.u)**	**ΔG (kcal mol^{-1})**	**E (a.u)**	**G (a.u)**	**ΔG (kcal mol^{-1})**
cis,mer-**2dd**	-2852.825857	-2852.369590	7.8	-2852.079630	-2851.623363	8.5	-2851.796328	-2851.340061	8.6
cis,mer-**2du**	-2852.830647	-2852.373777	5.2	-2852.084273	-2851.627403	6.0	-2851.801559	-2851.344689	5.7
trans,mer-**2a**	-2852.830721	-2852.374522	4.7	-2852.085198	-2851.628999	5.0	-2851.803383	-2851.347184	4.1
trans,fac-**2b**	-2852.834966	-2852.378882	2.0	-2852.089135	-2851.633051	2.4	-2851.807189	-2851.351105	1.6
cis,fac-**2c-S**	-2852.837285	-2852.381416	0.4	-2852.092202	-2851.636333	0.4	-2851.809507	-2851.353638	0.0
cis,fac-**2c-Cl**	-2852.836969	-2852.381999	0.0	-2852.091872	-2851.636901	0.0	-2851.808666	-2851.353696	0.0

Scheme S1. Possible isomers for [$Ru^{II}Cl_2$(bpea-pyrene)(dmso)].

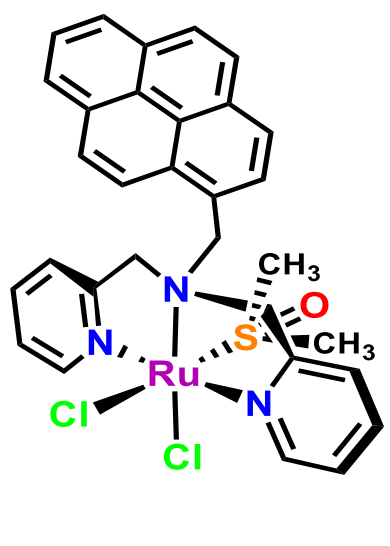

cis,mer-**2dd**

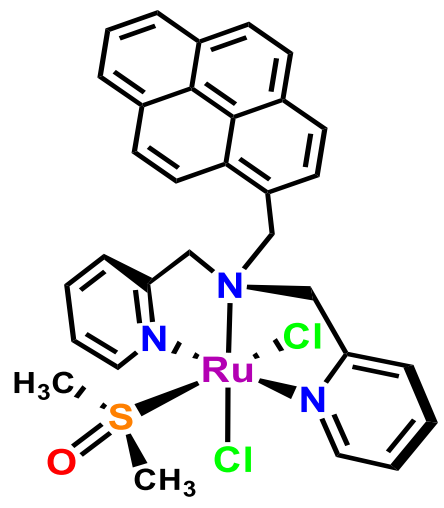

cis,mer-**2du**

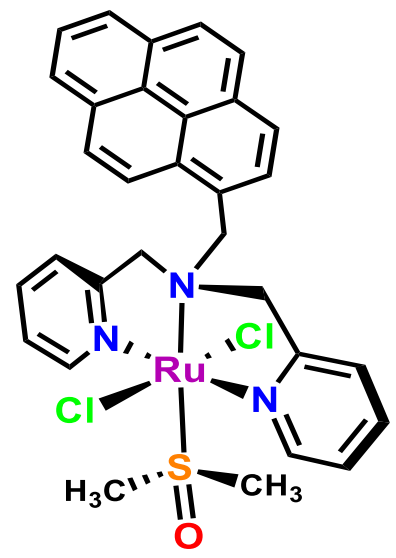

trans,mer-**2a**

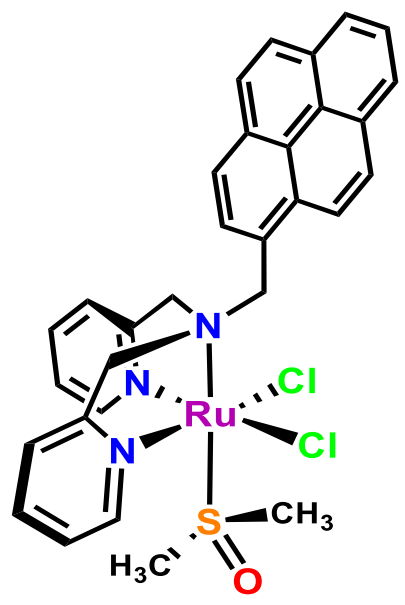

trans,fac-**2b**

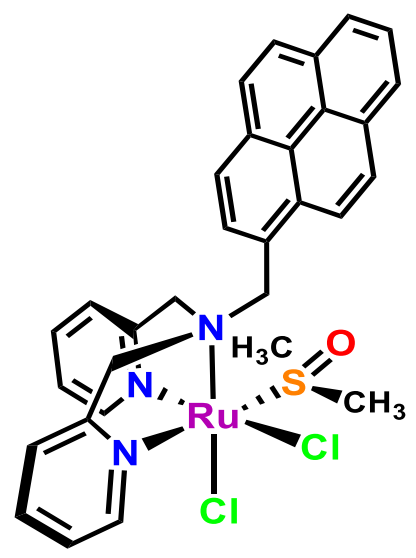

cis,fac-**2c**

Figure S1. A) Molecular structures of the enantiomers in the isomer **2c**; b) Hydrogen bond interactions for **2a** (1); **2b** (2) and **2c** (3). Circles in grey correspond to C atoms; in yellow to H atoms, in green to Cl atoms, in orange to S atoms, in red to O atoms, in blue to N atoms and, in greenish blue to Ru atom.

a)

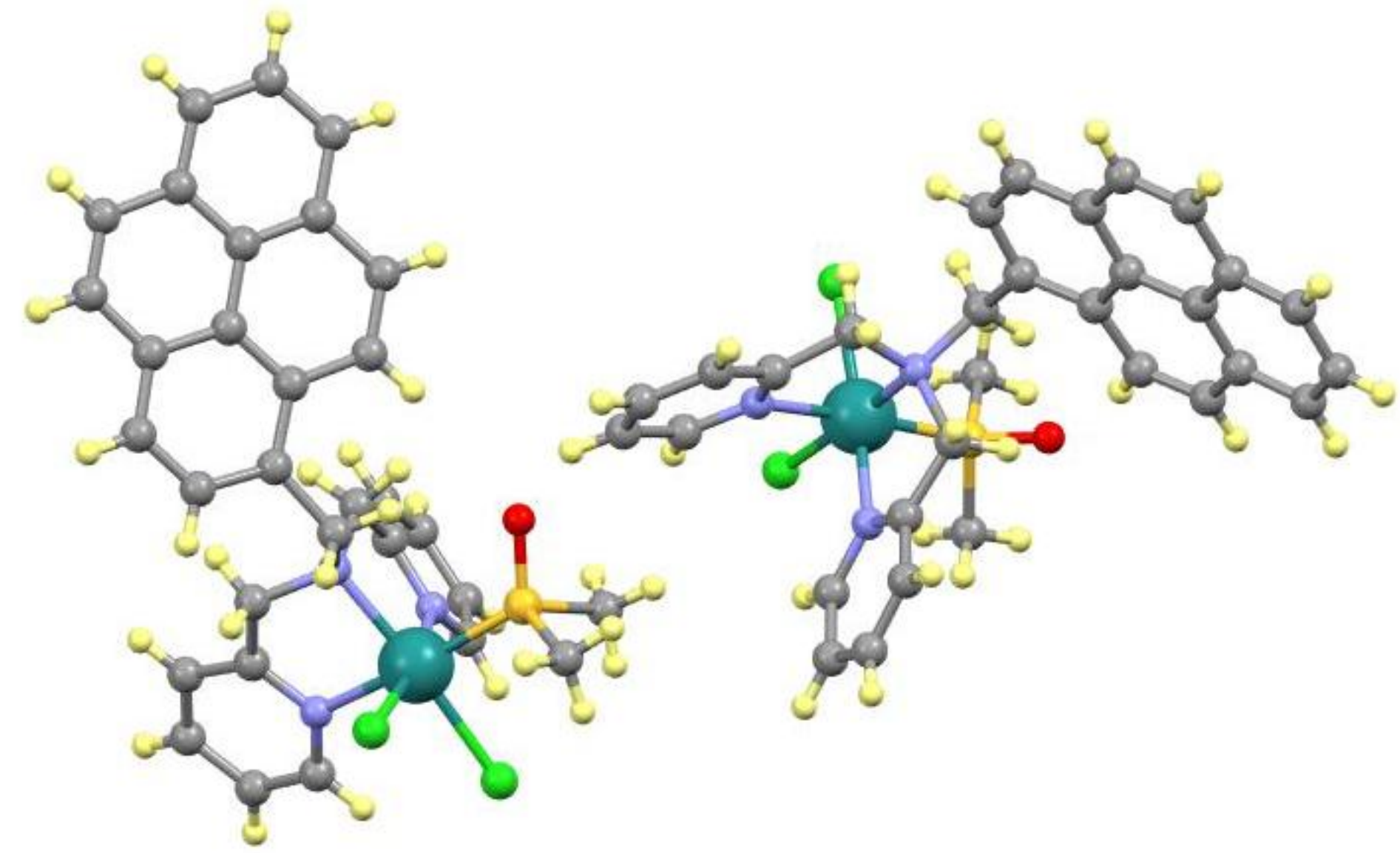

b)

1)

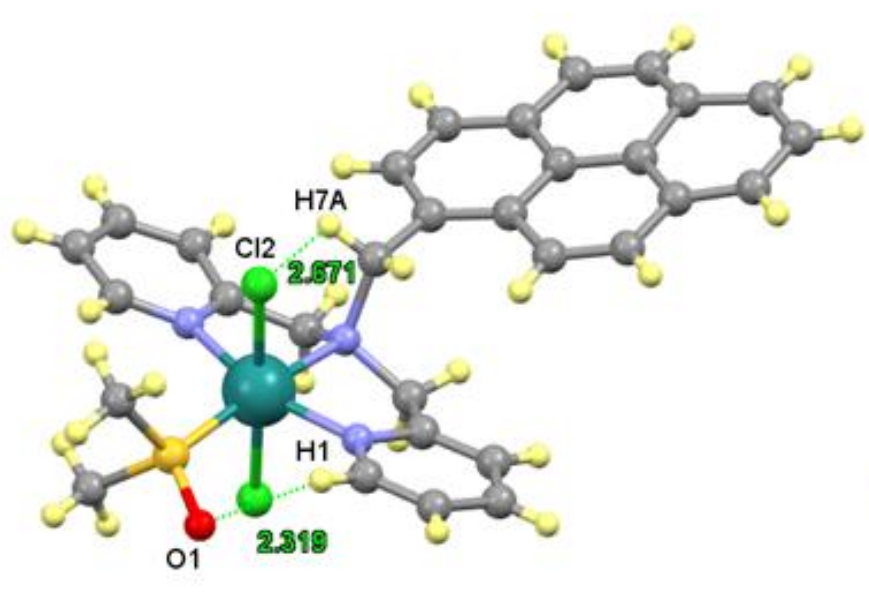

2)

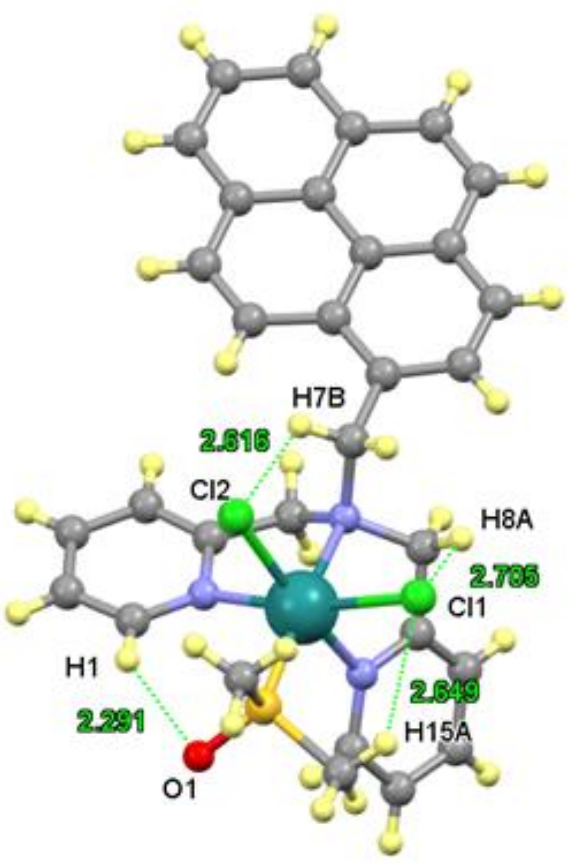

3)

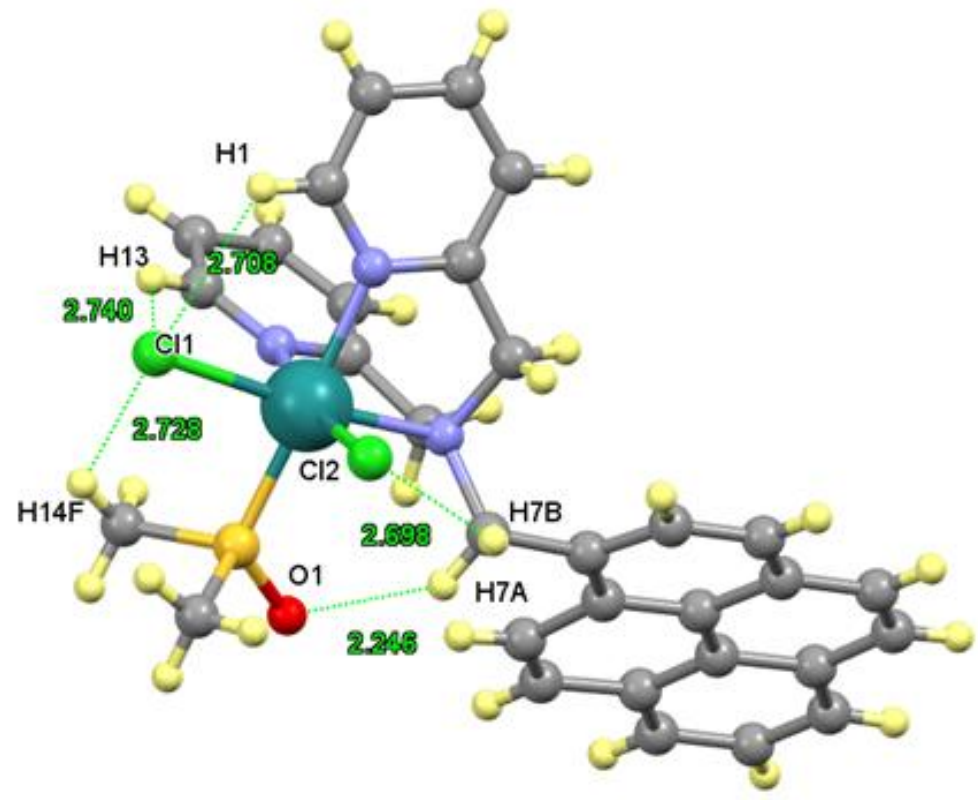

Figure S2. Packing arrangement for a) **2a** along axis a (1), b (2) and c (3); b) **2b** along axis a (4), b (5) and c (6) and c) **2c** along axis a (7) and c (8).

a)

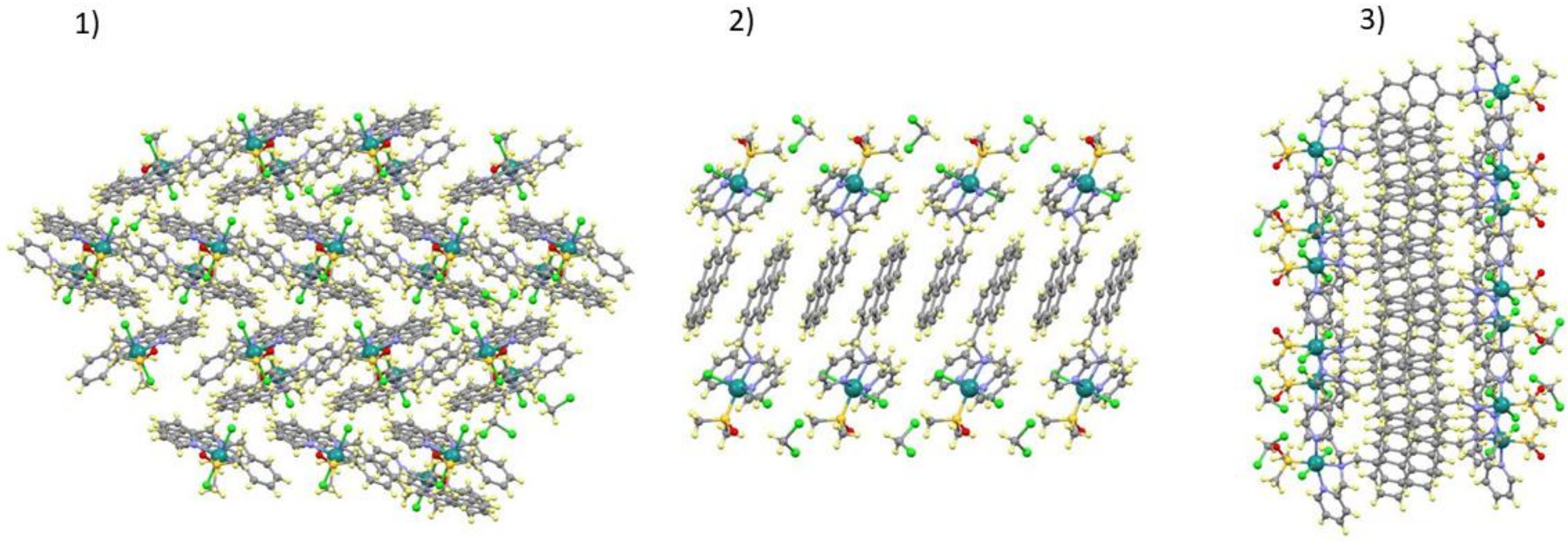

b)

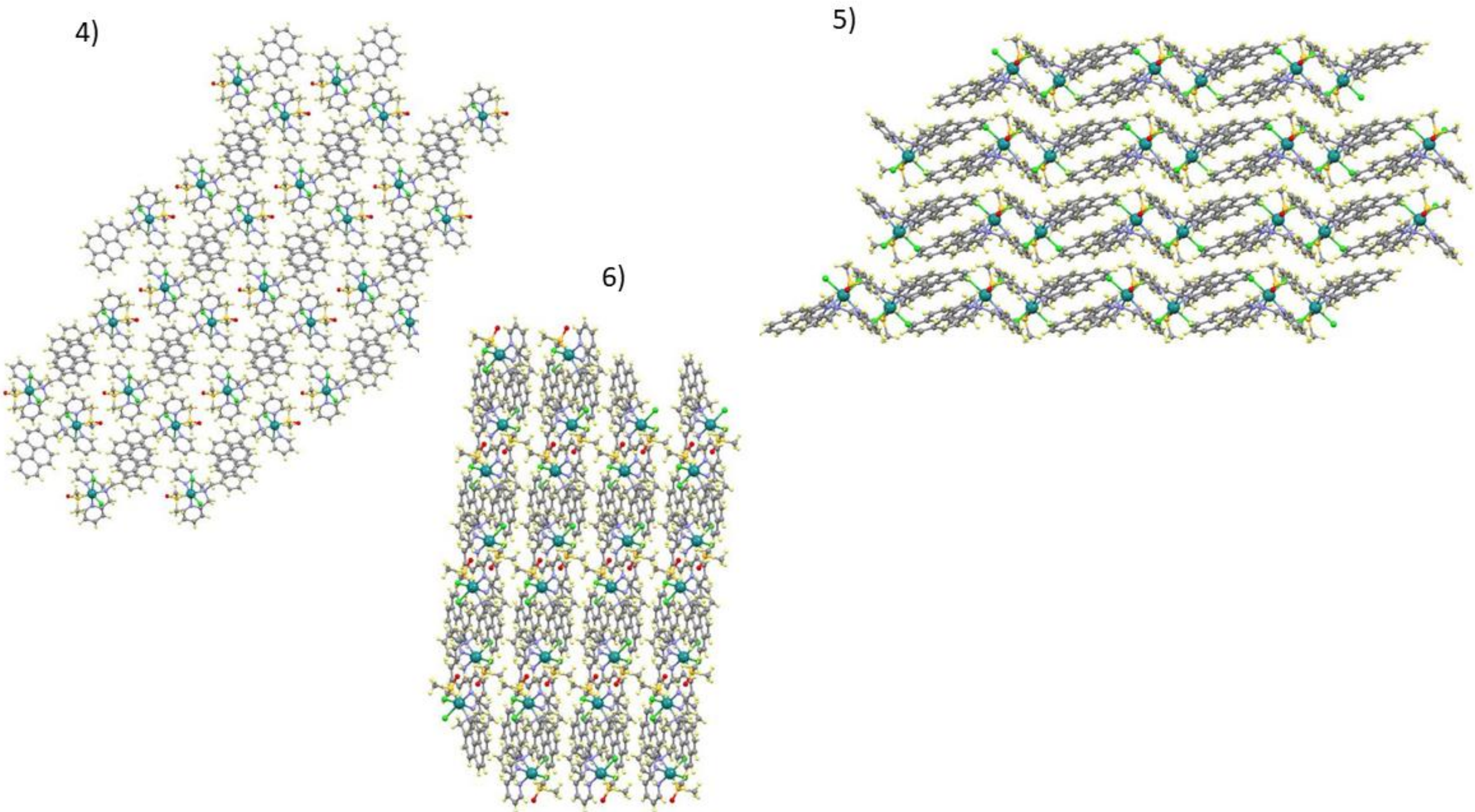

c)

7)

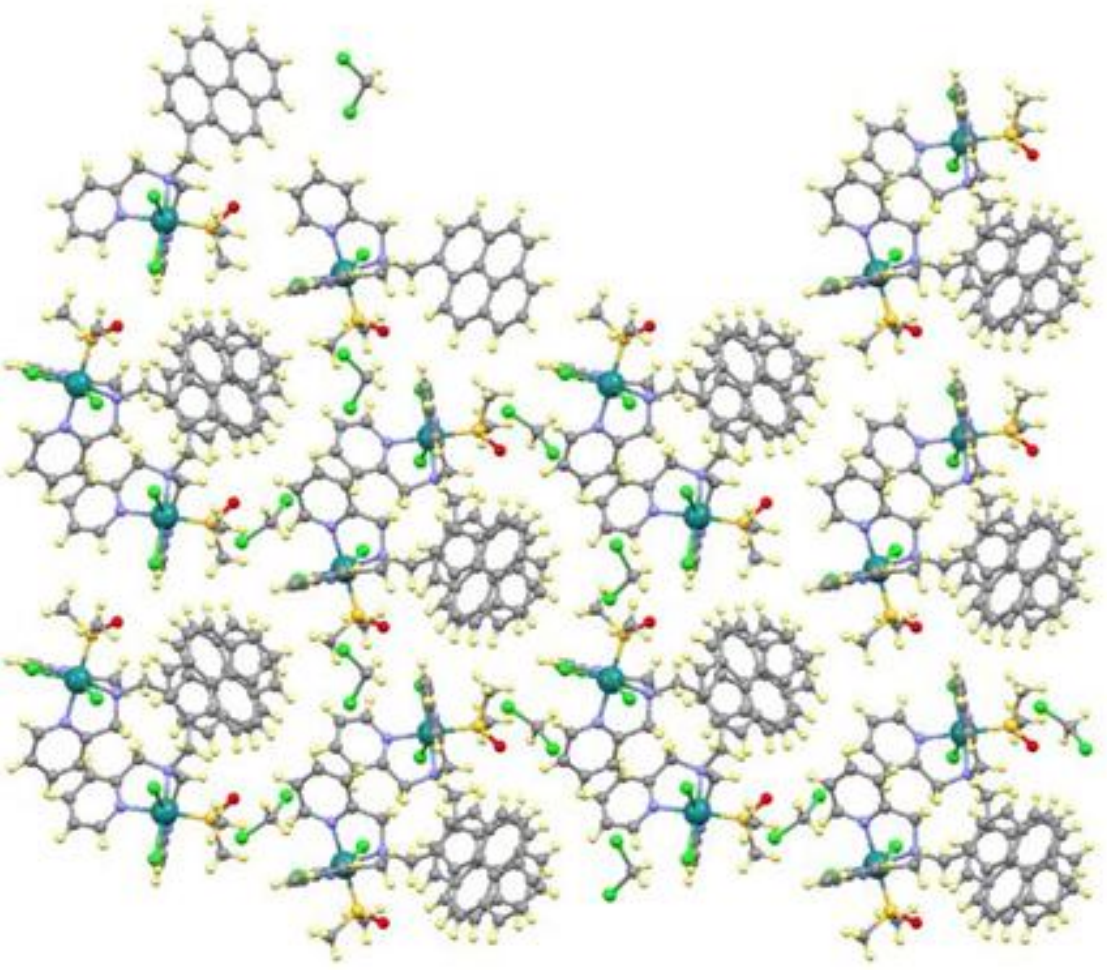

8)

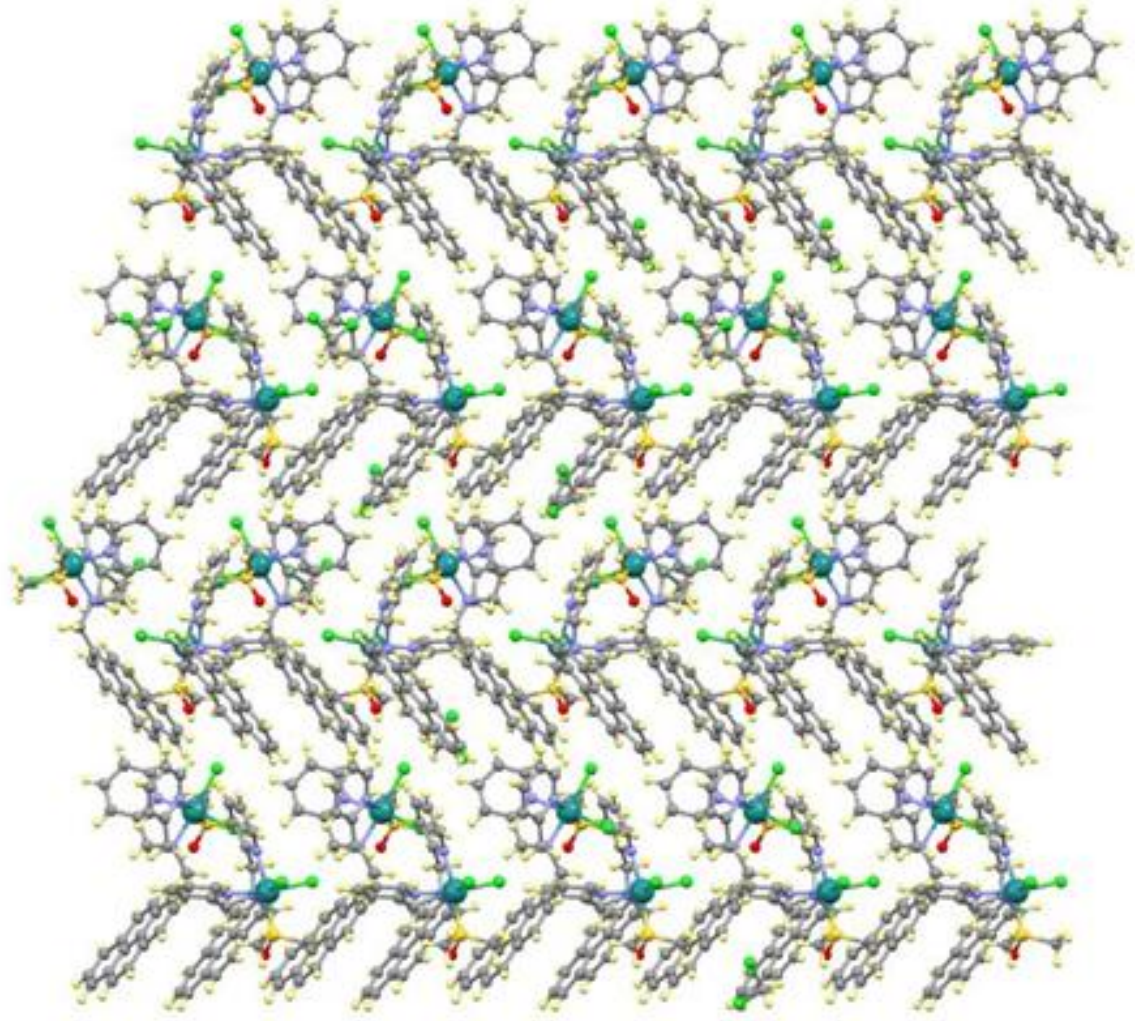

Figure S3. IR spectra of a) **2a** (blue); **2b** (red) and **2c** (green) and b) *cis,fac*-**5s**.

a)

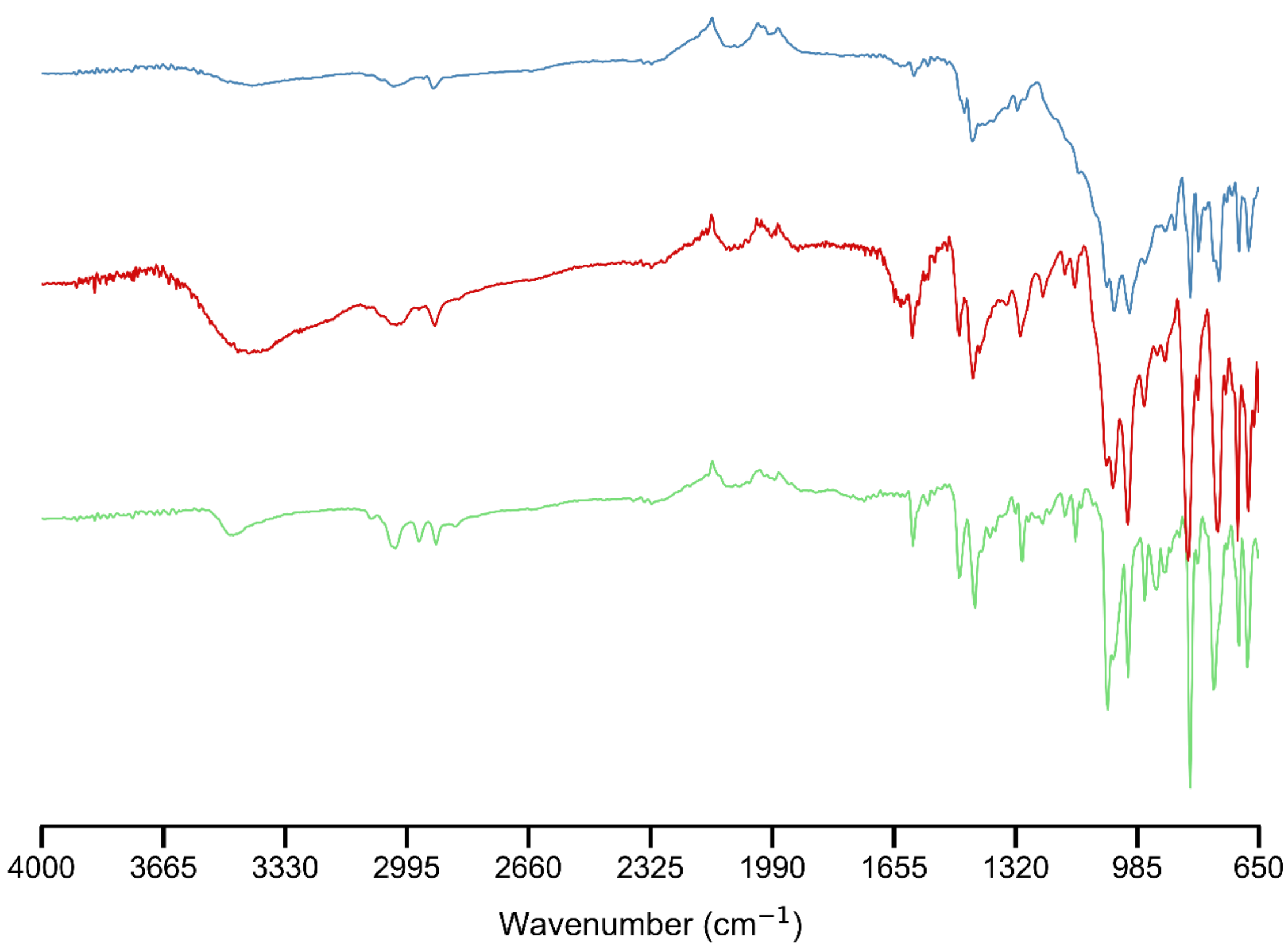

b)

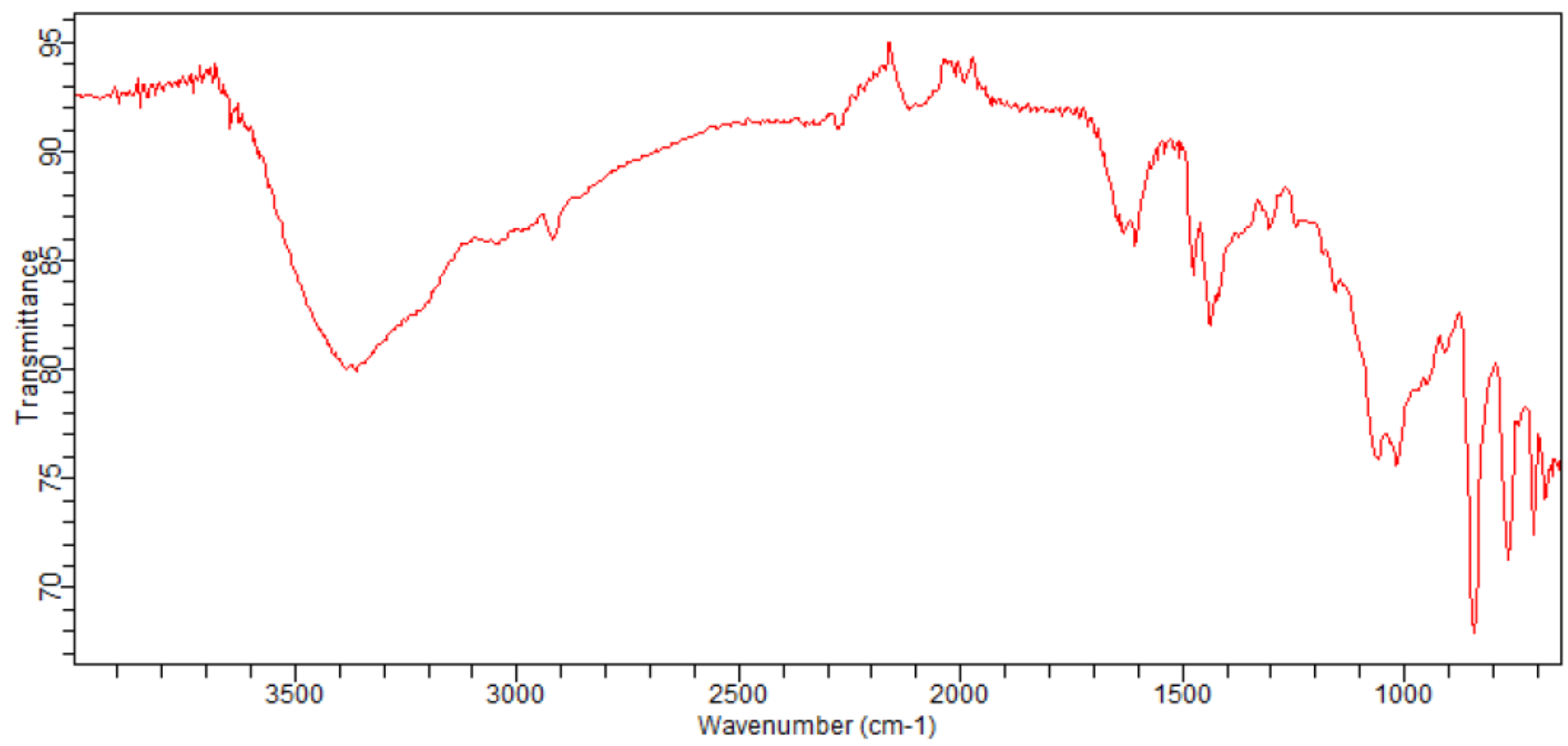

Figure S4. NMR spectra of **2a**, 400 MHz, CD_2Cl_2: a) ^{1}H-NMR; b) COSY; c) NOESY; d) ^{1}H-^{13}C HSQC.

a)

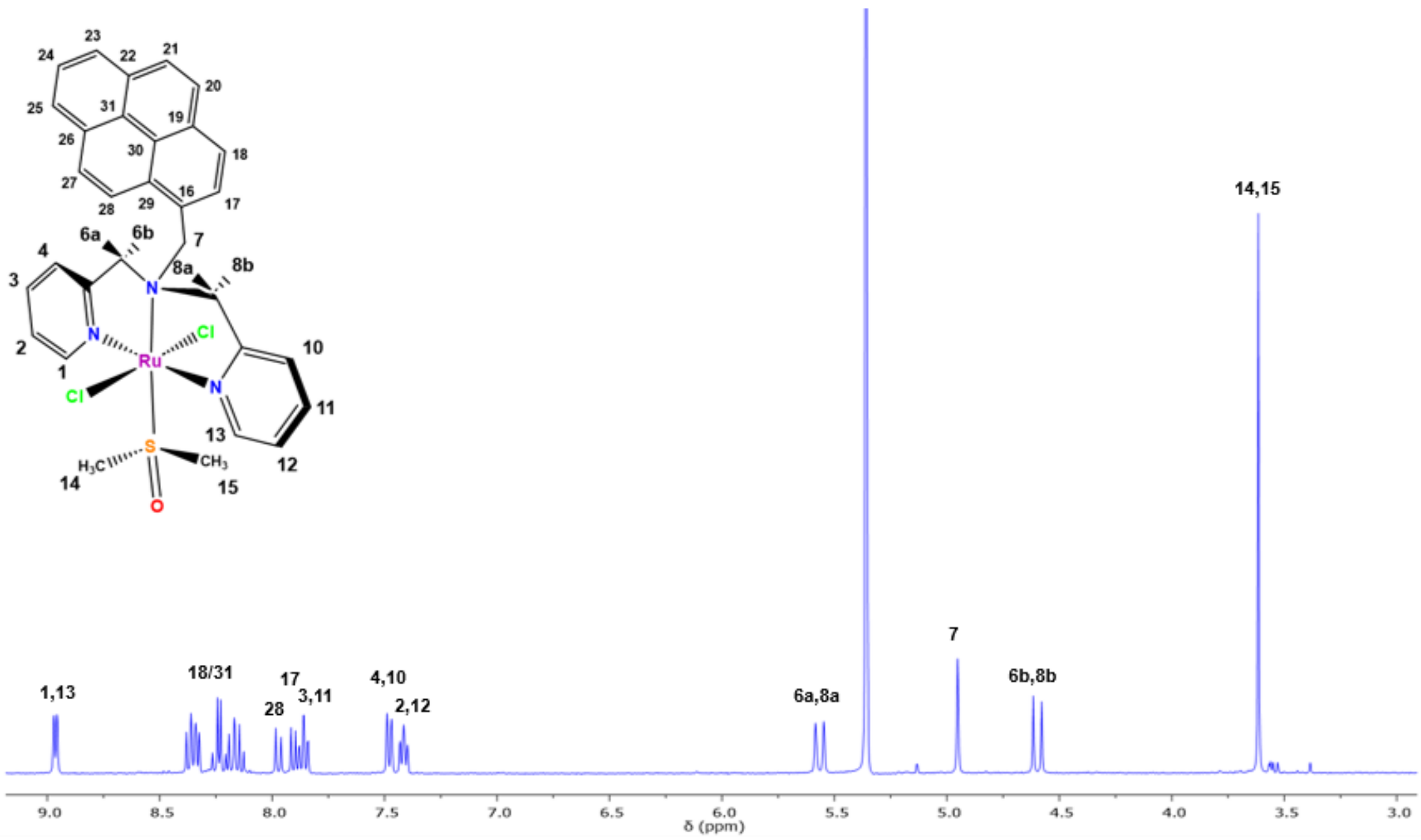

b)

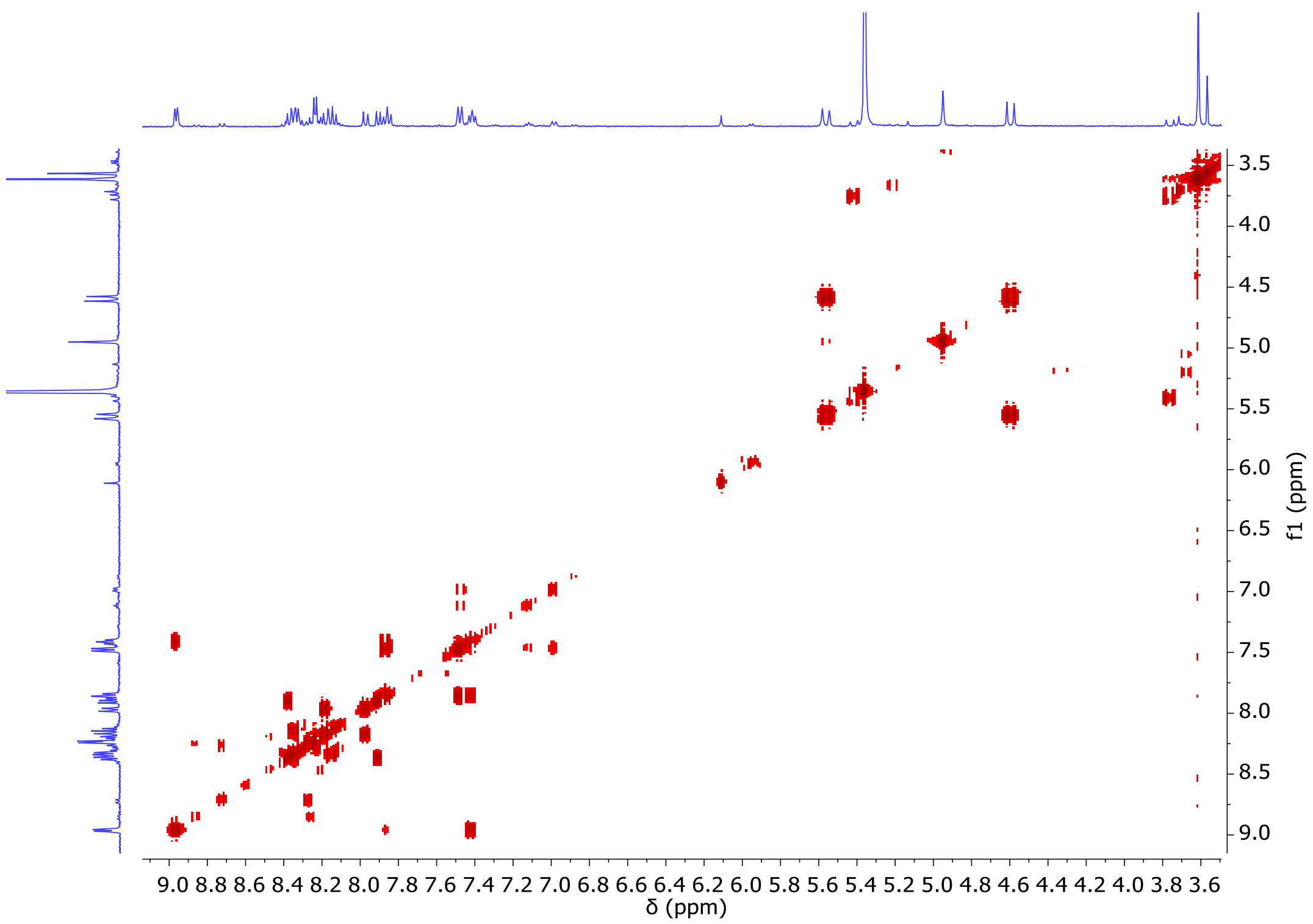

c)

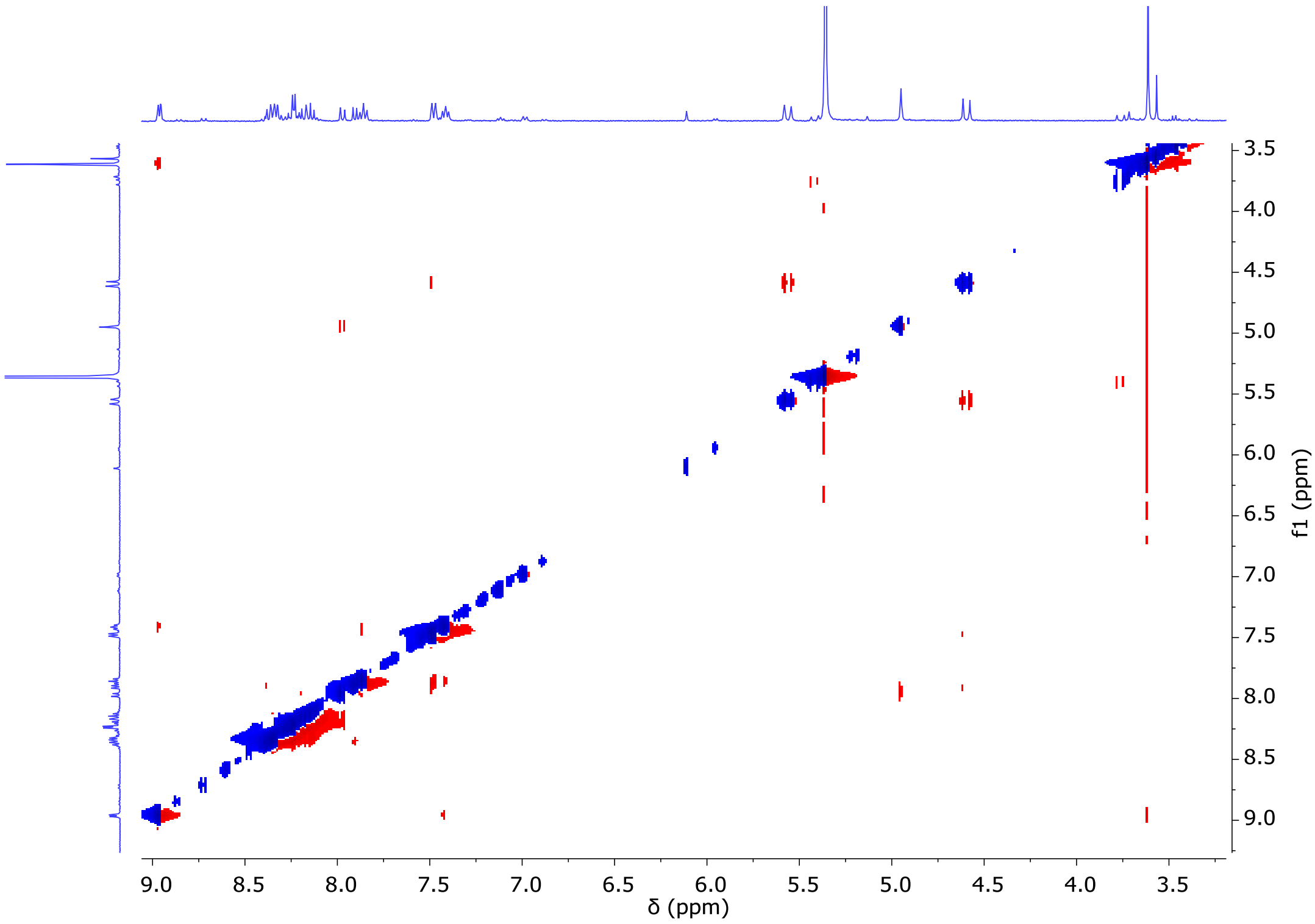
3.5
4.0
4.5
5.0
5.5
6.0
6.5
7.0
7.5
8.0
8.5
9.0
f1 (ppm)
9.0 8.5 8.0 7.5 7.0 6.5 6.0 5.5 5.0 4.5 4.0 3.5
δ (ppm)

d)

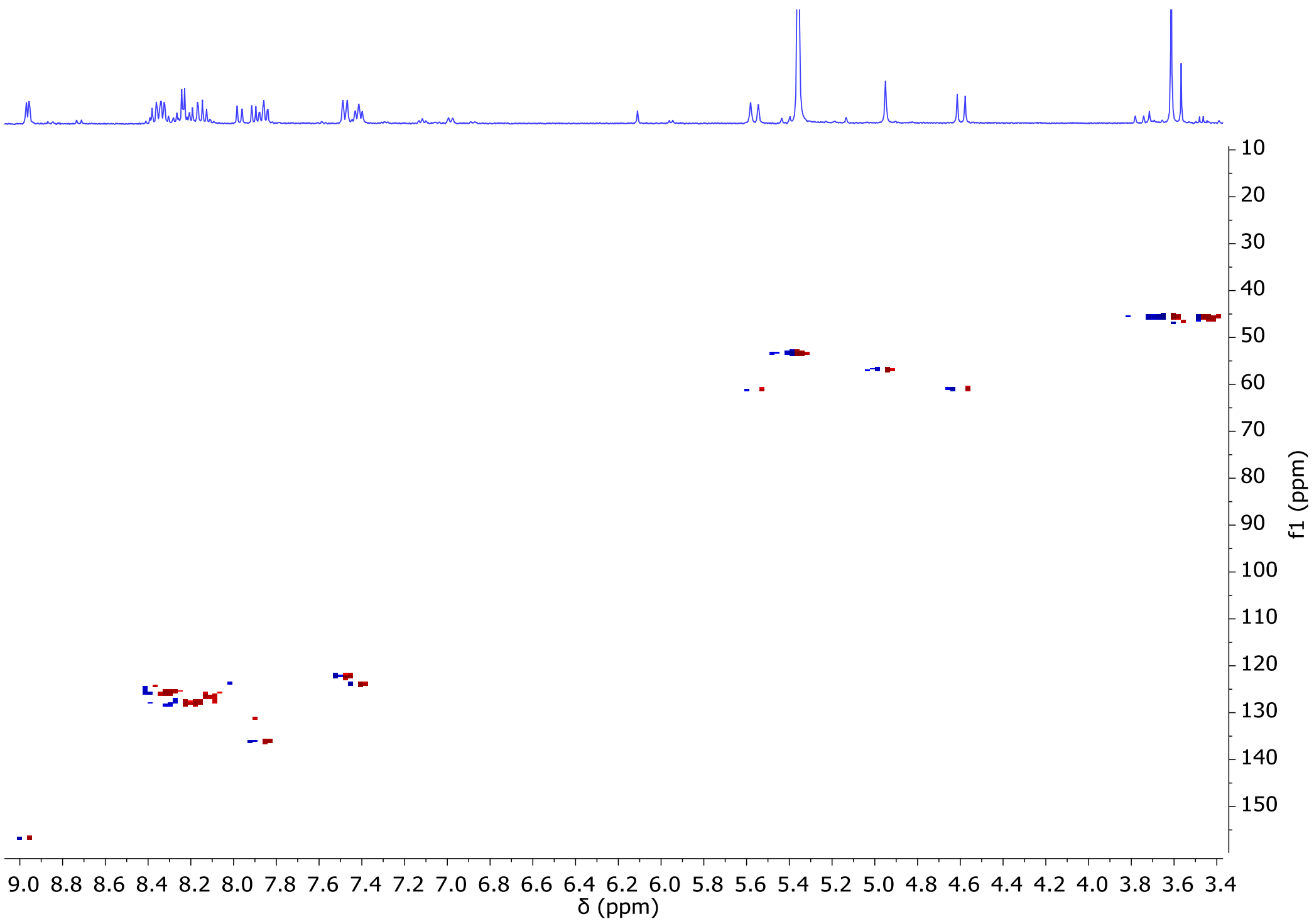
10
20
30
40
50
60
70
80
90
100
110
120
130
140
150
f1 (ppm)
9.0 8.8 8.6 8.4 8.2 8.0 7.8 7.6 7.4 7.2 7.0 6.8 6.6 6.4 6.2 6.0 5.8 5.6 5.4 5.2 5.0 4.8 4.6 4.4 4.2 4.0 3.8 3.6 3.4
δ (ppm)

Figure S5. NMR spectra of **2b**, 400 MHz, CD_2Cl_2: a) ^{1}H-NMR; b) ^{13}C-NMR; c) COSY; d)^{1}H-^{13}C HSQC.

a)

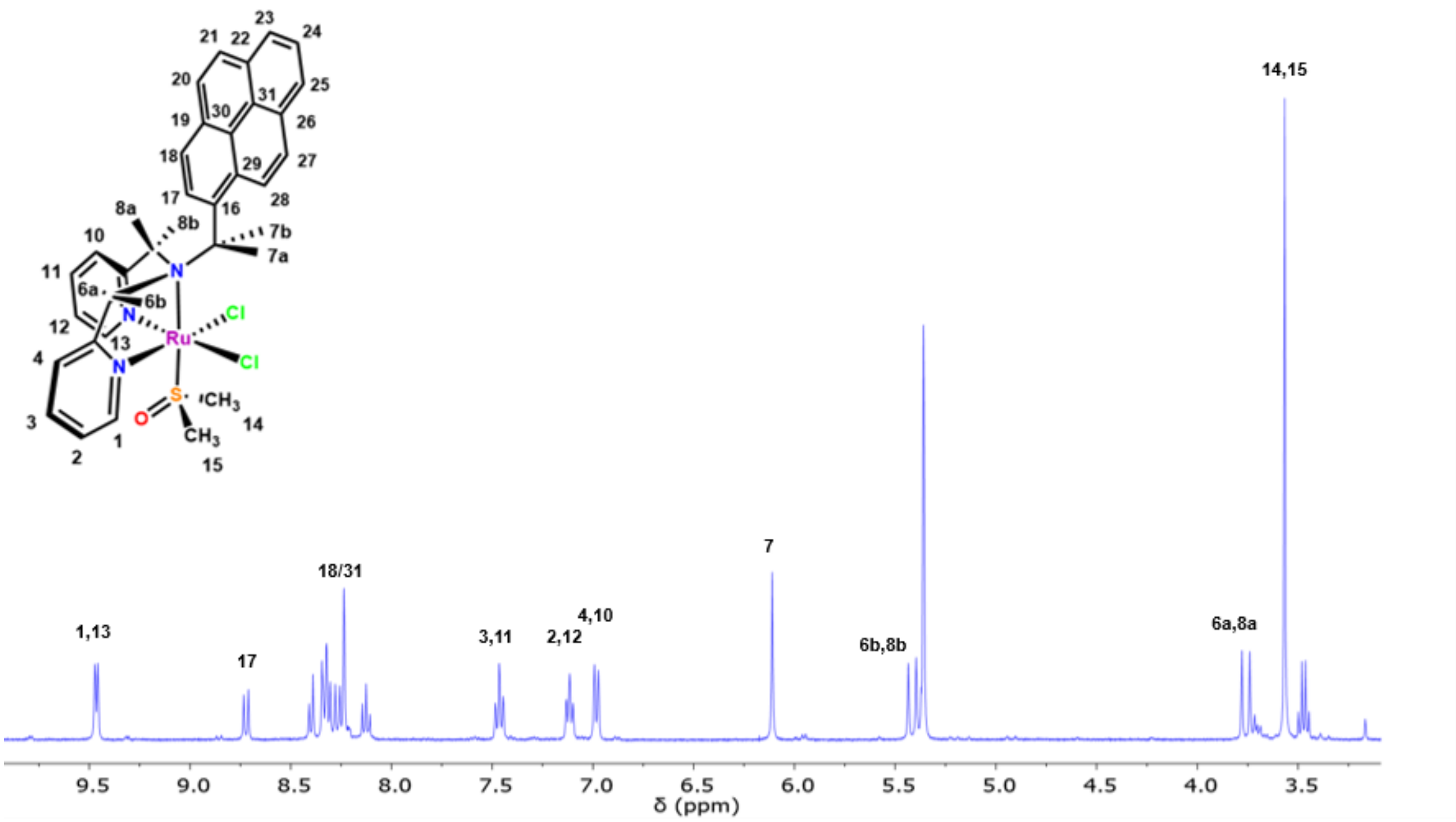

b)

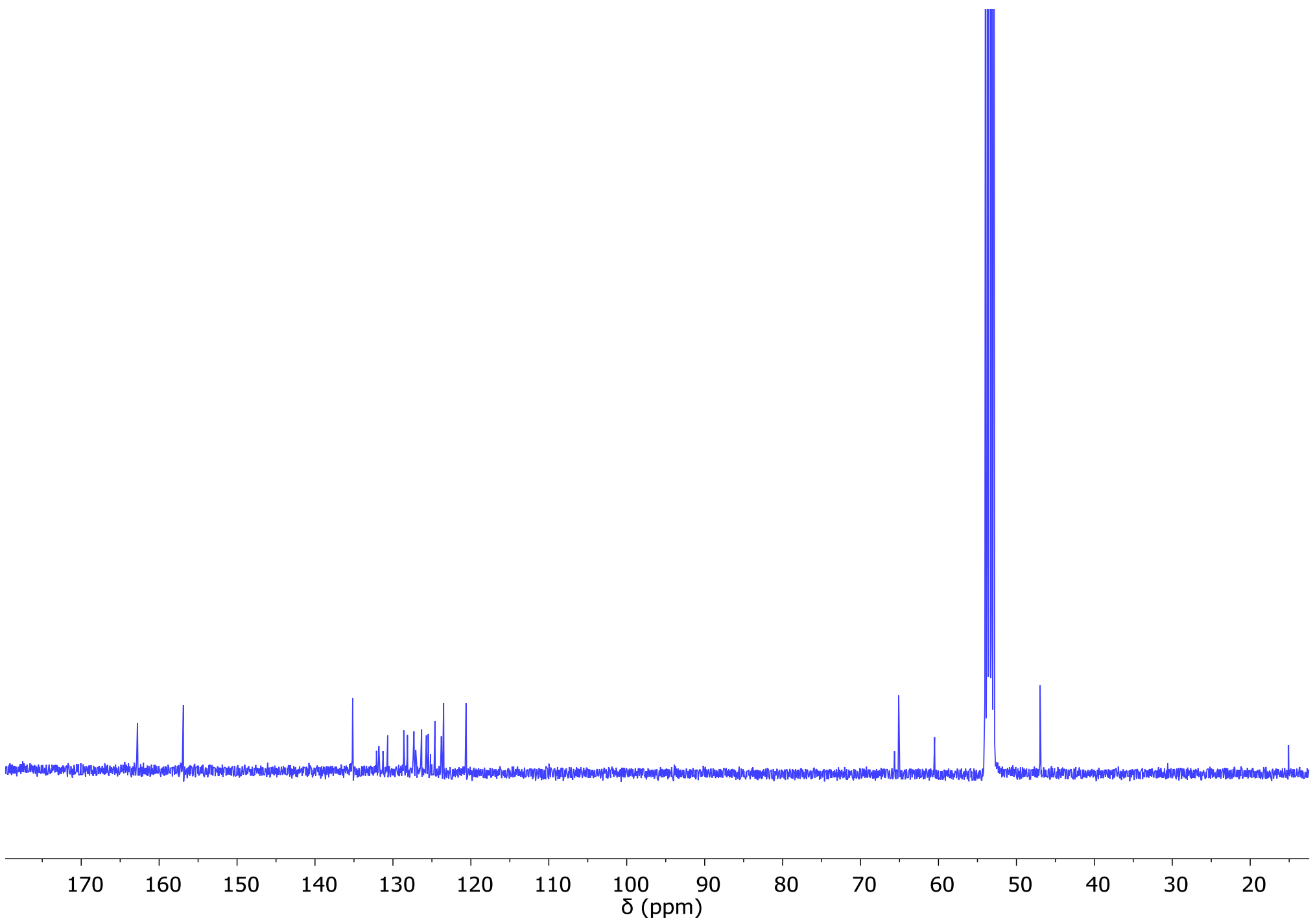

c)

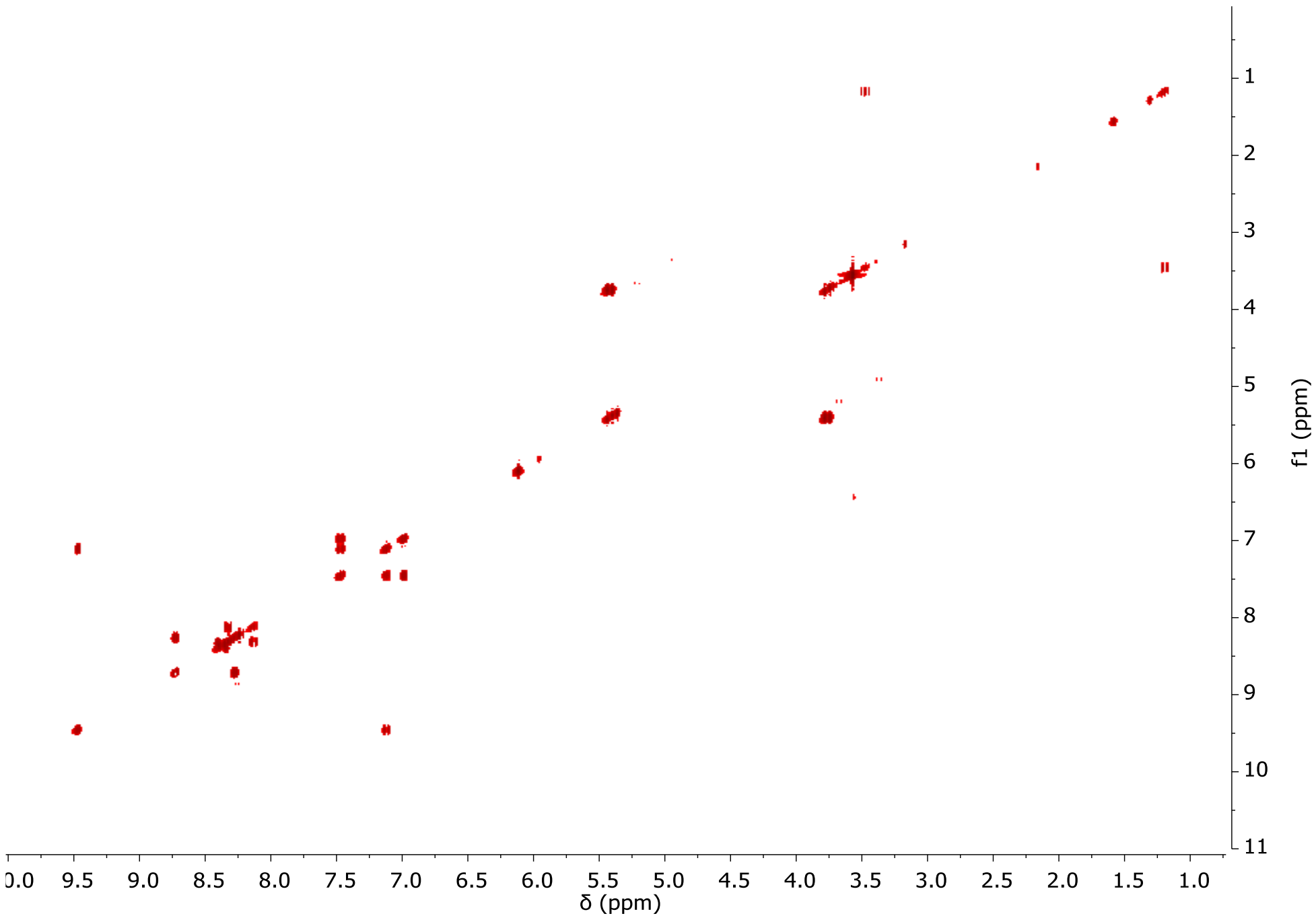

d)

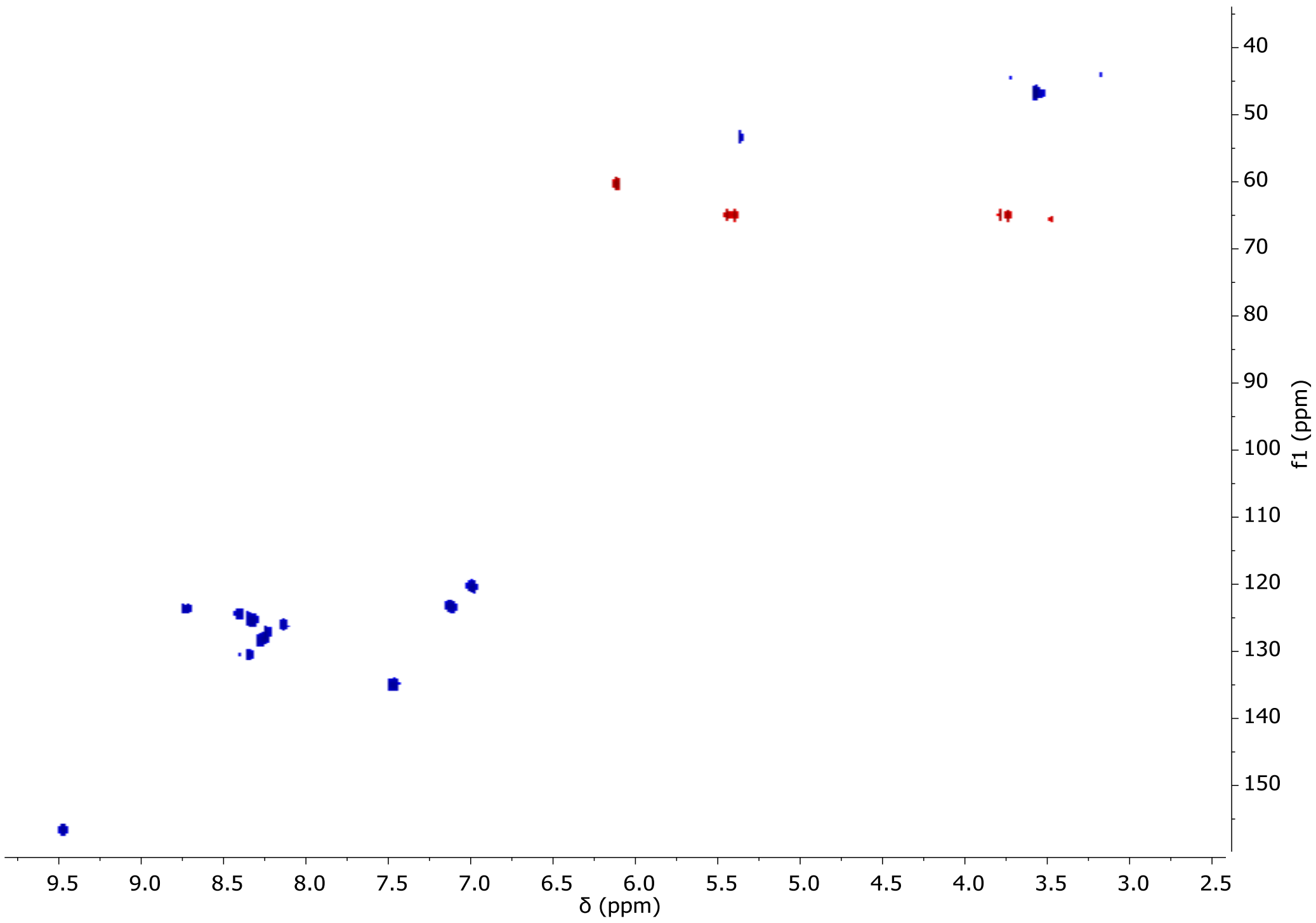

Figure S6. NMR spectra of **2c**, 400 MHz, CD_2Cl_2: a) ^{1}H-NMR; b) ^{13}C-NMR; c) COSY; d) NOESY; e) ^{1}H-^{13}C HSQC; f) ^{1}H-^{13}C HMBC; g) NOESY-1D.

a)

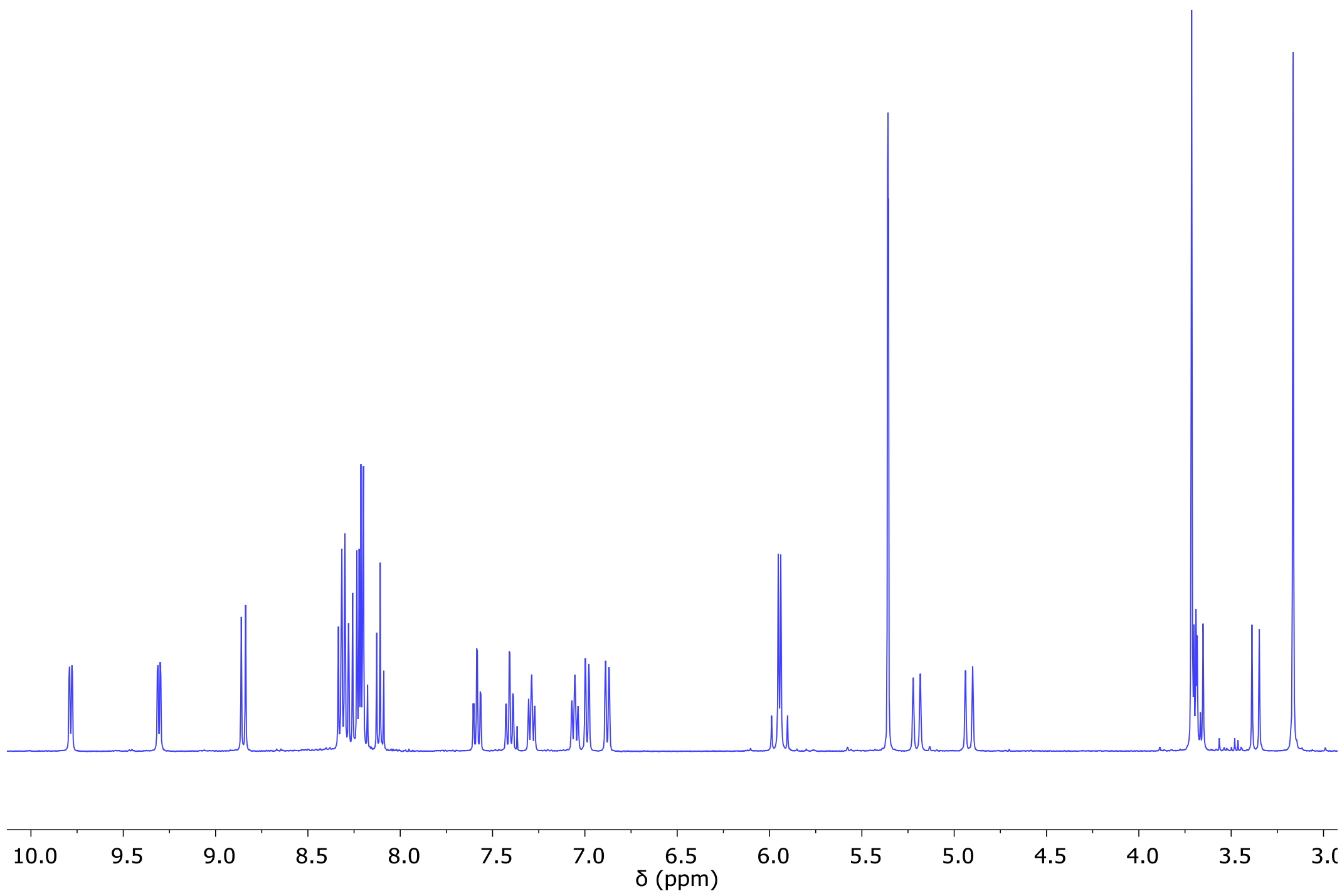

b)

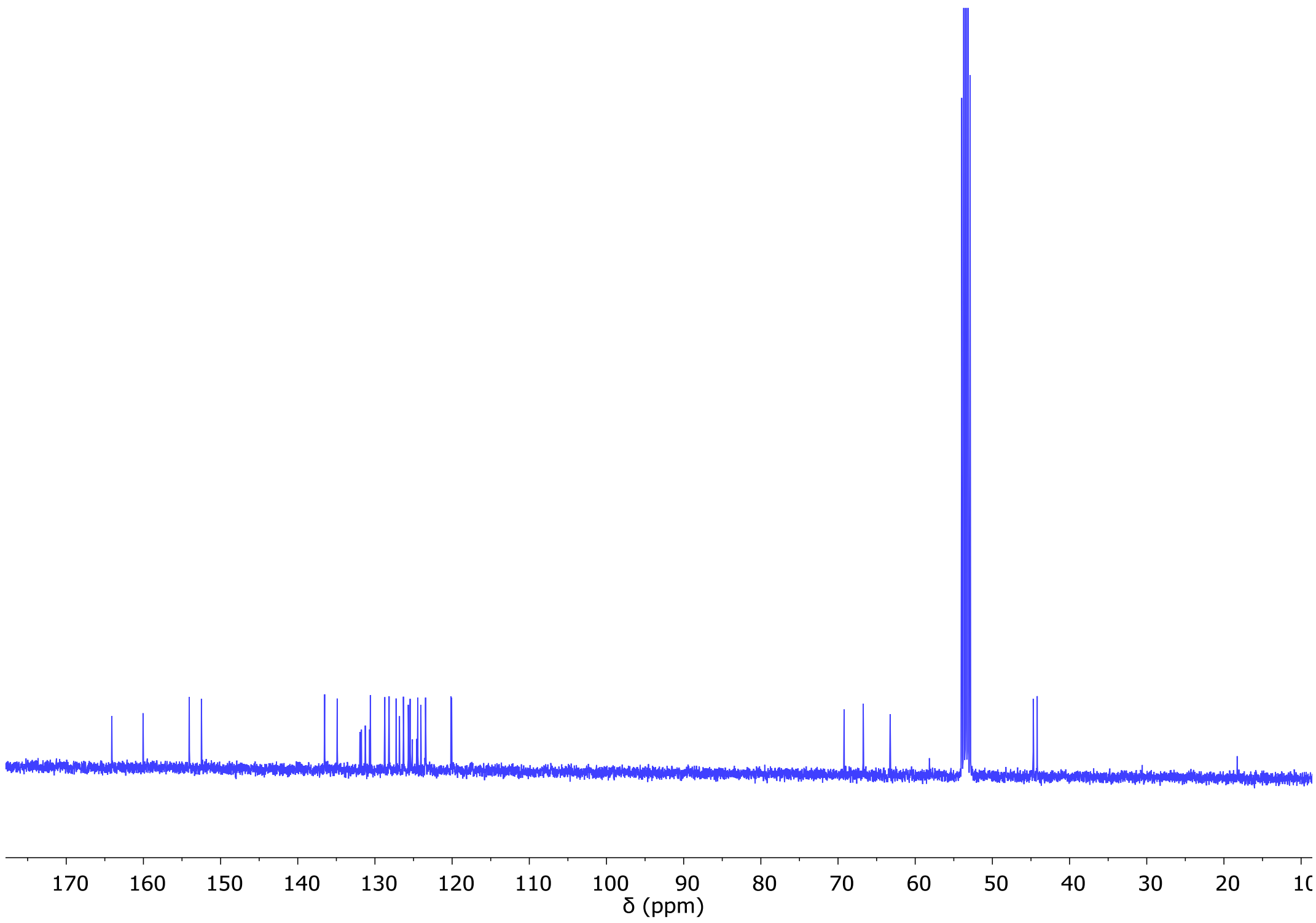

c)

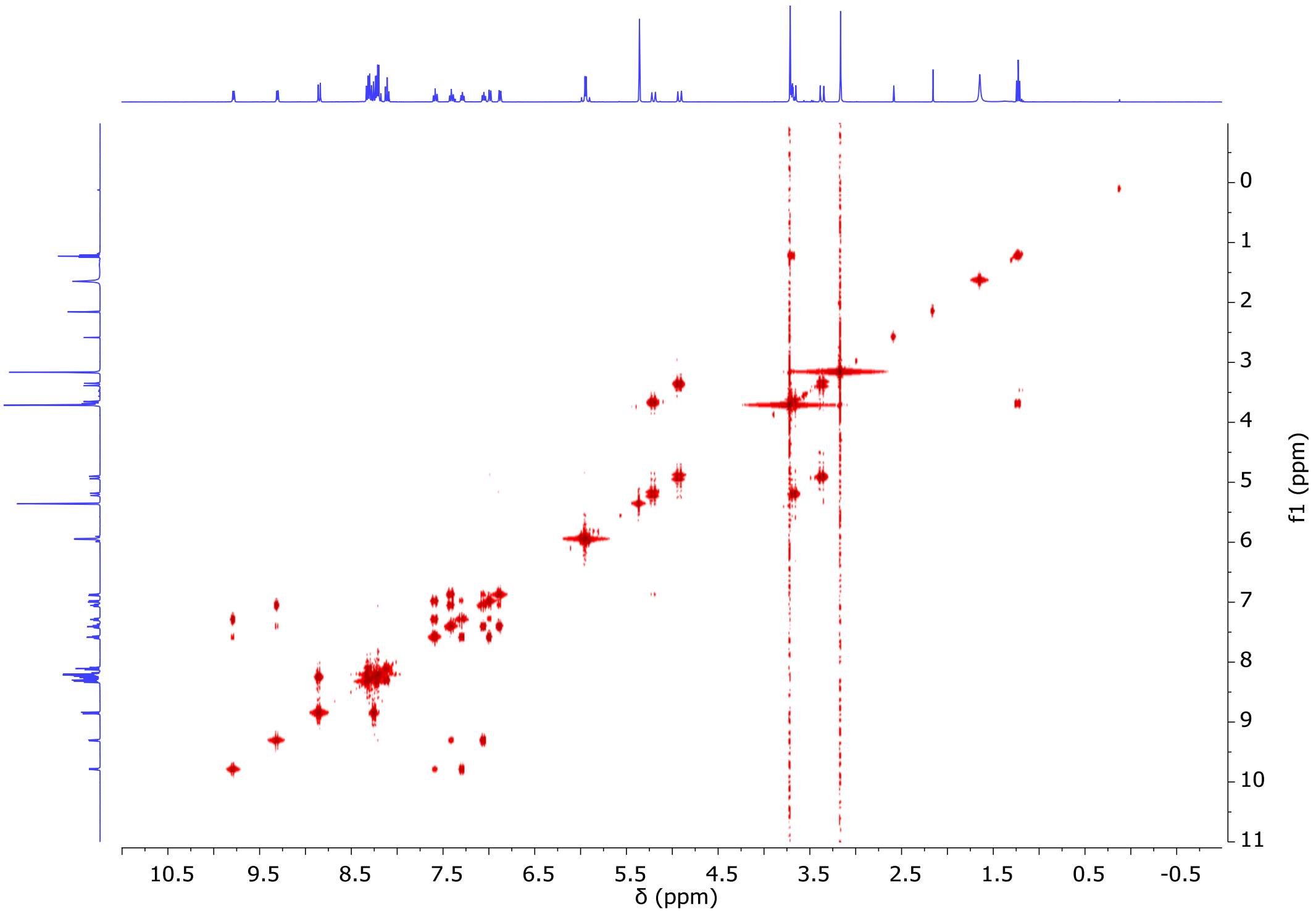

10.5
9.5
8.5
7.5
6.5
5.5
4.5
3.5
2.5
1.5
0.5
-0.5
δ (ppm)
0
1
2
3
4
5
6
7
8
9
10
11
f1 (ppm)

d)

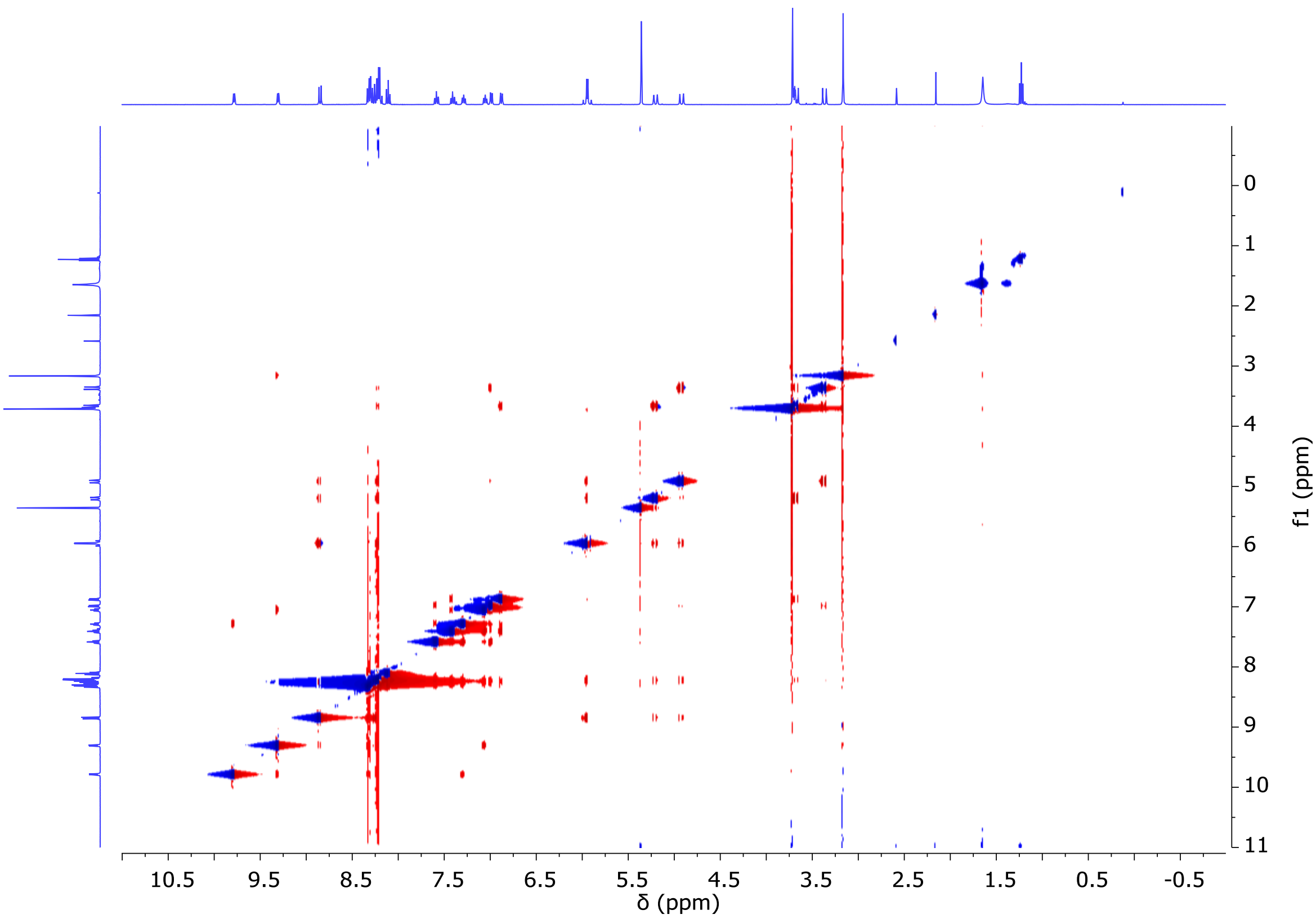

10.5
9.5
8.5
7.5
6.5
5.5
4.5
3.5
2.5
1.5
0.5
-0.5
δ (ppm)
0
1
2
3
4
5
6
7
8
9
10
11
f1 (ppm)

e)

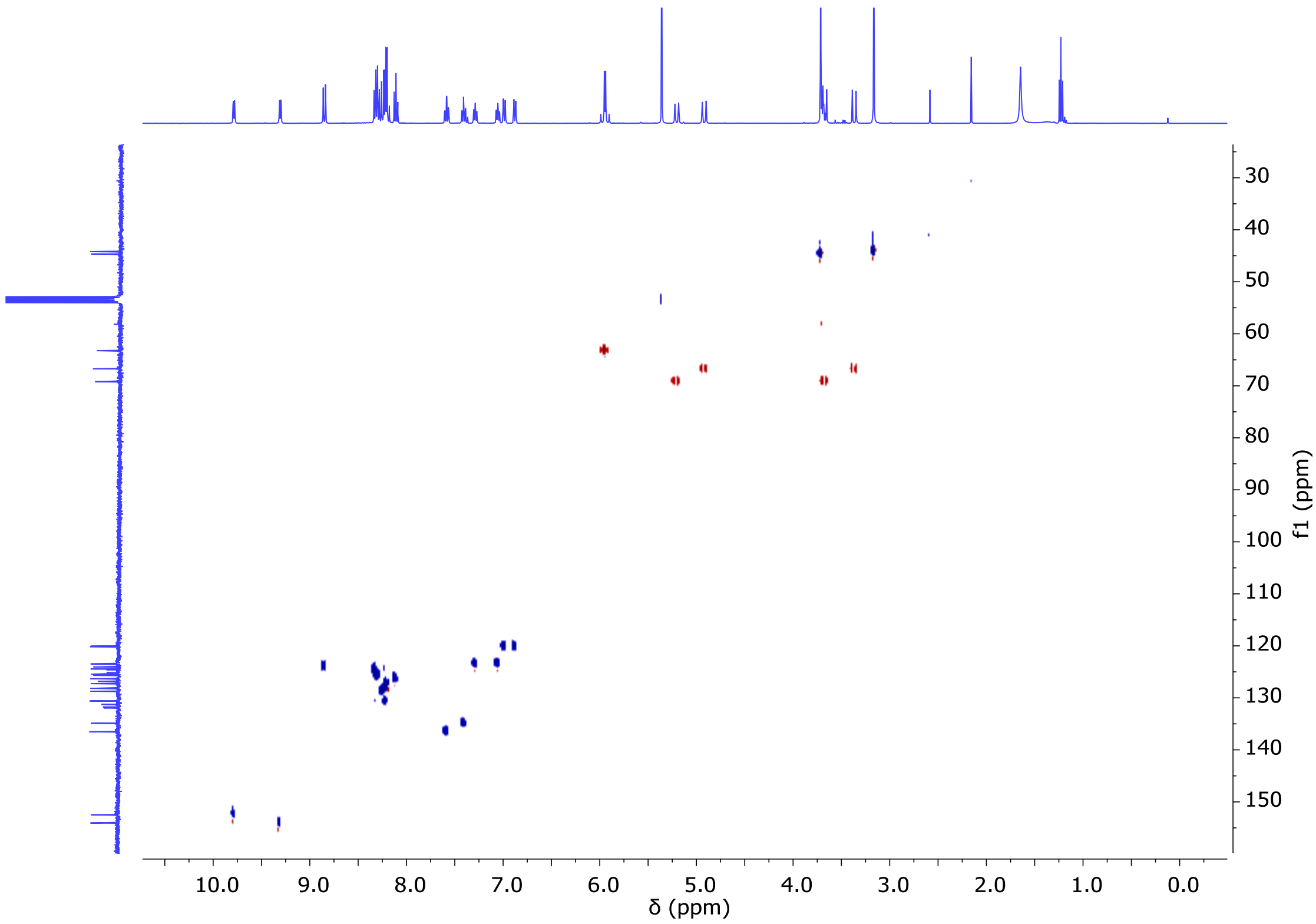

30
40
50
60
70
80
90
100
110
120
130
140
150
f1 (ppm)
10.0
9.0
8.0
7.0
6.0
5.0
4.0
3.0
2.0
1.0
0.0
δ (ppm)

f)

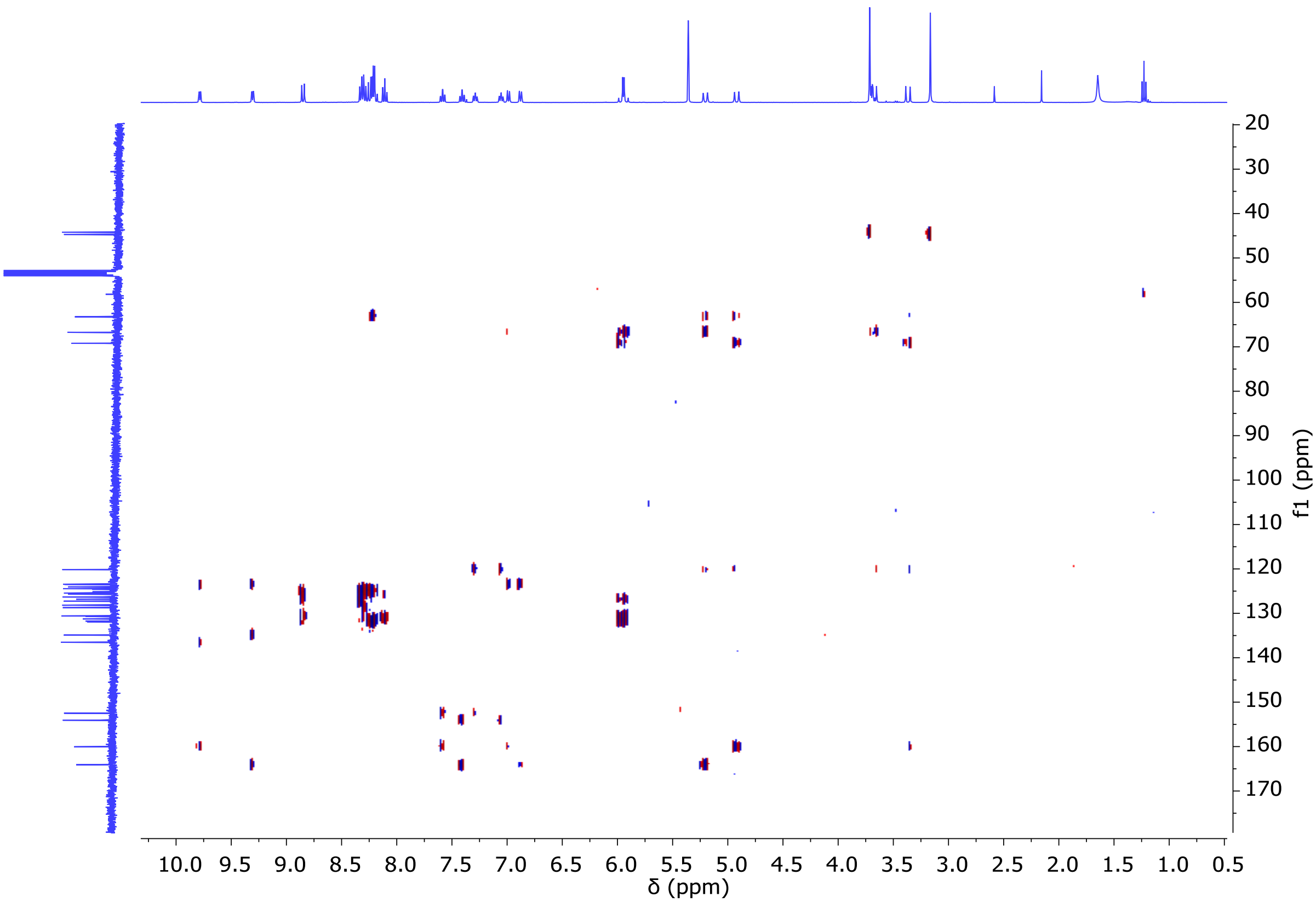

20
30
40
50
60
70
80
90
100
110
120
130
140
150
160
170
f1 (ppm)
10.0
9.5
9.0
8.5
8.0
7.5
7.0
6.5
6.0
5.5
5.0
4.5
4.0
3.5
3.0
2.5
2.0
1.5
1.0
0.5
δ (ppm)

Figure S7. A) UV-vis spectra of **2a** (0.1 Mm, blue line), **2b** (0.05 Mm red line) and **2c** (0.05 Mm green line) in MeOH. B) UV-vis spectrum of *fac*-**5s** (0.05 Mm). C) UV-Vis spectrum of bpea-pyrene ligand in MeOH (0.05 Mm).

a)

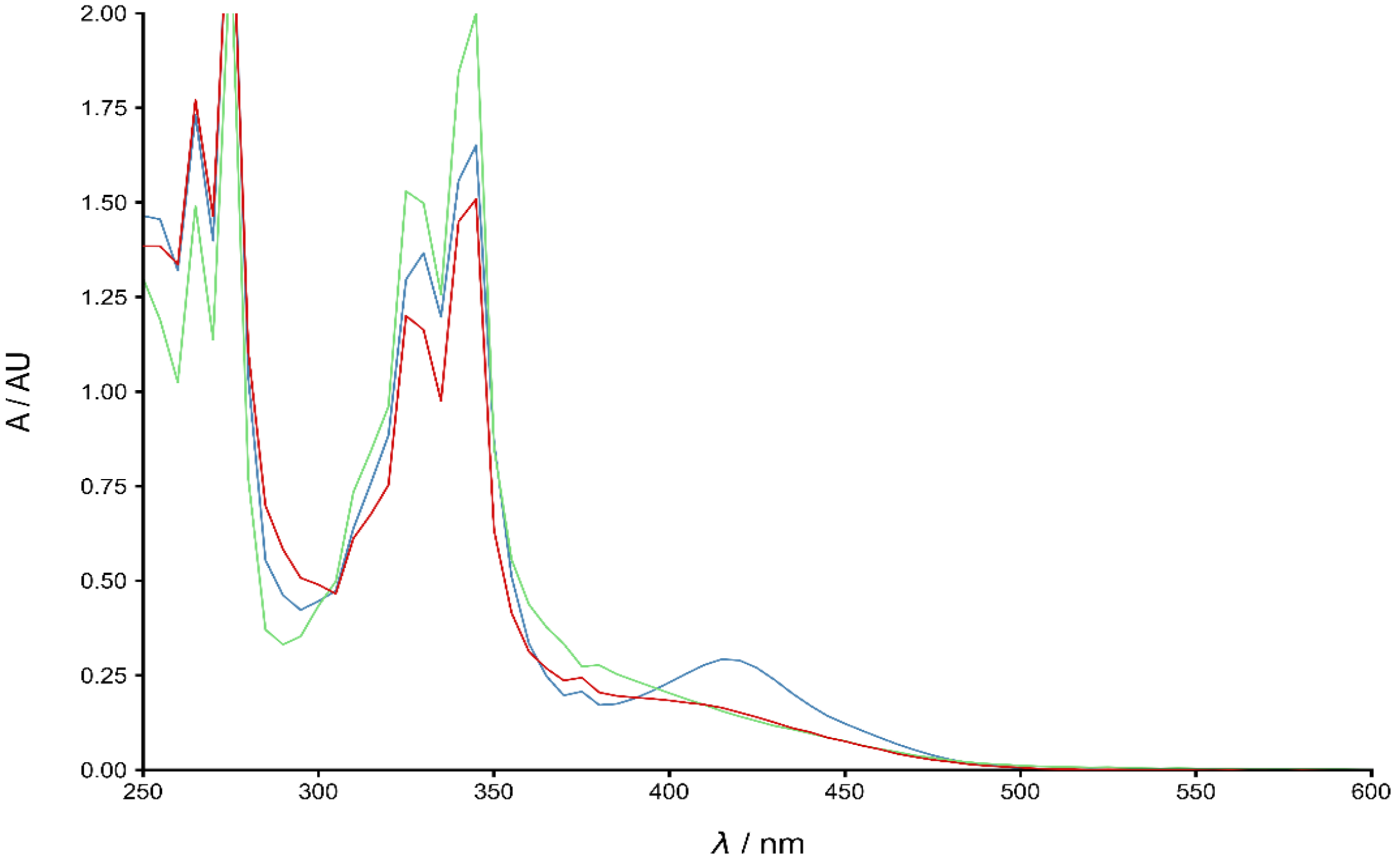

b)

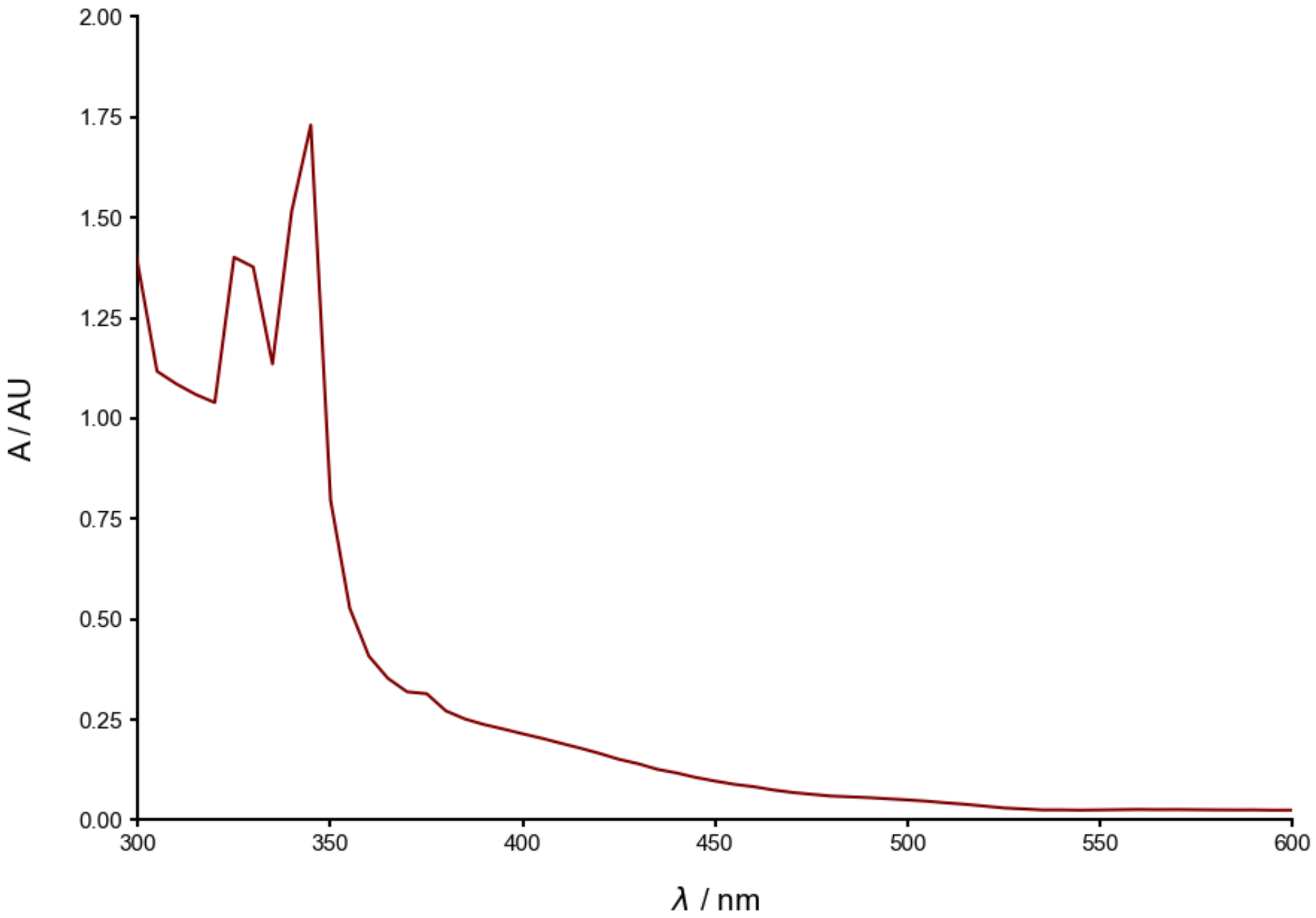

c)

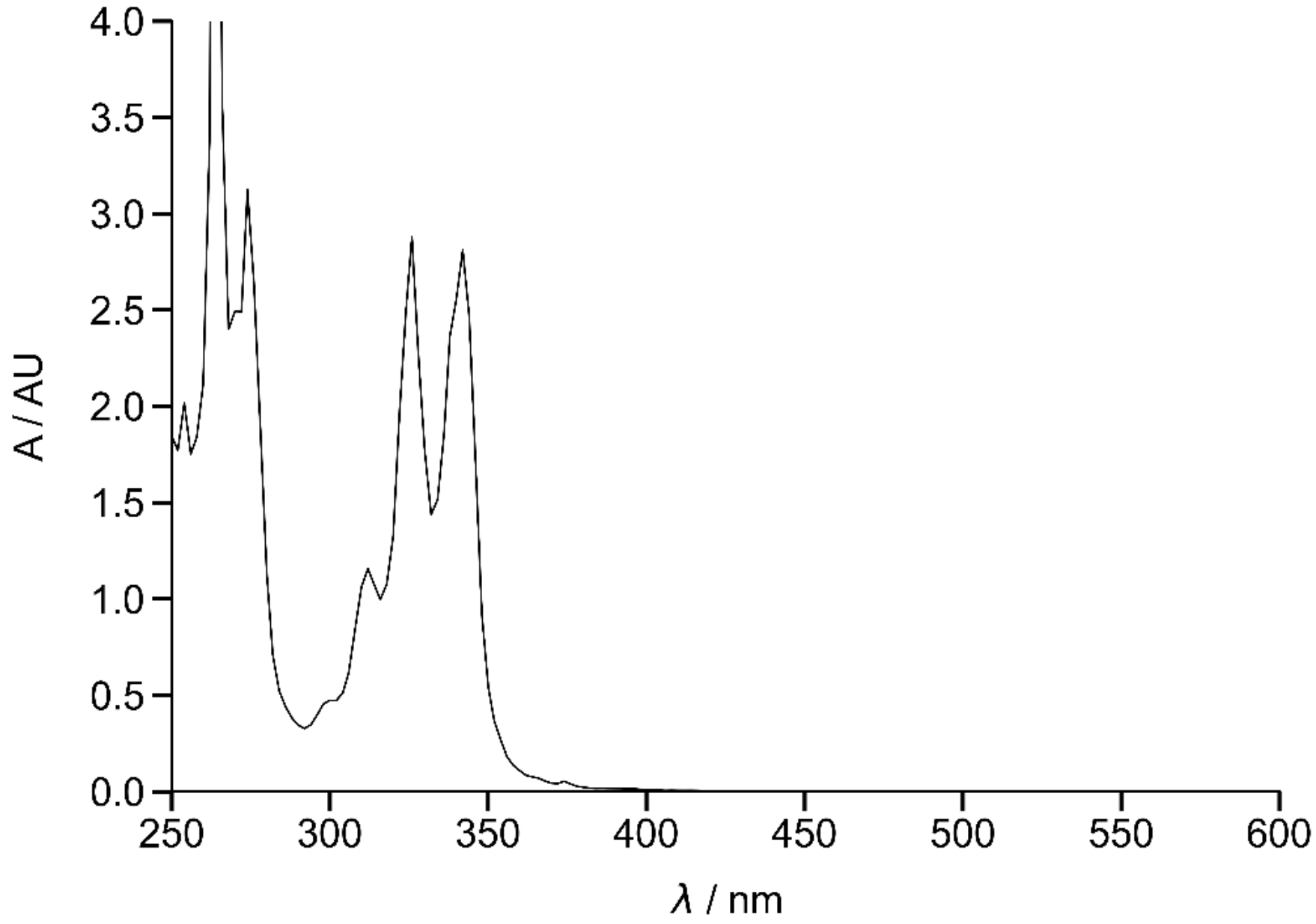

Figure S8. A) CVs for complexes **2a,** (red), **2b** (blue) and **2c** (yellow) in CH_2Cl_2 + 0.1 M $TBAPF_6$ *vs* SCE (reference electrode). Glassy carbon electrode (3 mm diameter) was used as working electrode. B) Cyclic voltammetry for the isomer **2a** in CH_2Cl_2 + 0.1M $TBAPF_6$ vs. SCE, starting the scanning at E_{init} = 1.20 V applying an equilibration time of 2 s (red) and 20 s (blue).

a)

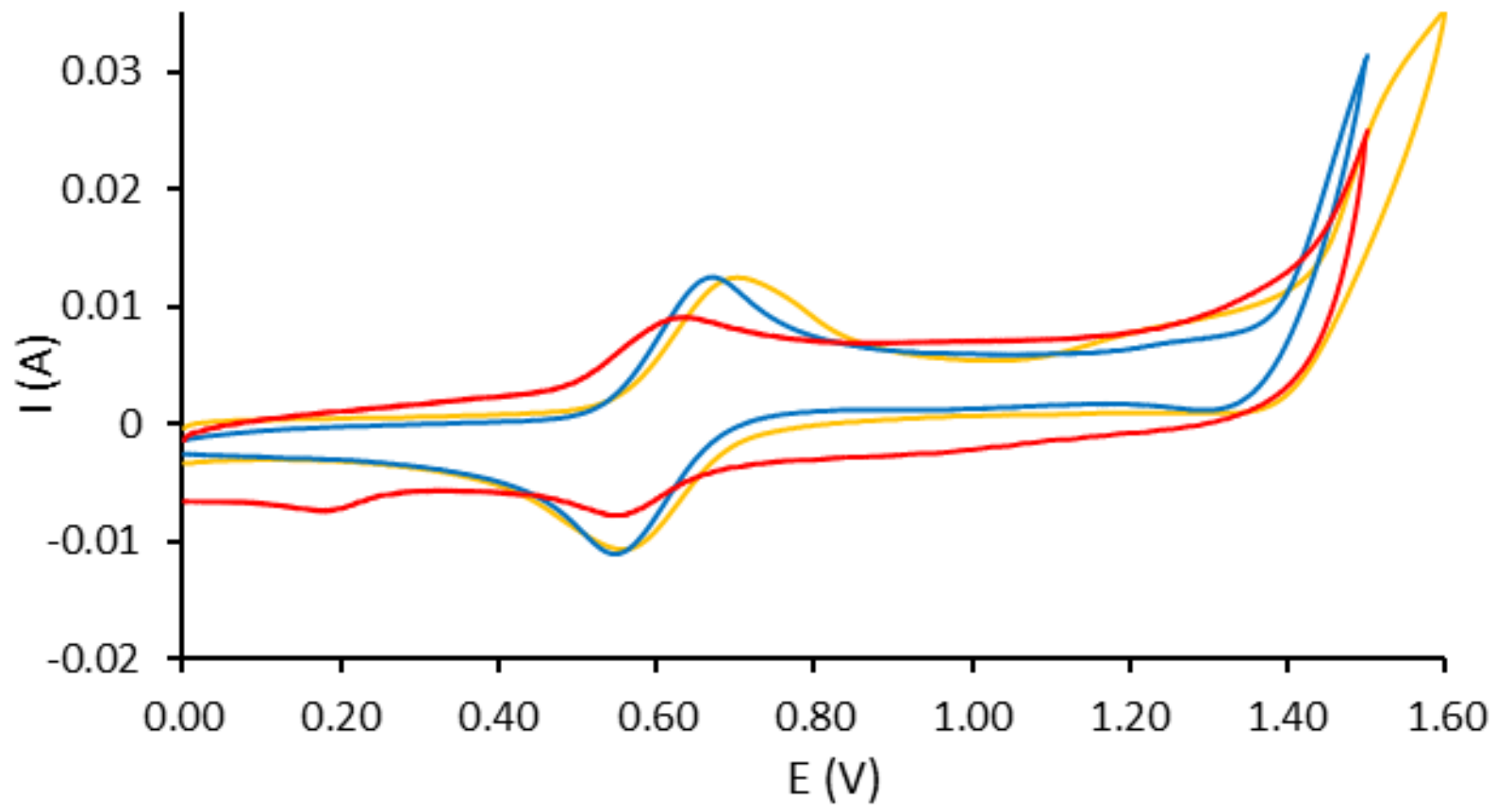

b)

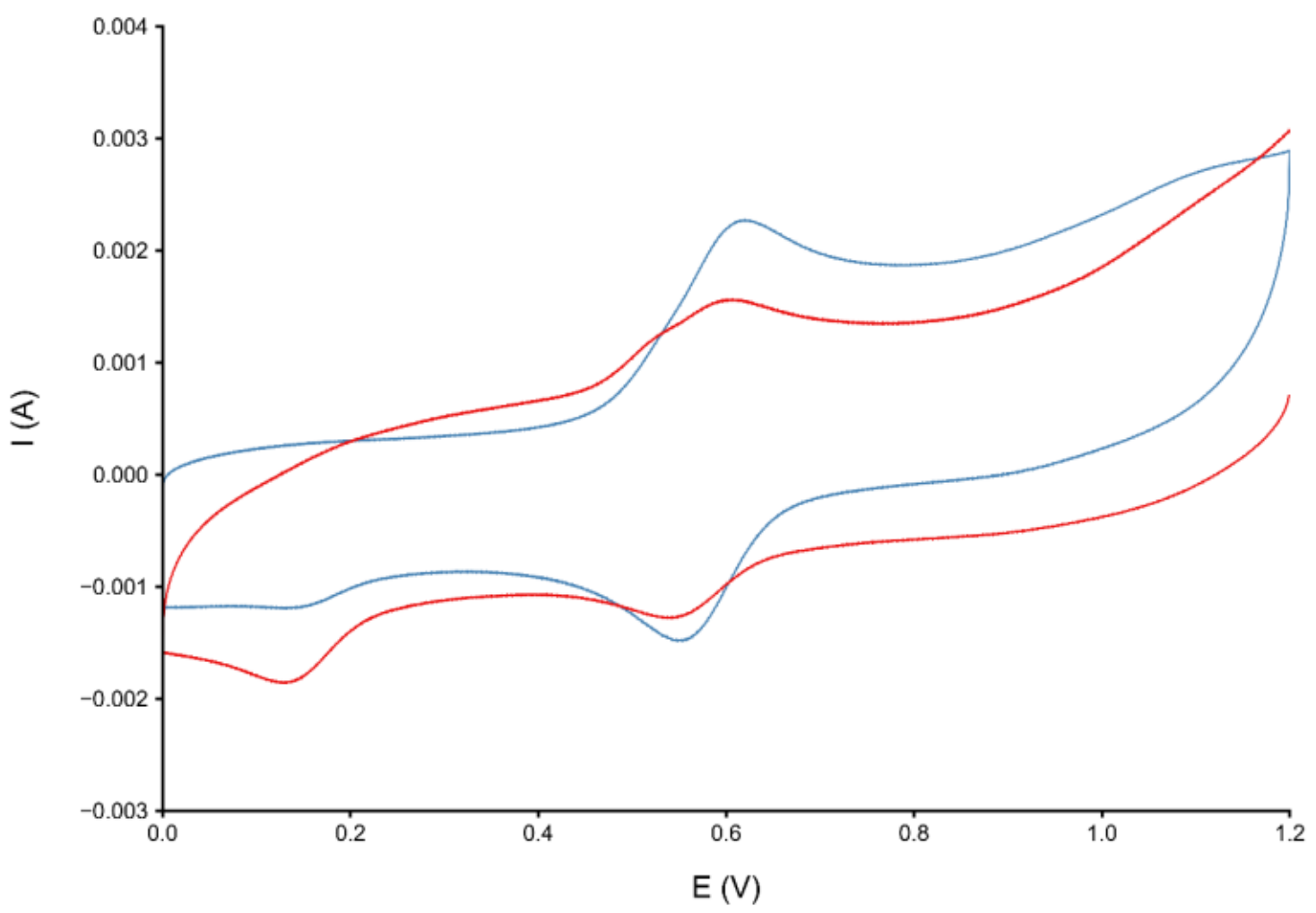

Figure S9. Gibbs free energy profile of the proposed mechanism for the isomerization of **2a** into **2c** via sulfoxide dissociation computed at Ωb97XD/def2-TZVP(H,C,N,O,S,Ru)/def2-TZVPD(Cl)//B3LYP/def2-SVP(H,C,N,O,S,Ru)/def2-SVPD(Cl) level, including solvent effects (ethanol) and quasi-harmonic enthalpic and entropic corrections at 353.15 K (in kcal mol^{-1}).

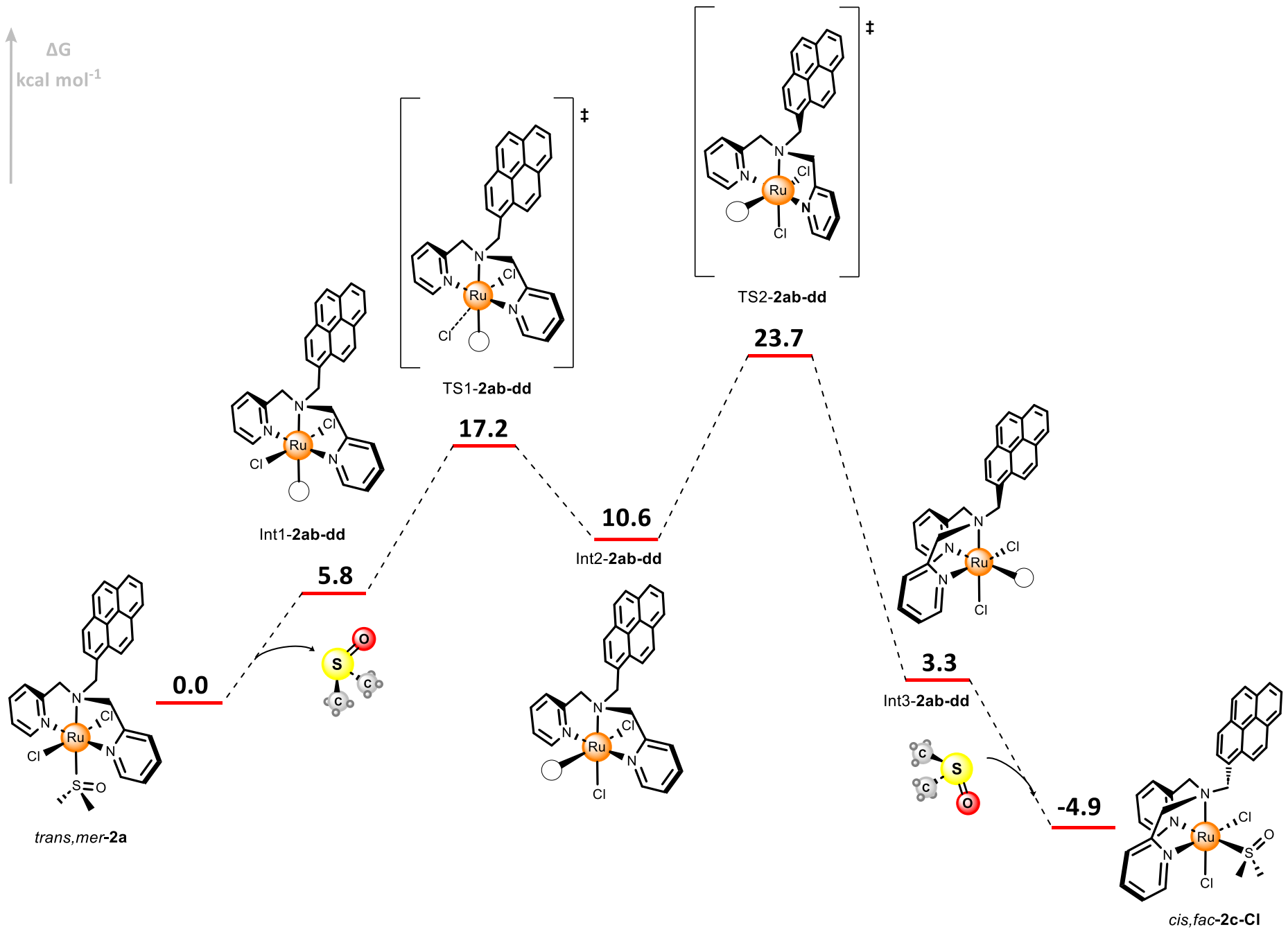

The dissociation of the sulfoxide ligand in the **2a** isomer generates the Int1-**2ab-dd** intermediate, which lies 5.8 kcal mol^{-1} higher in energy (Figure S9). The migration of the hloride ligand to the axial position leads to the formation of TS1-**2ab-dd**. This transition state presents a shift in the Cl1-Ru1-Cl2 angle from 165º to 127º, exhibits an imaginary frequency of -97 cm^{-1}, and lies at 17.2 kcal mol^{-1}. Further displacement to the vacant axial site stabilizes the molecule, yielding the intermediate Int2-**2ab-dd**, which lies at 10.6 kcal mol^{-1} and presents a narrow angle of 102º. At this point, the sulfoxide ligand could recoordinate to the vacant equatorial site, yielding the **2du** isomer, which is 1 kcal mol^{-1} above **2a**.

The rotation of the bpea-pyrene ligand in the Int2-**2ab-dd** intermediate proceeds through the TS2-**2ab-dd** transition state. This state presents an N5-Ru1-N6-N7 dihedral angle of 139.7º, an imaginary frequency of -151 cm^{-1}, and lies at 23.7 kcal mol^{-1}. Further rotation of the pyridine ring to an N5-Ru1-N6-N7 dihedral angle of 101º yields the Int3-**2ab-dd** intermediate at 3.3 kcal mol^{-1}. At this step, it is possible for the coordination of the sulfoxide ligand to the equatorial vacant position, yielding the **2c** isomer, which lies 4.9 kcal mol^{-1} below **2a**.

Figure S10. Gibbs free energy profile of the proposed mechanism for the isomerization of *trans,fac*-**2b** into the *cis,fac*-**2c** isomer via chloride dissociation computed at Ωb97XD/def2-TZVPD(H,C,N,O,S,Ru)/def2-TZVPD(Cl)//B3LYP/def2-SVP(H,C,N,O,S,Ru)/def2-SVPD(Cl) level, including solvent effects (ethanol) and quasi-harmonic enthalpic and entropic corrections at 353.15 K (in kcal/mol^{-1}).

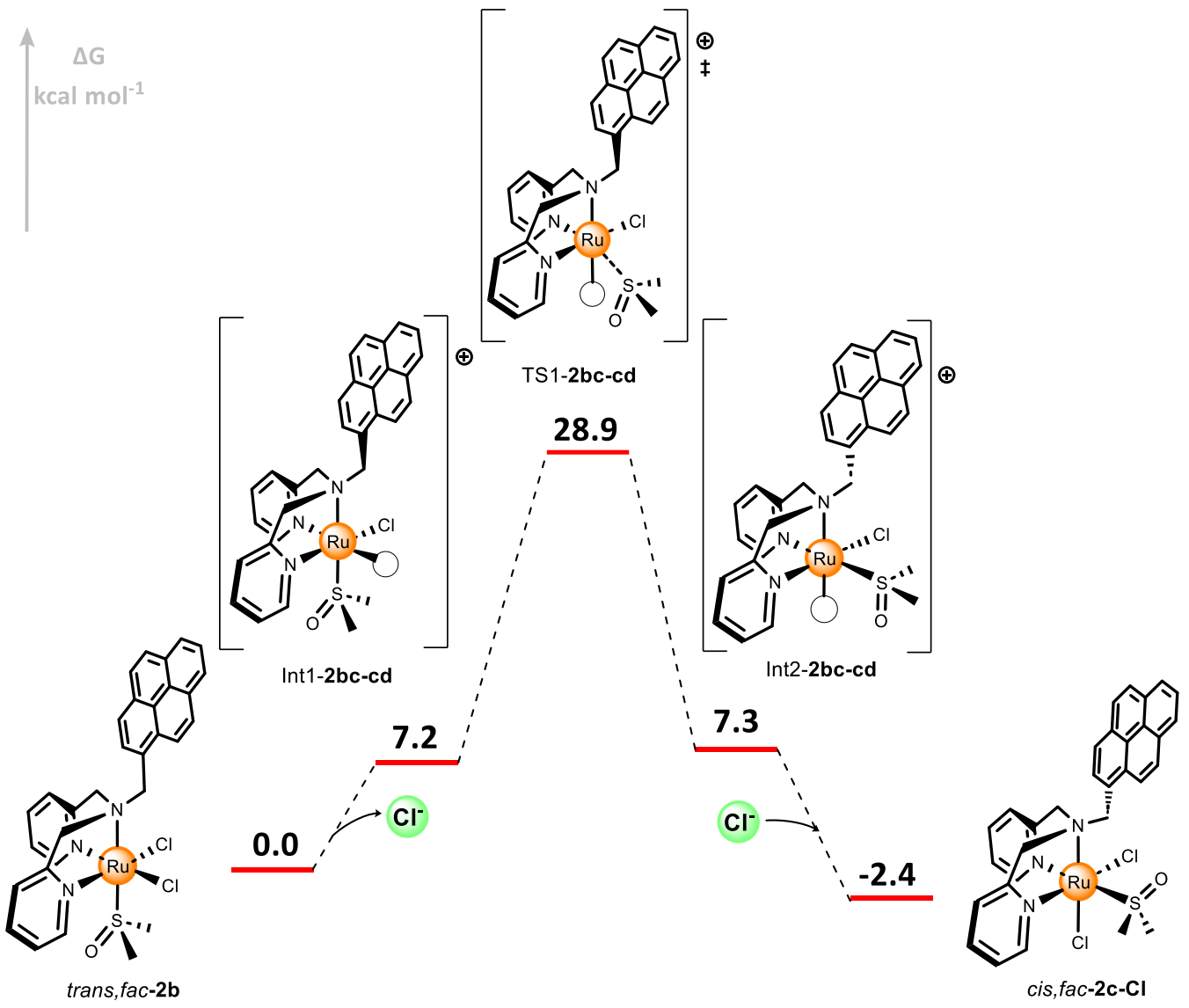

The chlorido ligand dissociation on the **2b** isomer yields the intermediate Int1-**2bc-cd**, which lies 7.2 kcal mol^{-1} higher in energy. The migration of the sulfoxide ligand to the vacant equatorial site leads to the formation of the transition state TS1-**2bc-cd**. This state presents a N5-Ru1-S1 angle of 142.0º, an imaginary frequency of -135 cm^{-1}, and lies at 28.9 kcal mol^{-1}. Continued migration of the sulfoxide ligand to the vacant site generates the intermediate Int2-**2bc-cd** when the N5-Ru1-S1 angle is 174.5º, which lies at 7.3 kcal mol^{-1}. The coordination of the chlorido ligand to the axial position yields the **2c-Cl**, which is 2.4 kcal mol^{-1} more stable than the **2b** isomer.

Comparing both mechanisms, it can be concluded that the isomerization in ethanol proceeds via the dissociation of the sulfoxide ligand. This is evidenced by the fact that the TS1-**2bc-dd** lies at 12.5 kcal mol^{-1} while TS1-**2bc-cd** lies significantly higher at 28.9 kcal mol^{-1}. This energetic difference can be attributed to two main factors. First, the migration of the sulfoxide ligand is energetically more costly due to its bulkiness compared with the chlorido ligand. Second, the use of a protic solvent such as ethanol stabilizes strong H-acceptor species such as sulfoxide. In contrast, the release of a charged chloride anion into the reaction medium is highly unfavored under these conditions.

Figure S11. Molecular structure and labelling schemes for *mer*-**3a**. The structure shows an octahedral geometry with a meridional disposition of both the bpea-pyrene ligand and the three hloride ligands.

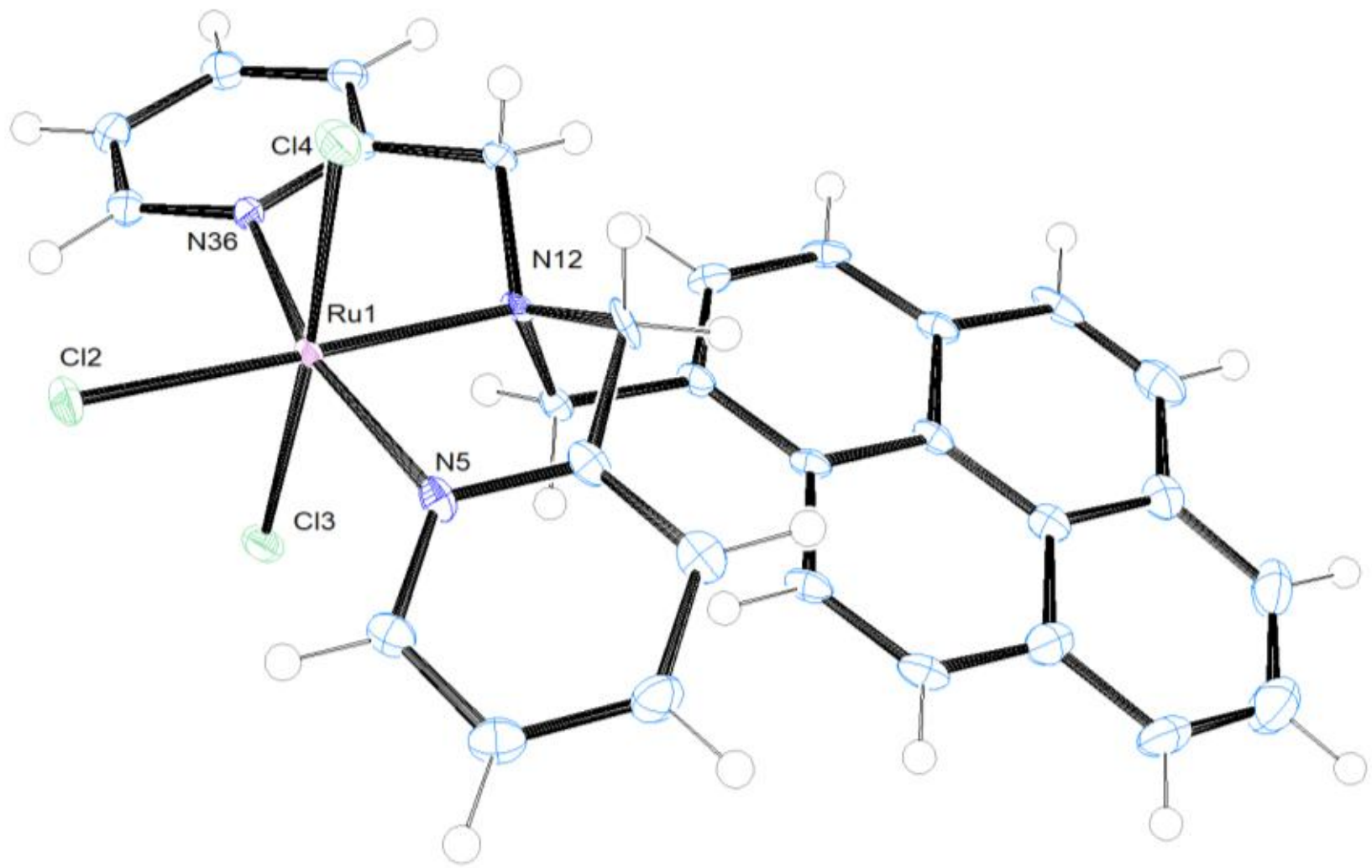

Figure S12. Cyclic voltammetry preformed after irradiation of a) **2a** and b) **2c** in CH_2Cl_2 + 0.1 M $TBAPF_6$ vs. SCE starting the scanning at E_{init} = 1.20 V.

a)

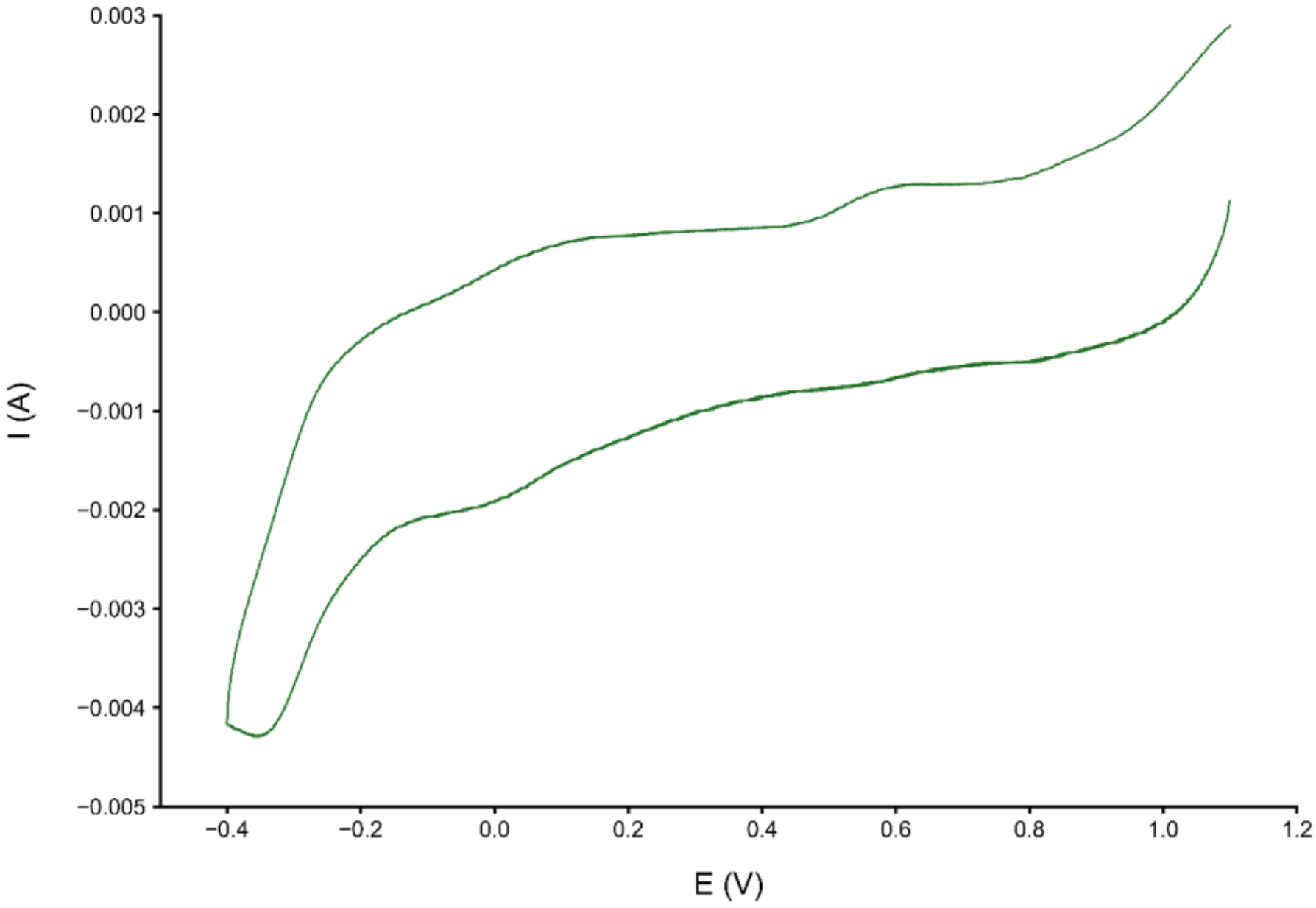

b)

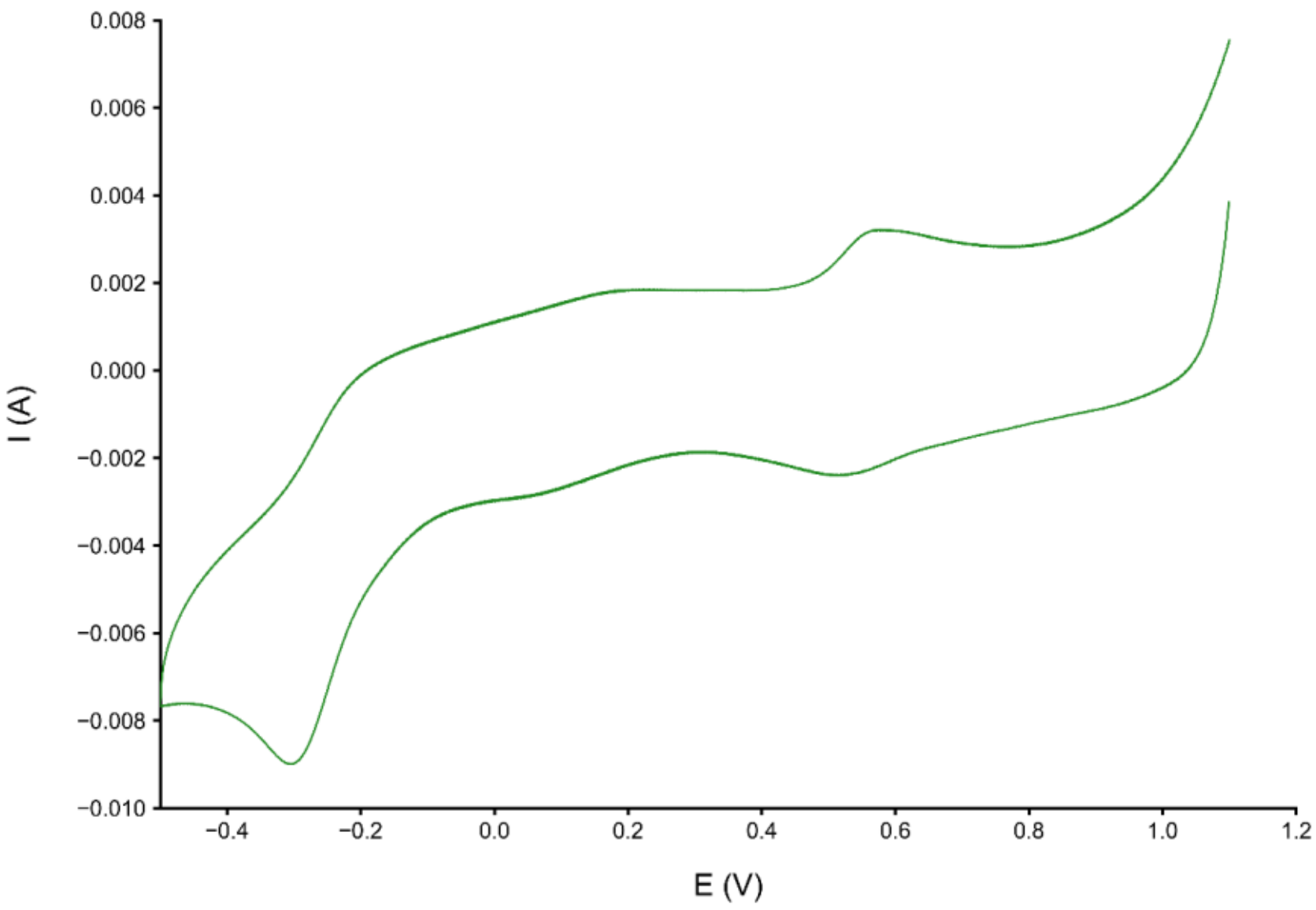

Figure S13. A) UV-vis spectra corresponding to the photochemical transformation of **2a** upon 3 h of light irradiation and b) **2c** after 2.5 h (green t= 0 h; violet t= 2.5 h)**,** in H_2O; c) CV corresponding to the photochemical transformation **2a** after 3 h of irraciation in phosphate tampon Ph 7(t= 0 h green line; t= 4 h blue line).

a)

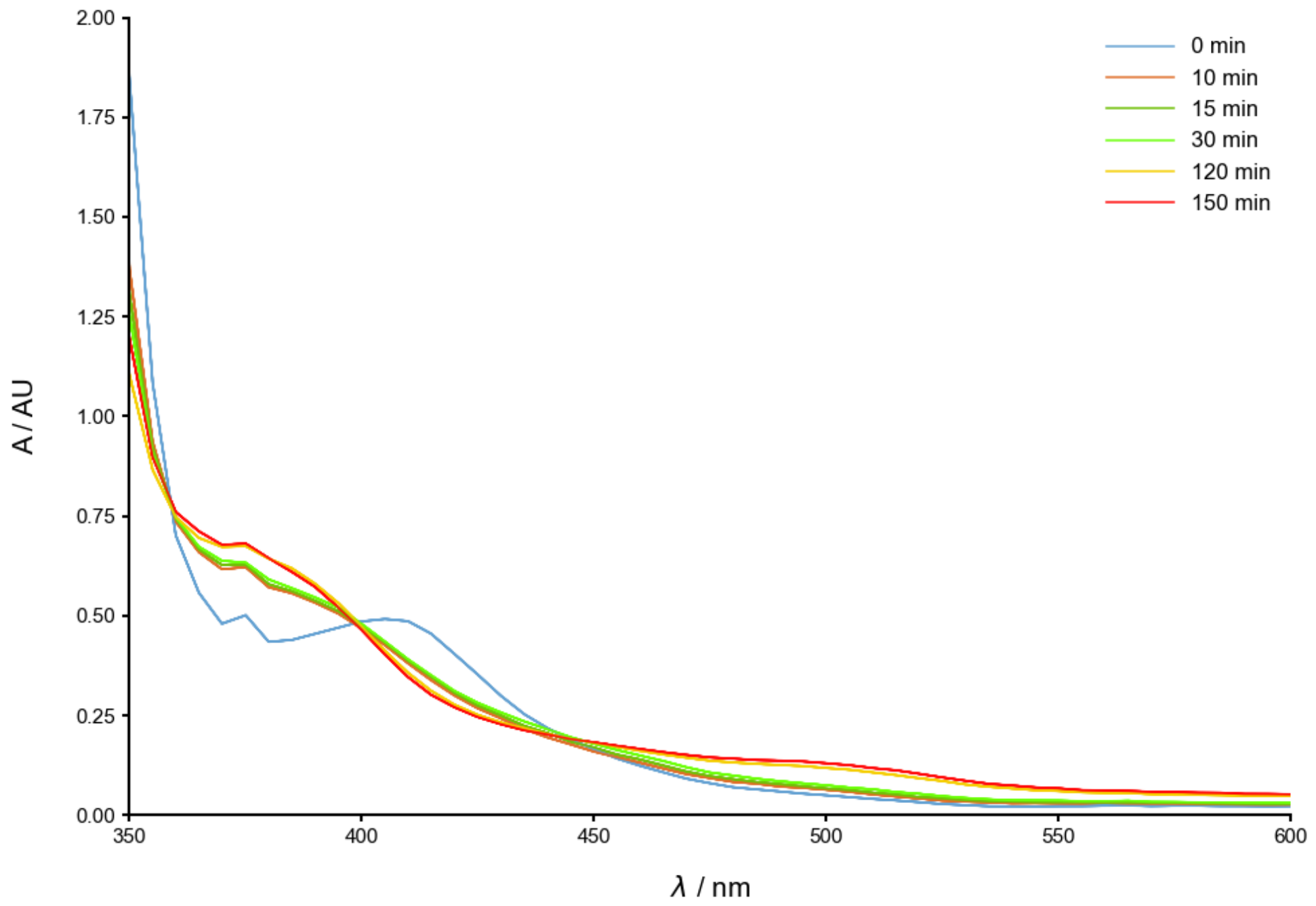

b)

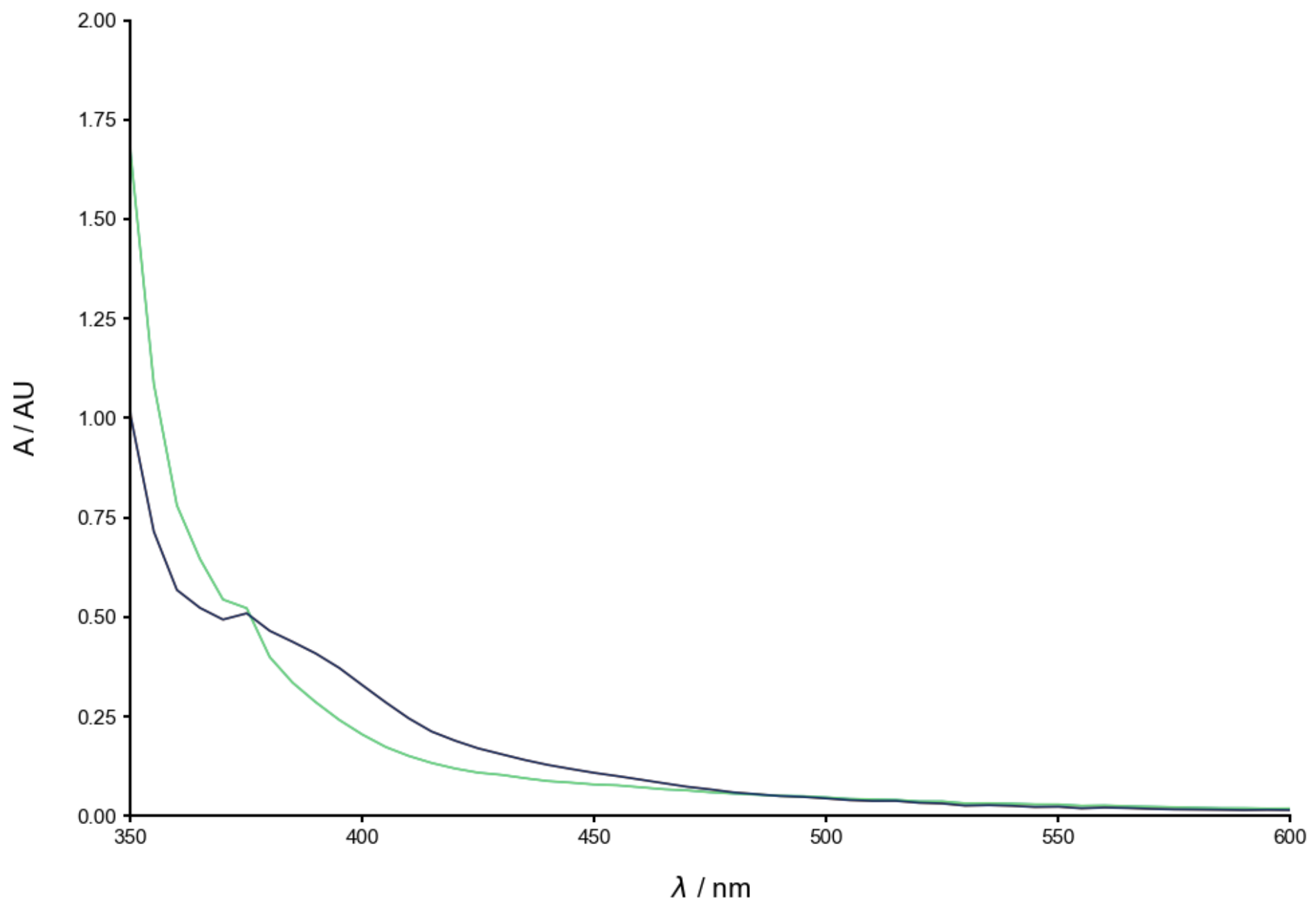

c)

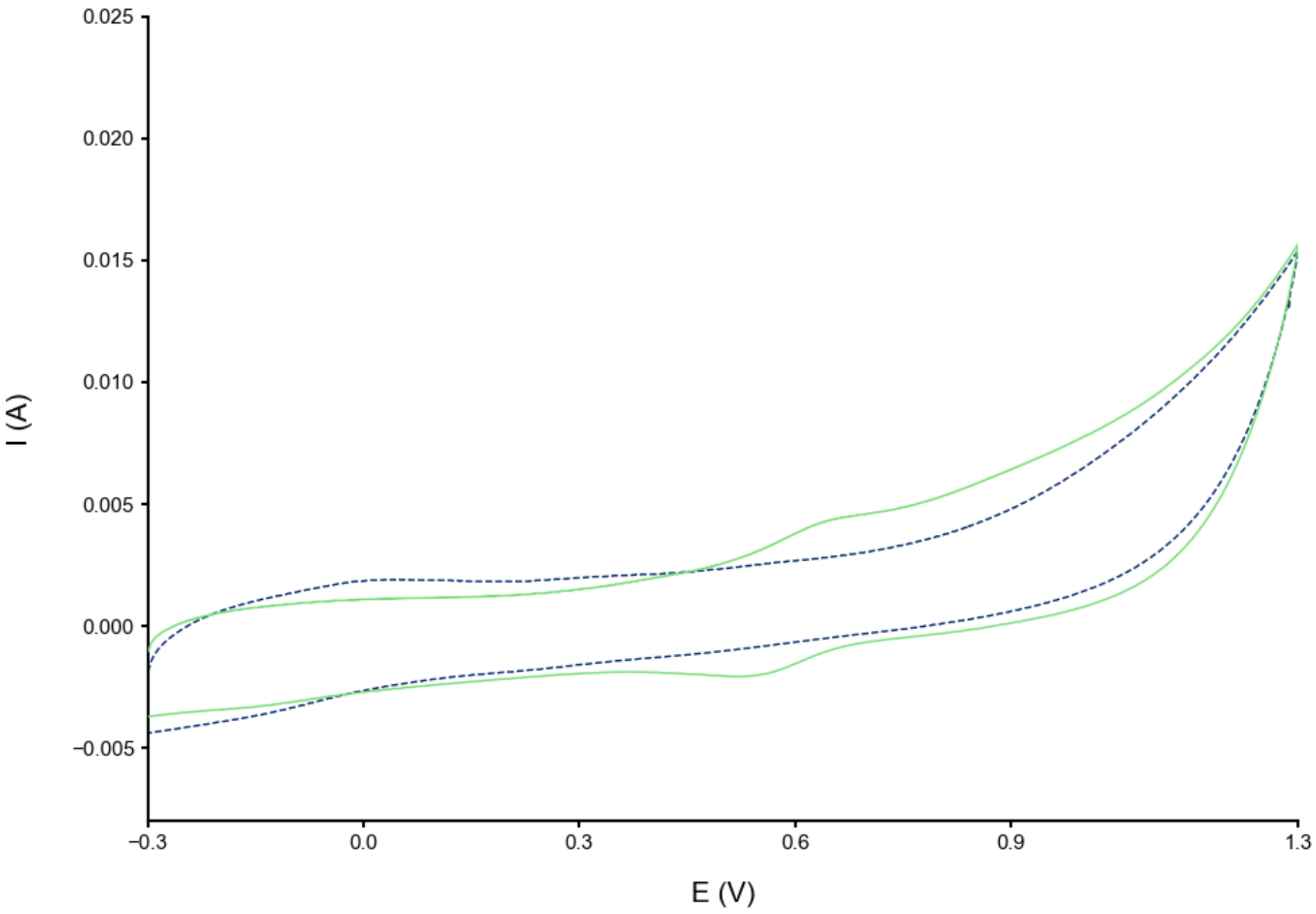

Figure S14. A) UV-vis experiment corresponding to the photoinduced transformation of **2a** at RT in CH_3CN after 60 min; b) CV corresponding to the photoinduced transformation of **2a** at RT in CH_3CN + 0.1 M $TBAPF_6$ vs. SCE after 60 min.

a)

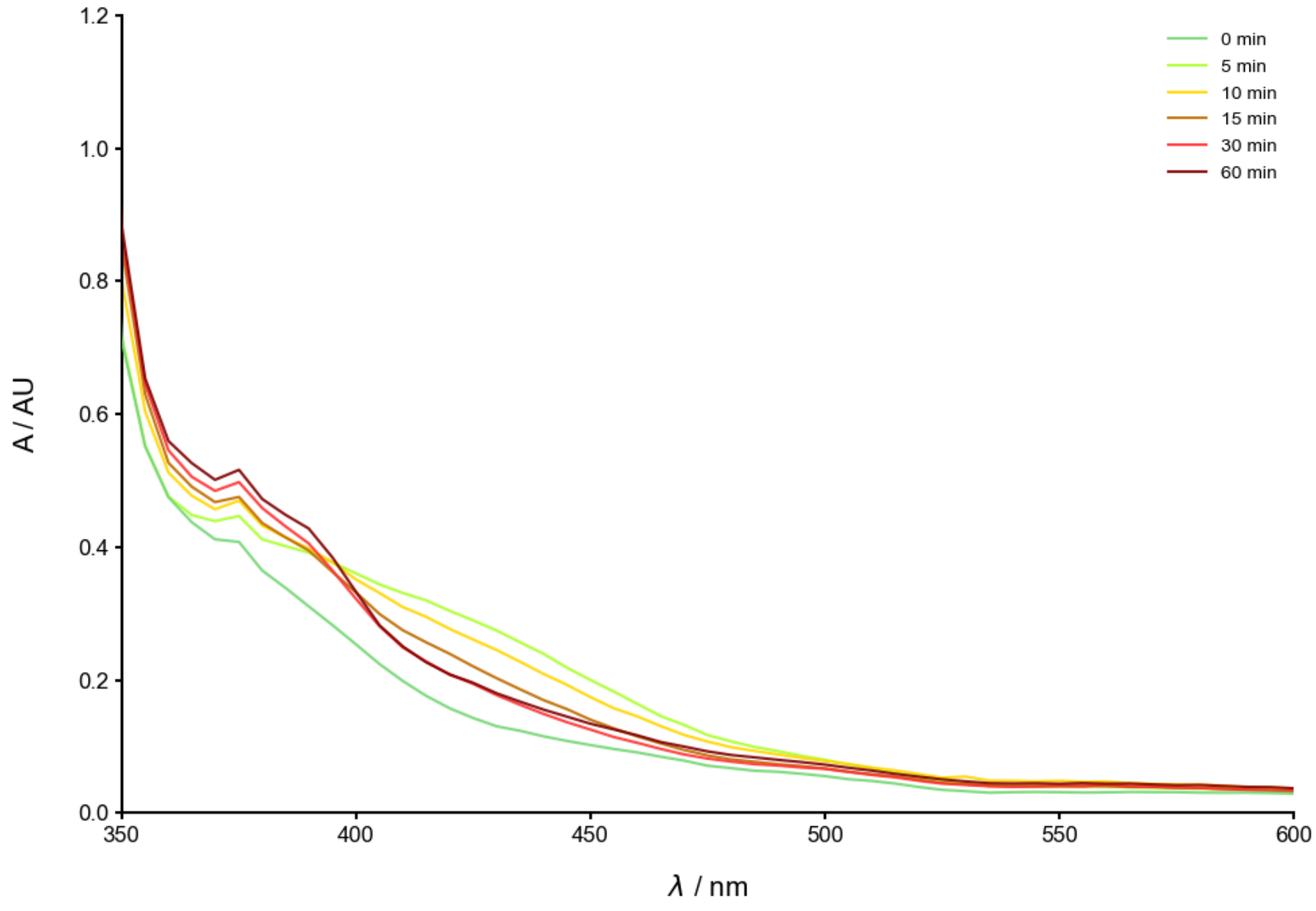

b)

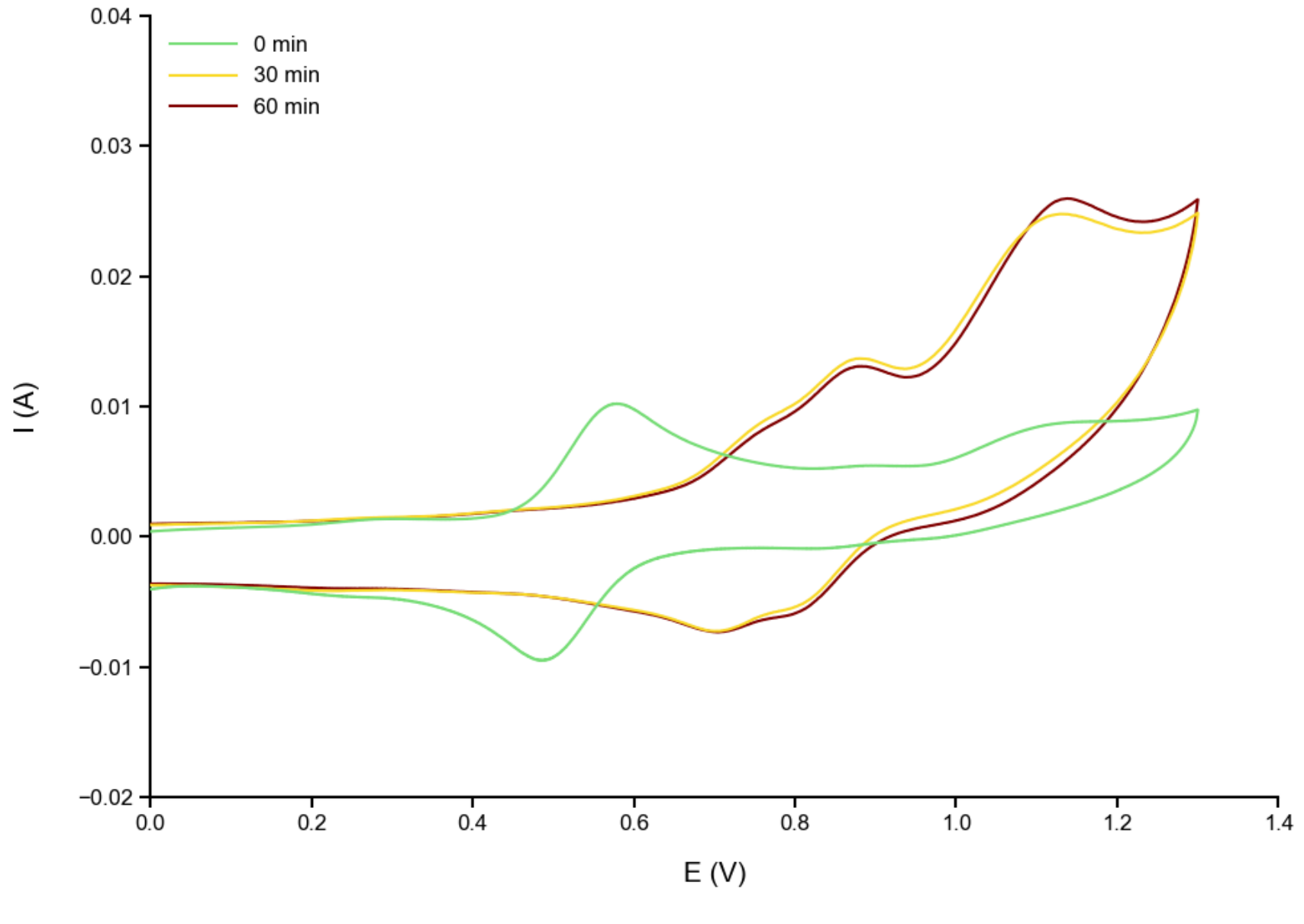

Figure S15. A) UV-vis spectra corresponding to the thermal transformation of **2c** in H_2O at 60 ºC after 6 h; b) Superposition of UV-vis spectra resulting from photo and thermo substitution in water of **2c** and **2a**, respectively, leading to the formation of the compound *cis*,*fac*-[$Ru^{II}Cl_2$(bpea-pyrene)(OH_2)], *cis*,*fac*-**4c**.

a)

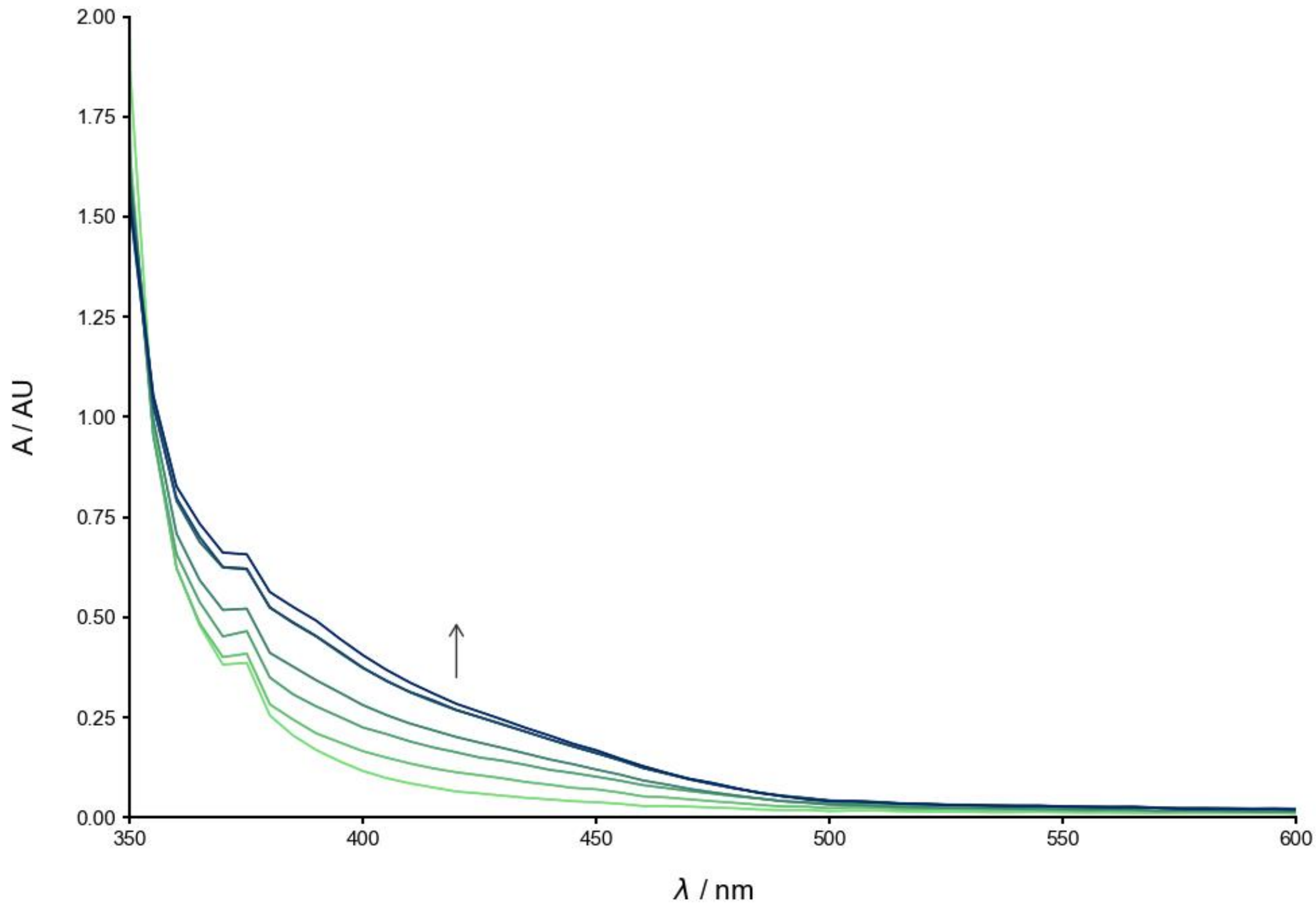

b)

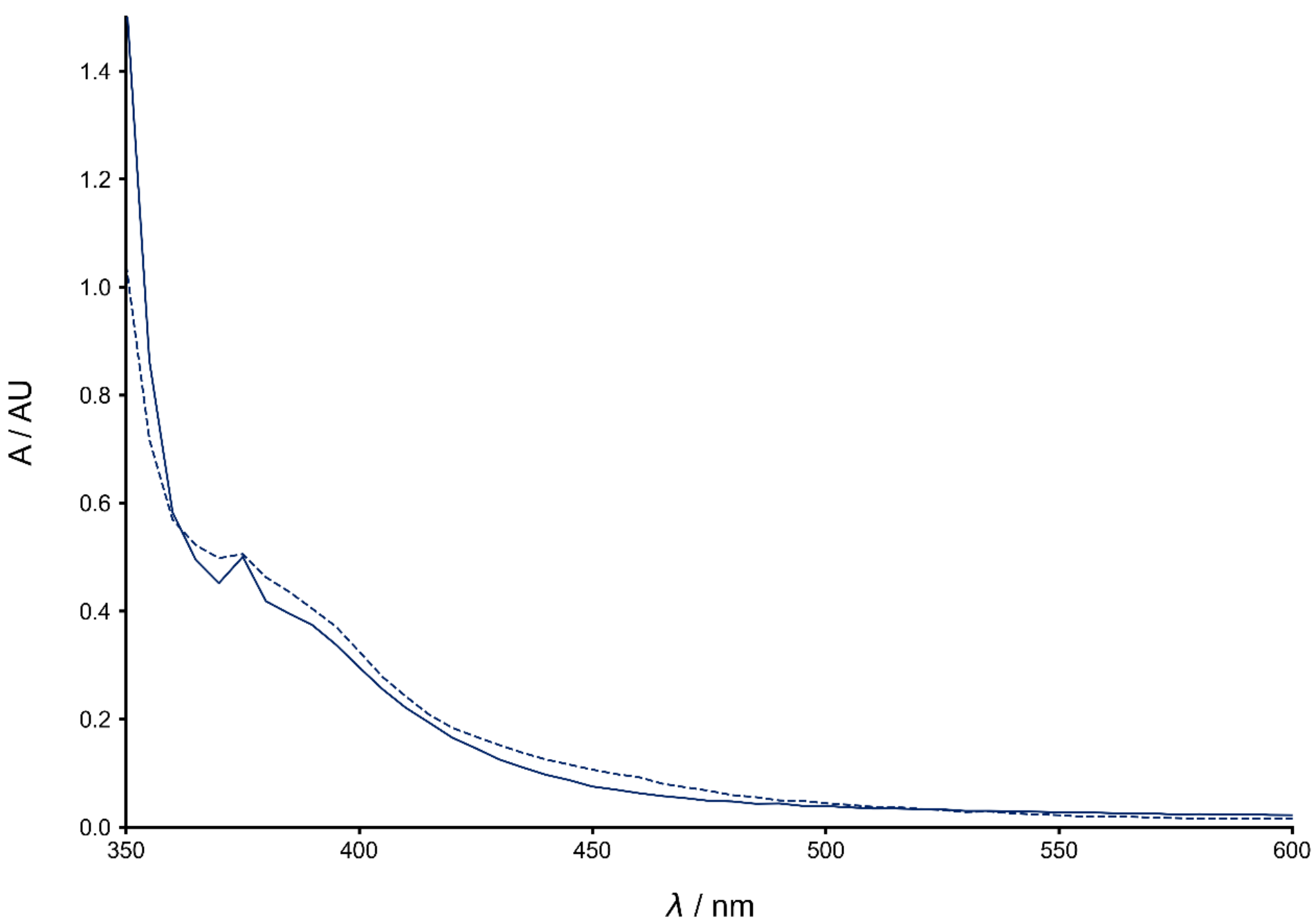

Figure S16. A) CV in CH_3CN and b) ESI-MS of *cis,fac*-**5s**.

a)

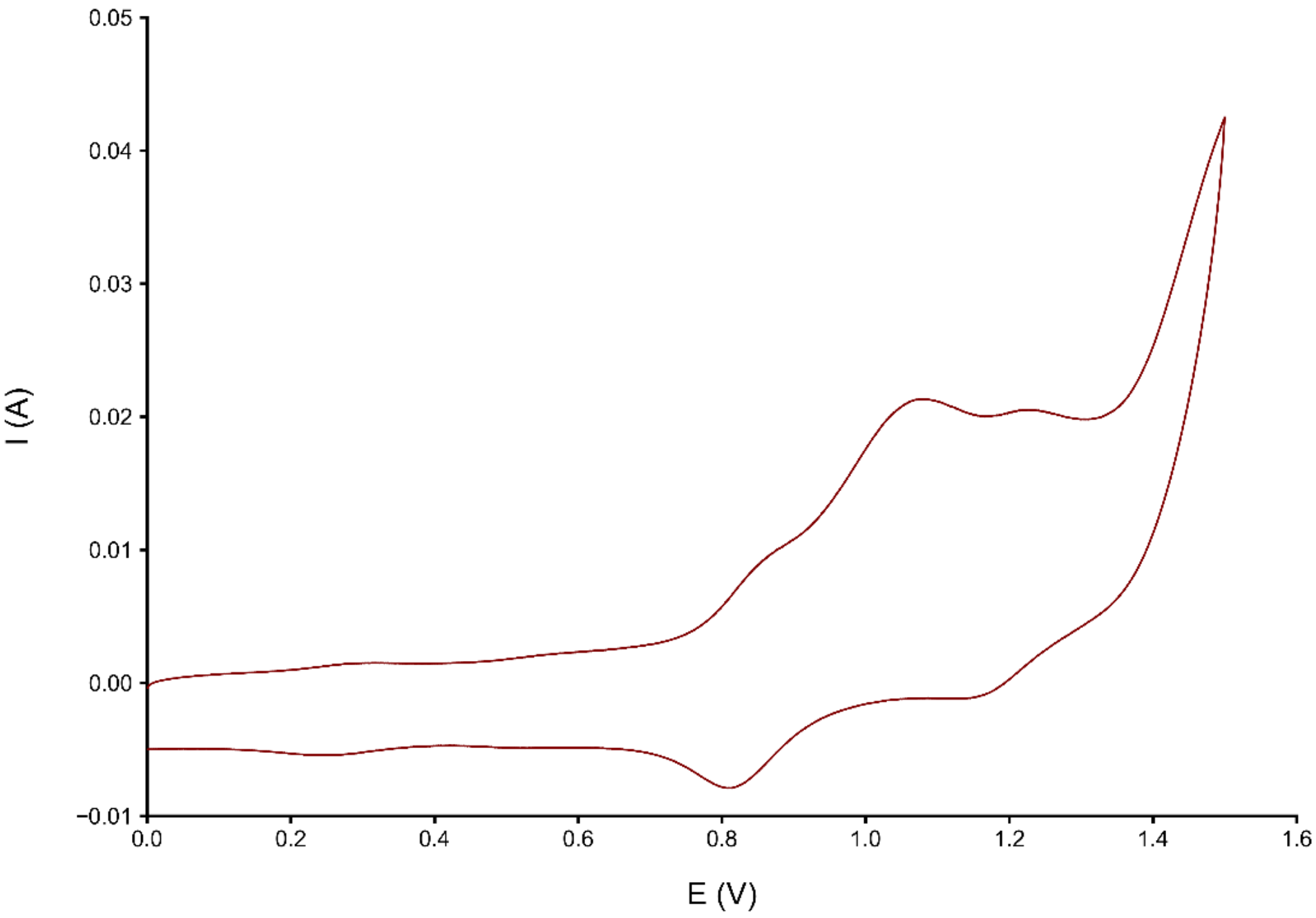

b)

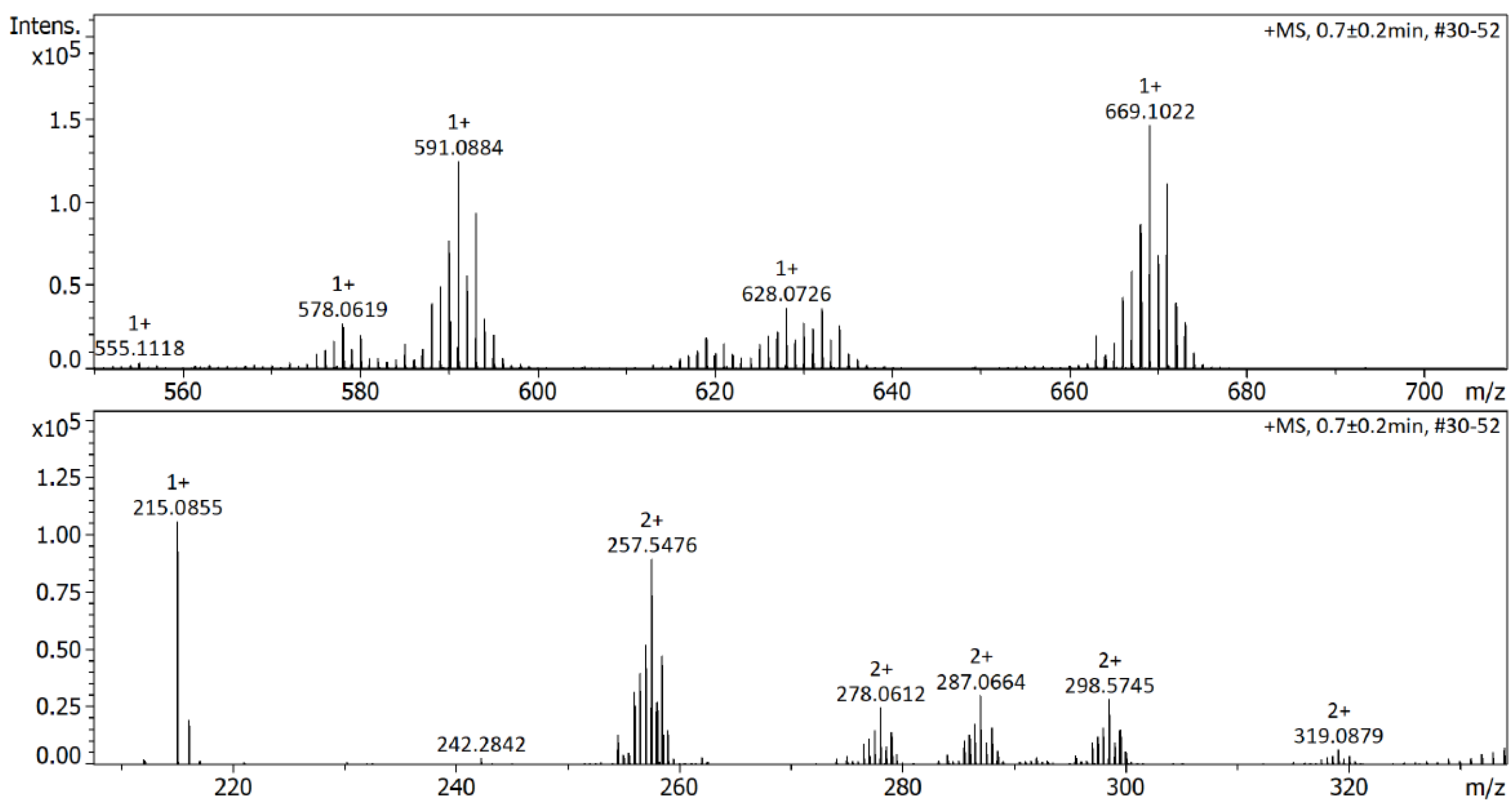

Figure S17. Thermogravimetric analysis of naked GO (black line) and GO@**2c** (yellow line).

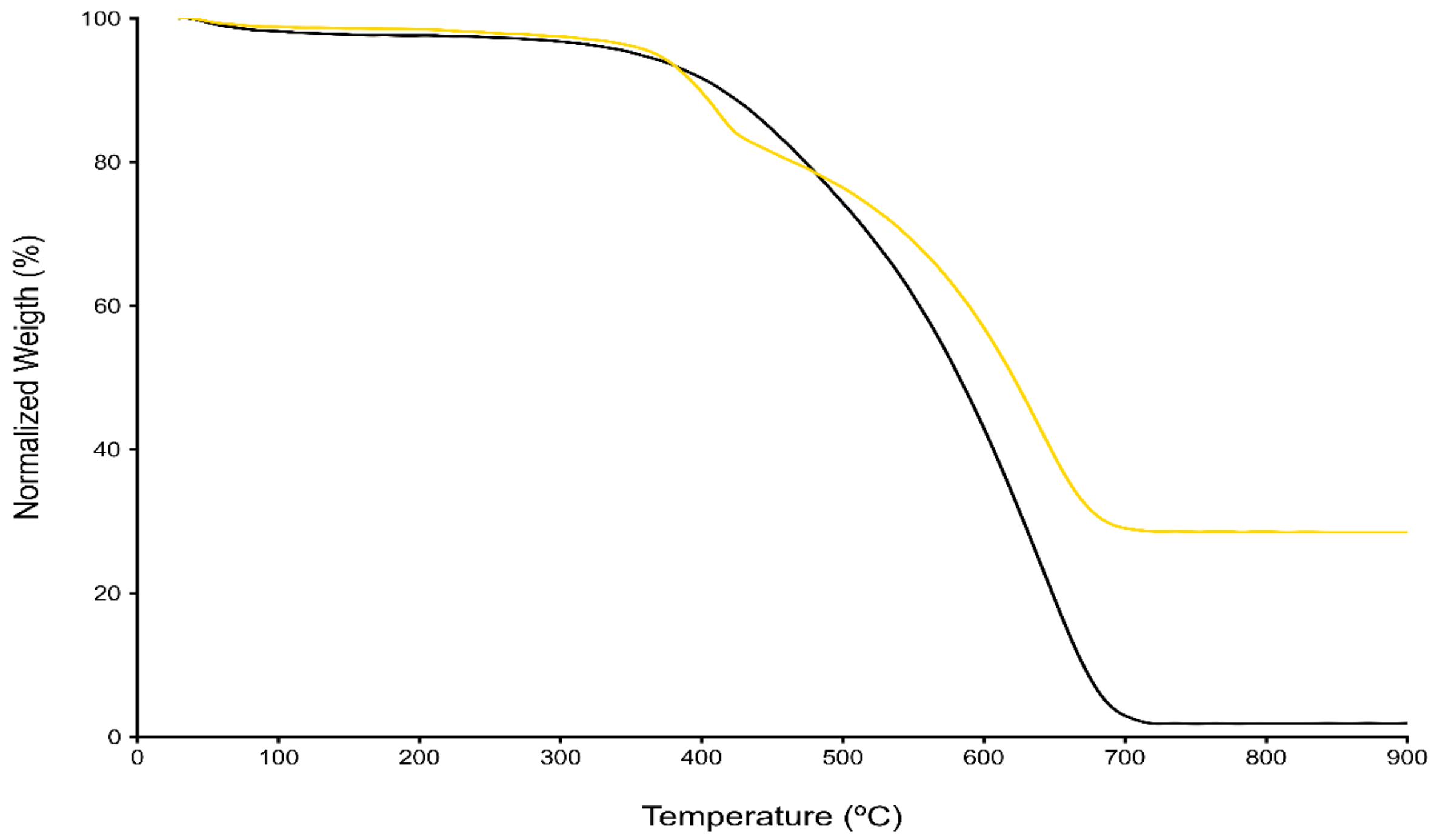

Figure S18. TEM images of GO@**2c**. The bars indicate a length of 100 nm.

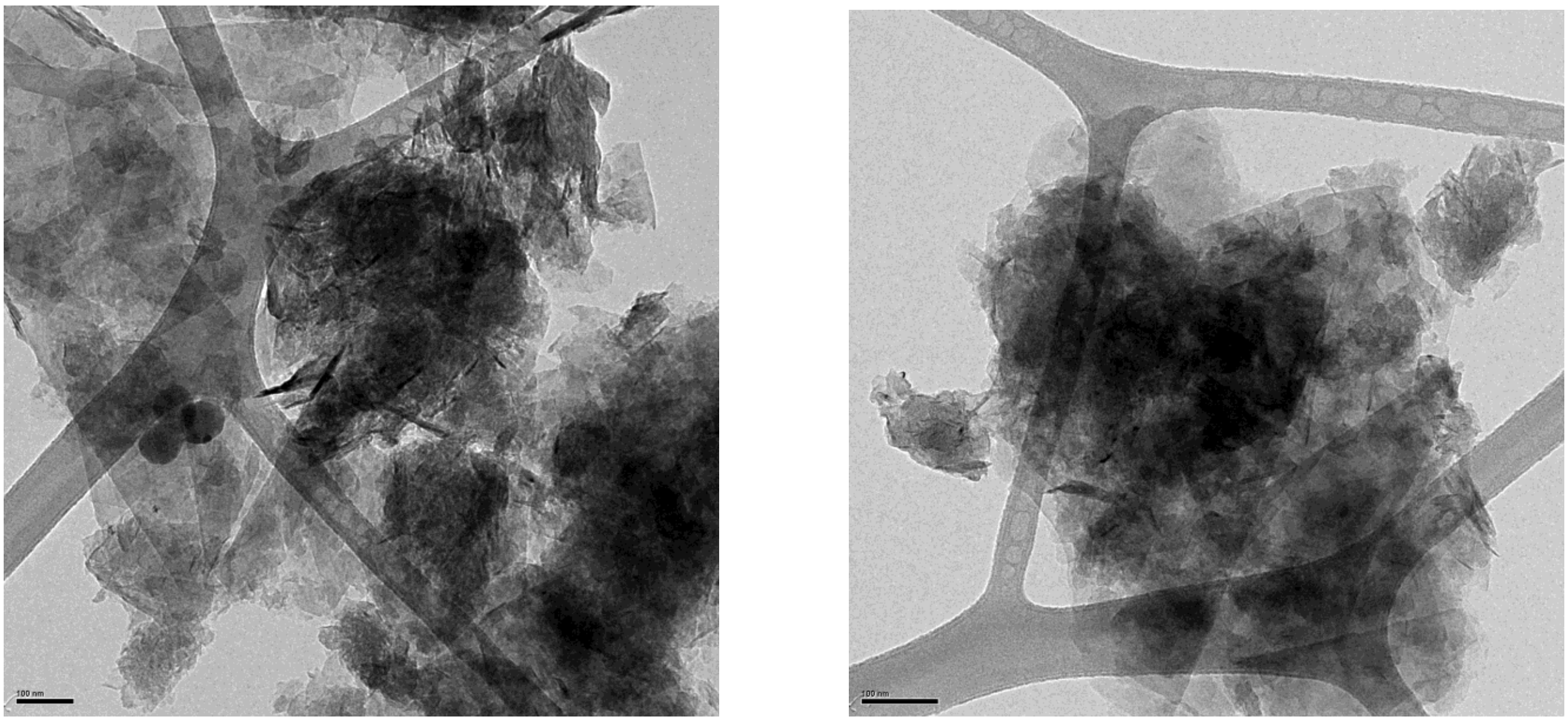

Figure S19. A) XPS spectra of GO@**2c**; Magnification of the XPS spectrum in b) the O1s, c) the N1s and d) the C1s regions.

a)

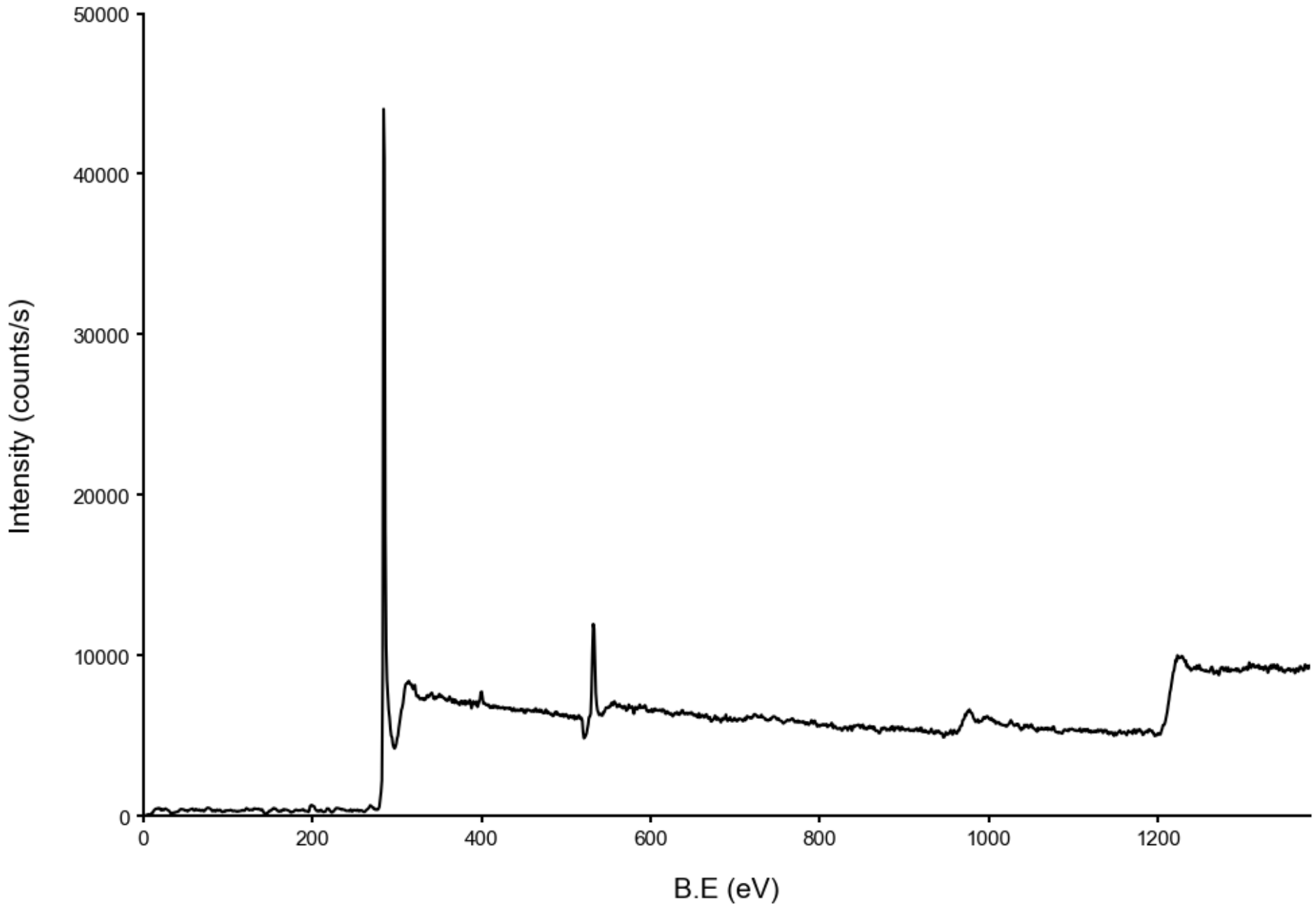

b)

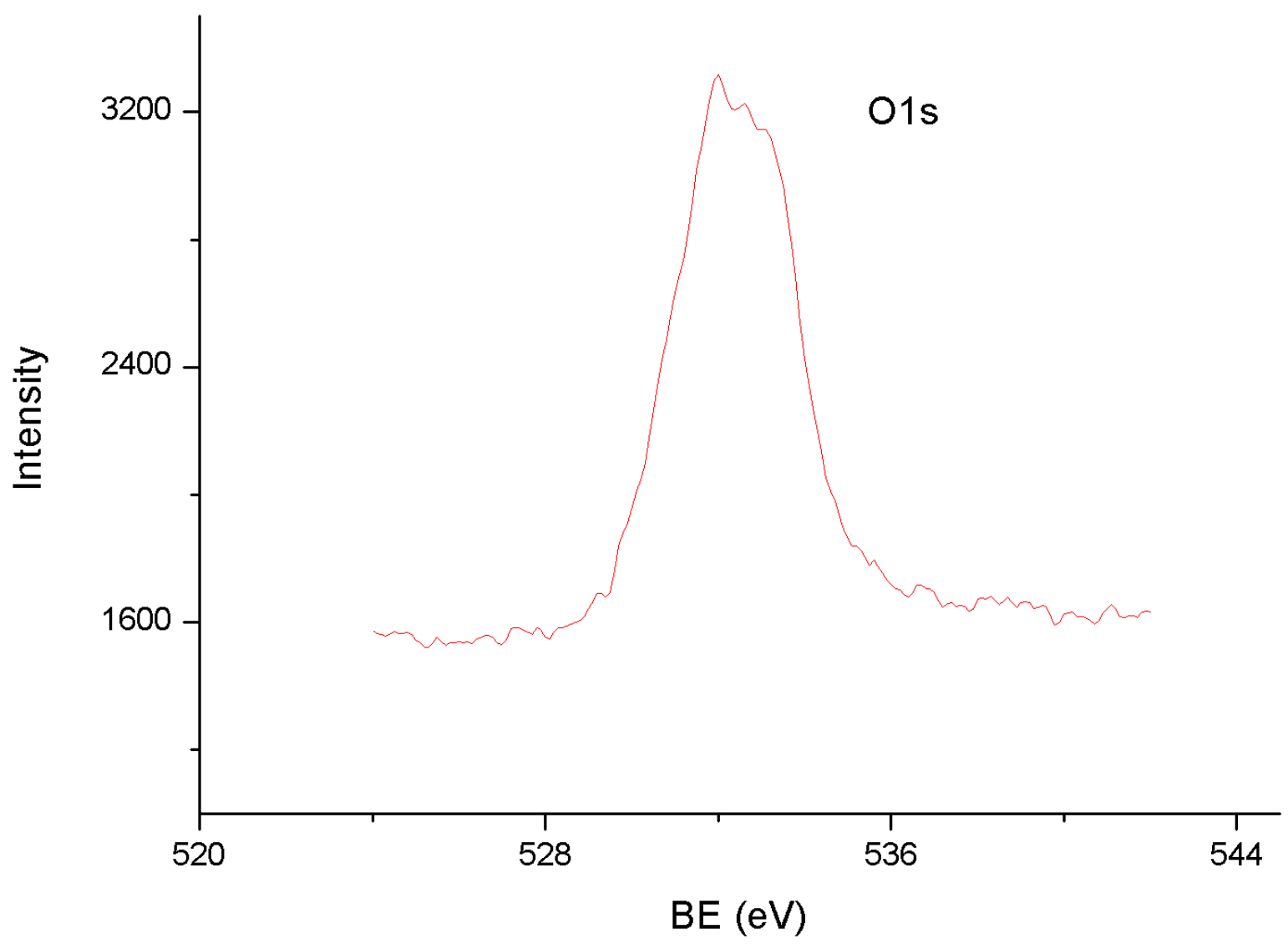

c)

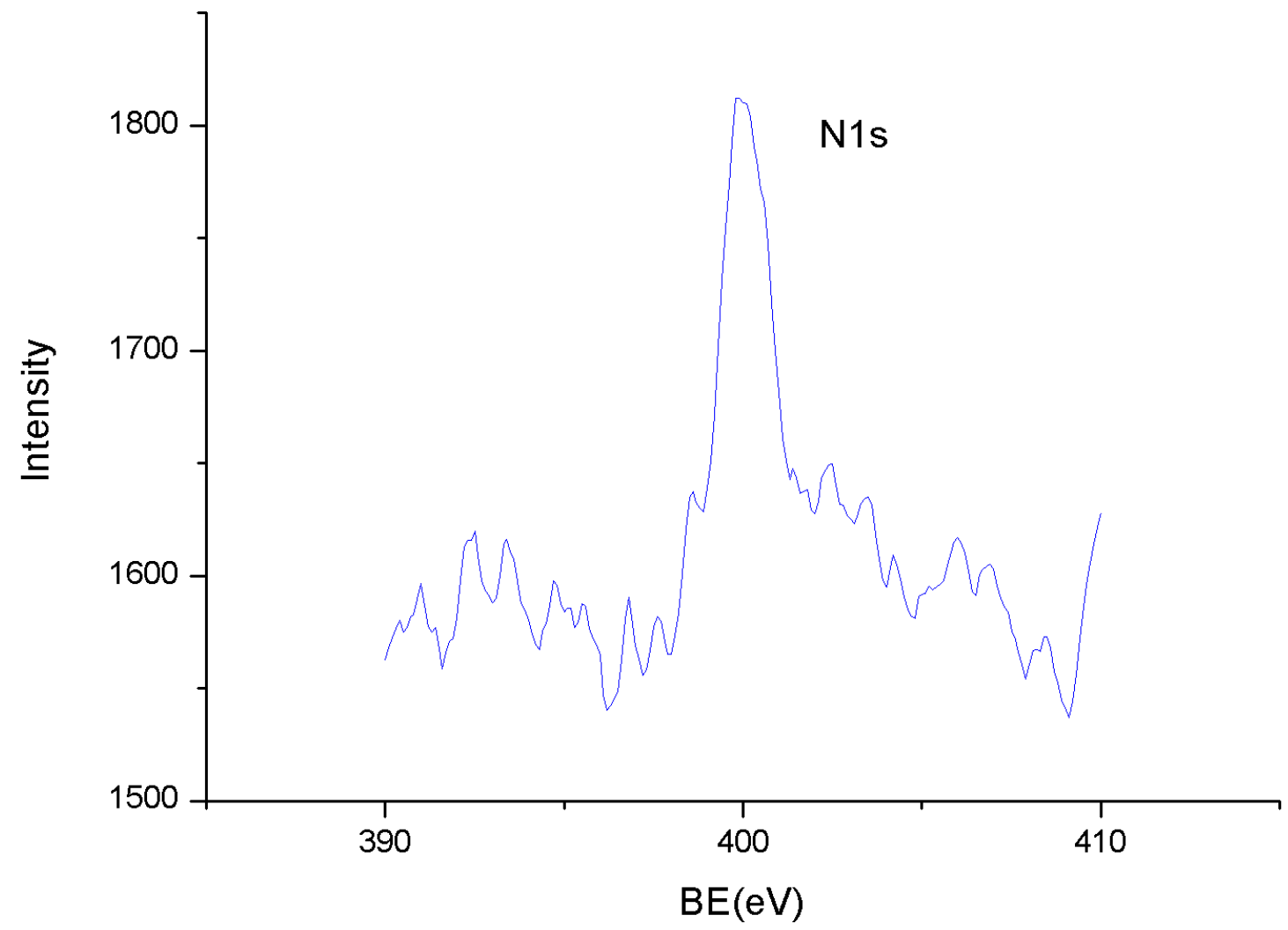

N1s
Intensity
1800
1700
1600
1500
390
400
410
BE(eV)

d)

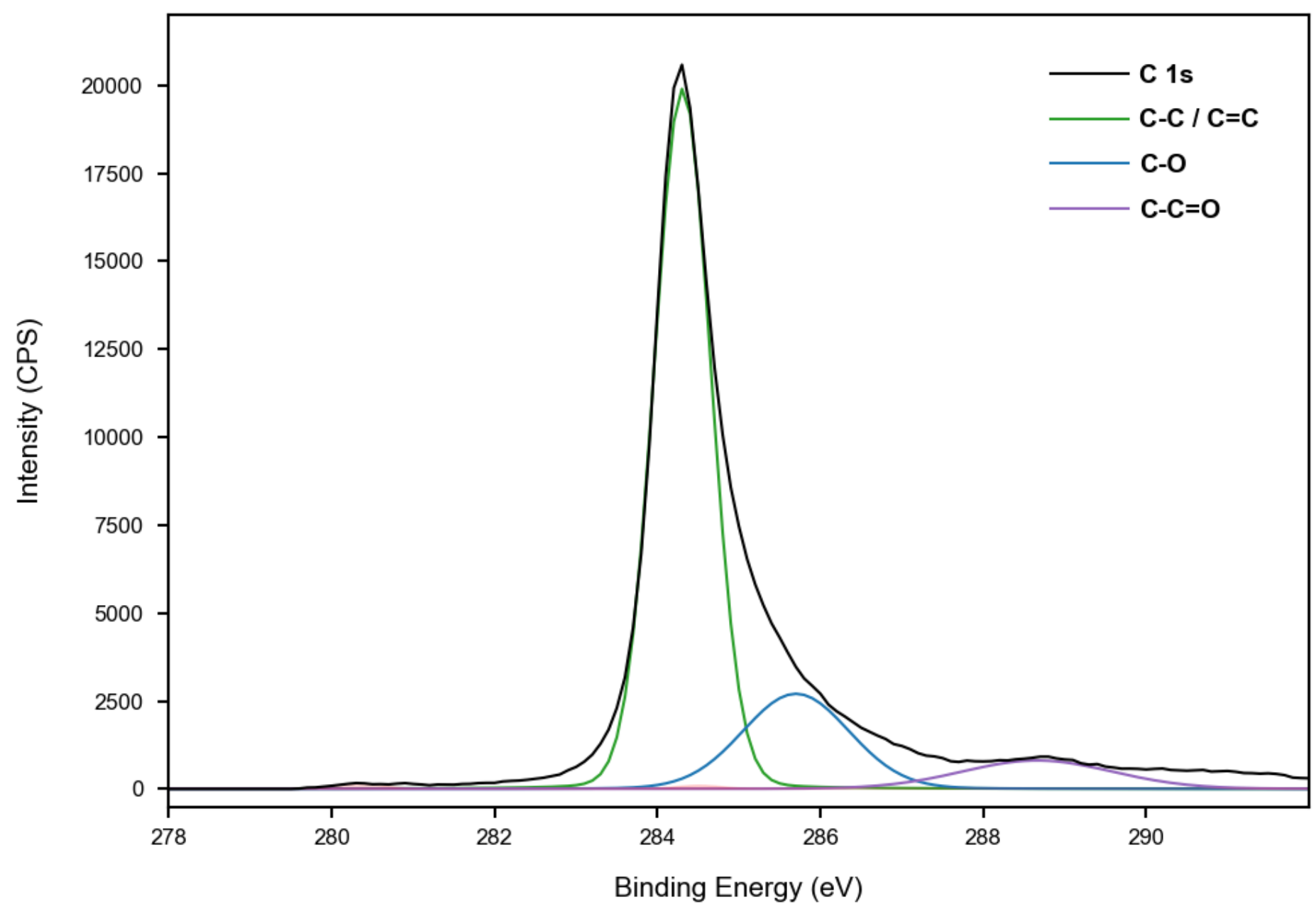

C 1s
C-C / C=C
C-O
C-C=O
Intensity (CPS)
20000
17500
15000
12500
10000
7500
5000
2500
0
278
280
282
284
286
288
290
Binding Energy (eV)

Figure S20. DPV of a) **2c** (blue line), GO@**2c** (red line) and GO (green line) and b) GO@**2c** (red line) and GO (green line) in CH_2Cl_2 +$TBAPF_6$ 0.1 M *vs* SCE (reference electrode). Glassy carbon electrode (3 mm diameter) was used as working electrode.

a)

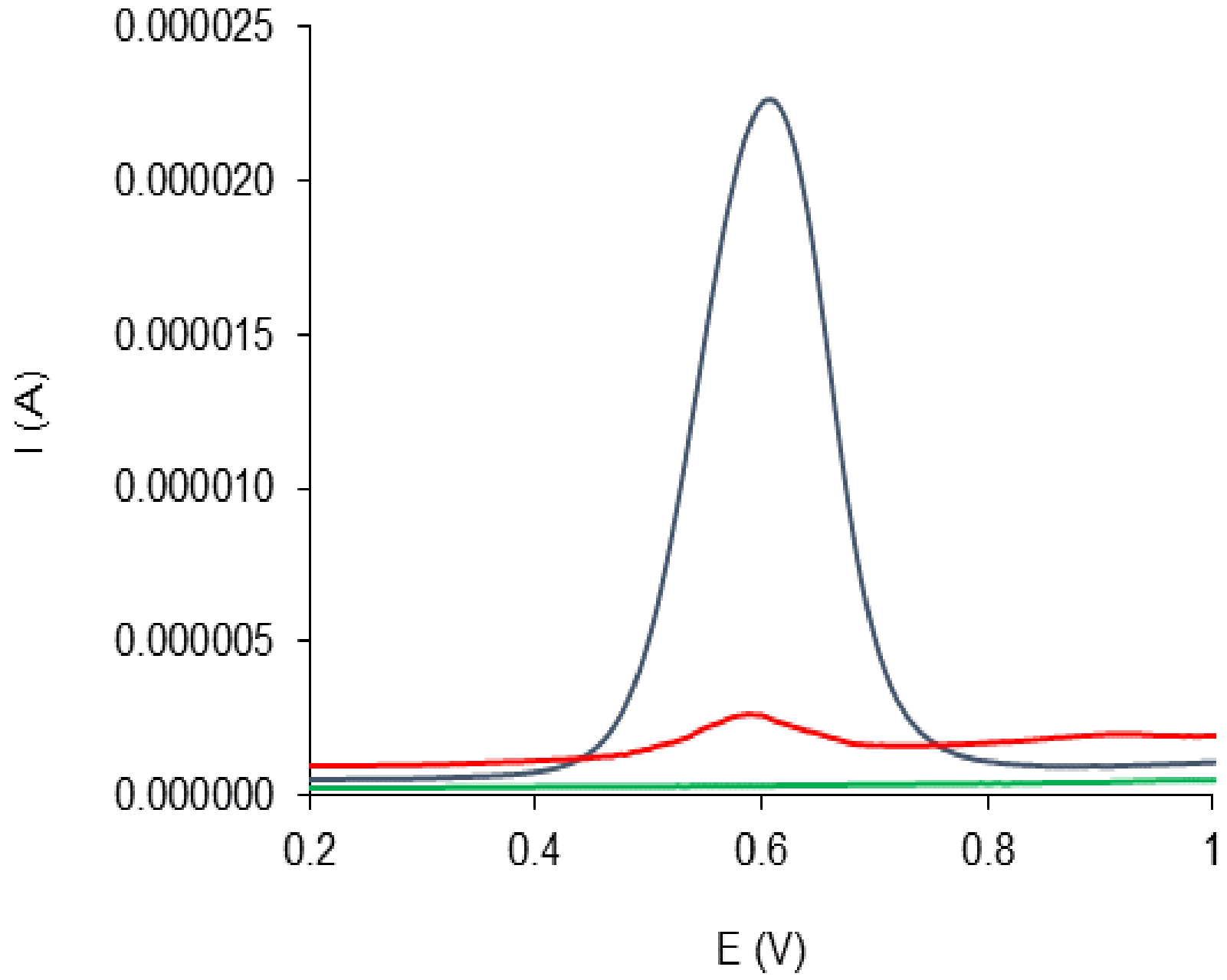

b)

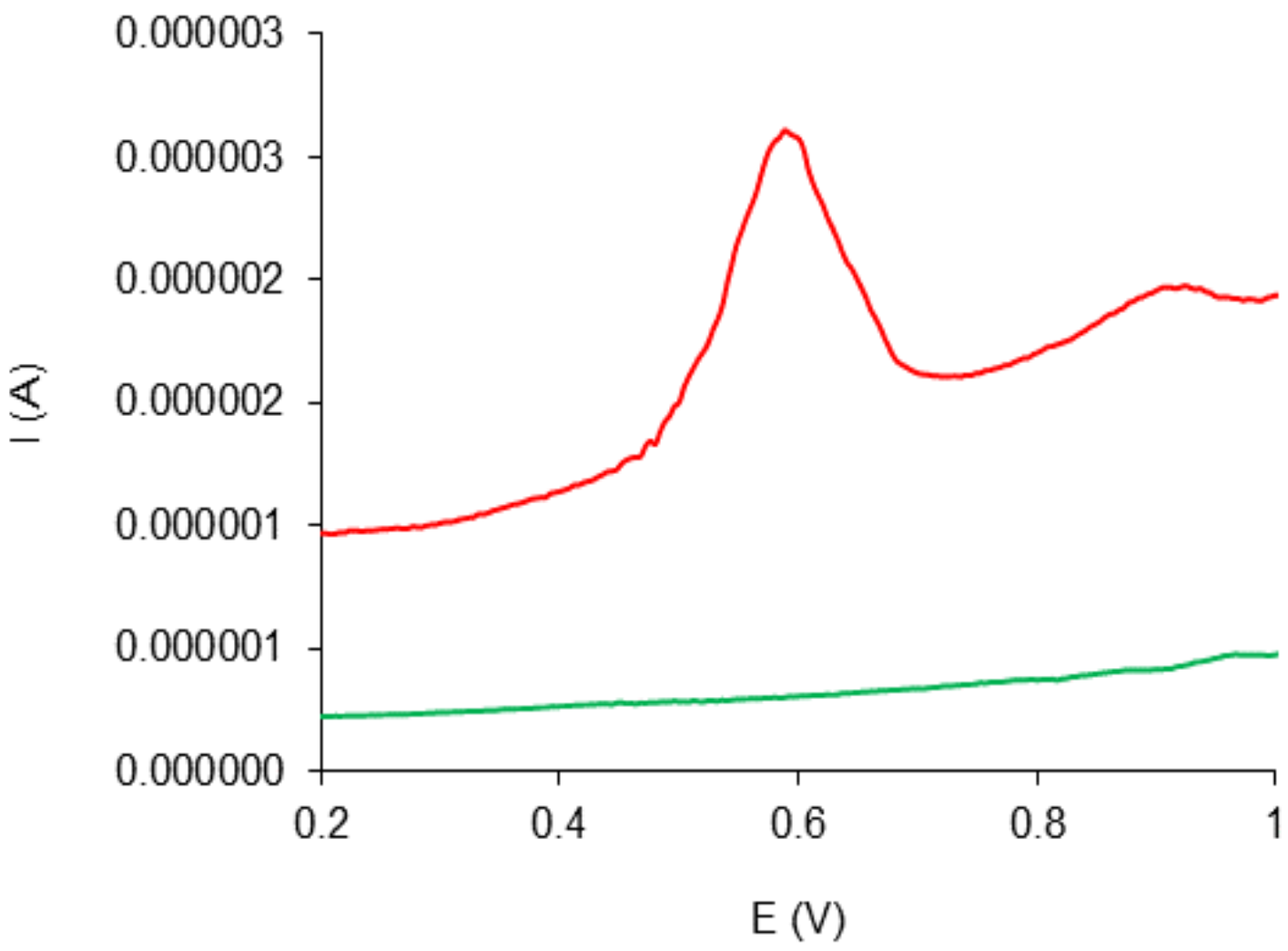

Figure 21. Conversion and selectivity values obtained throughout five consecutive reuses of GO@**2c** in the hydrolysis of benzonitrile in water after 6 h.

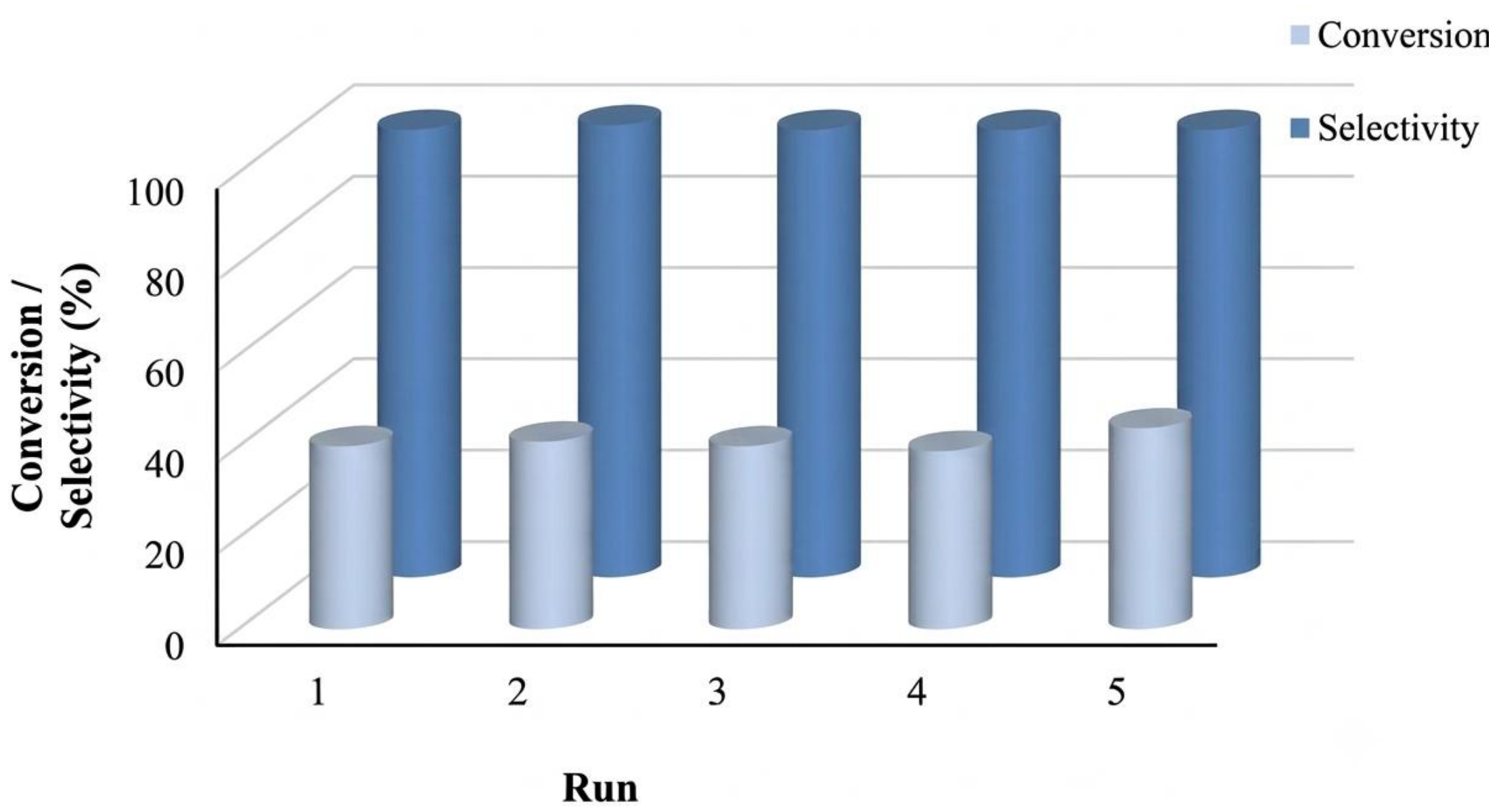

Figure S22. a) TEM and b) SEM images of GO@**2c** in the hydrolysis of benzonitrile after five reuses. The bars indicate a length of a) 200 nm; b) 10 µm).

a)

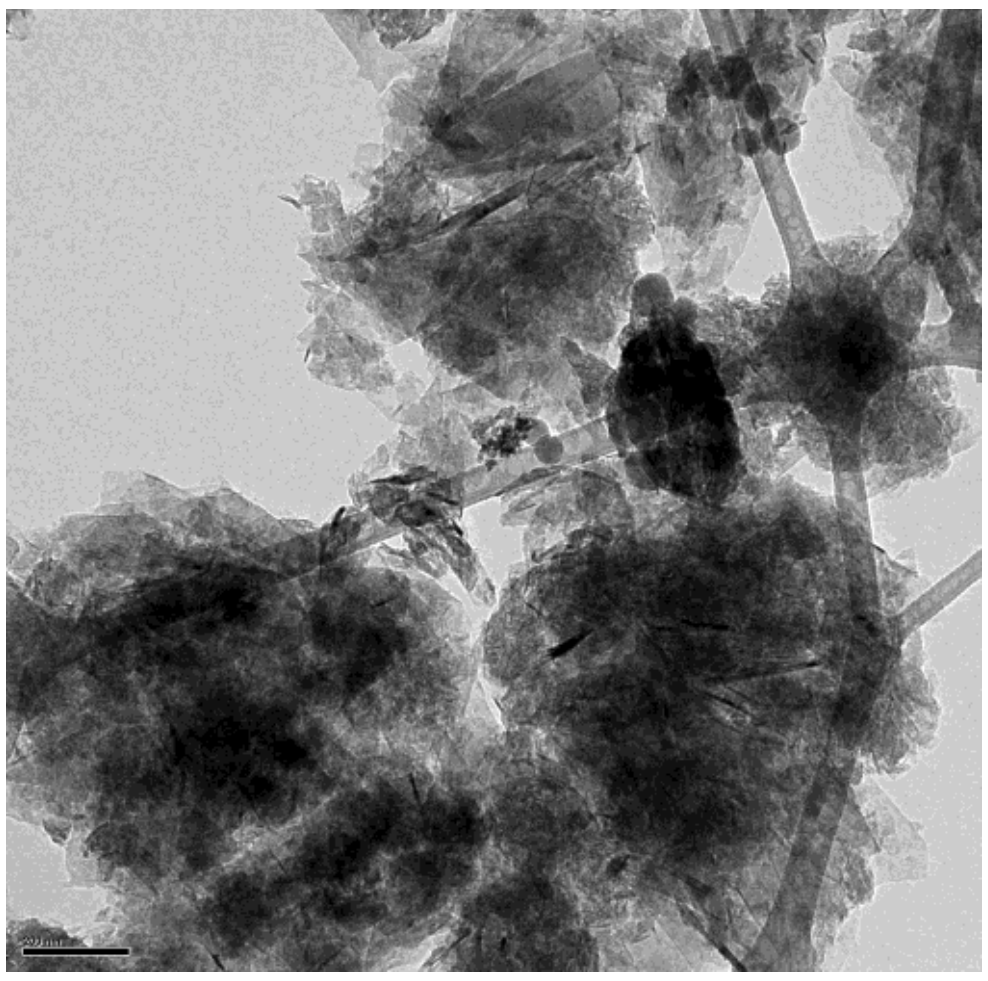

b)

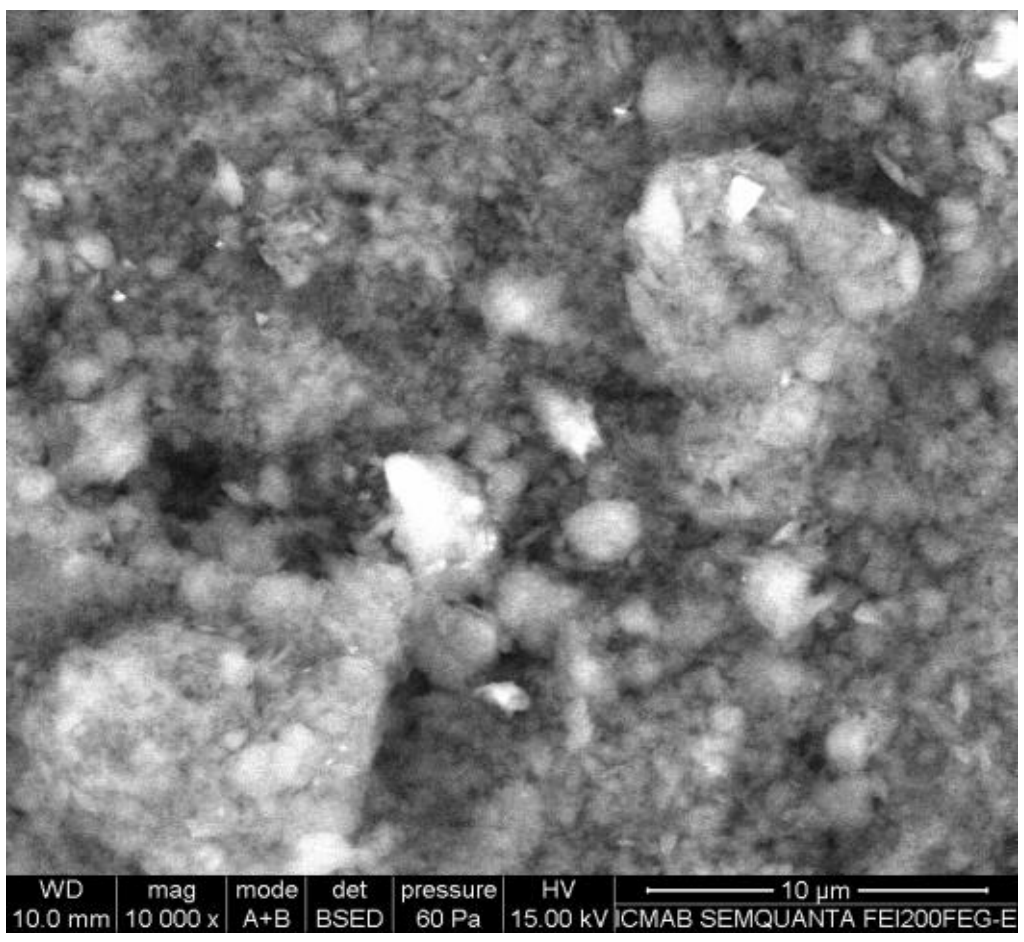

Figure S23. Representative NMR spectra in $(CD_3)_2CO$ of the obtained amides in the hydrolysis of nitriles in water, a) benzamide; b) methylbenzamide; c) chlorobenzamide; d) acrylamide; e) chloroacetonitrile.

a)

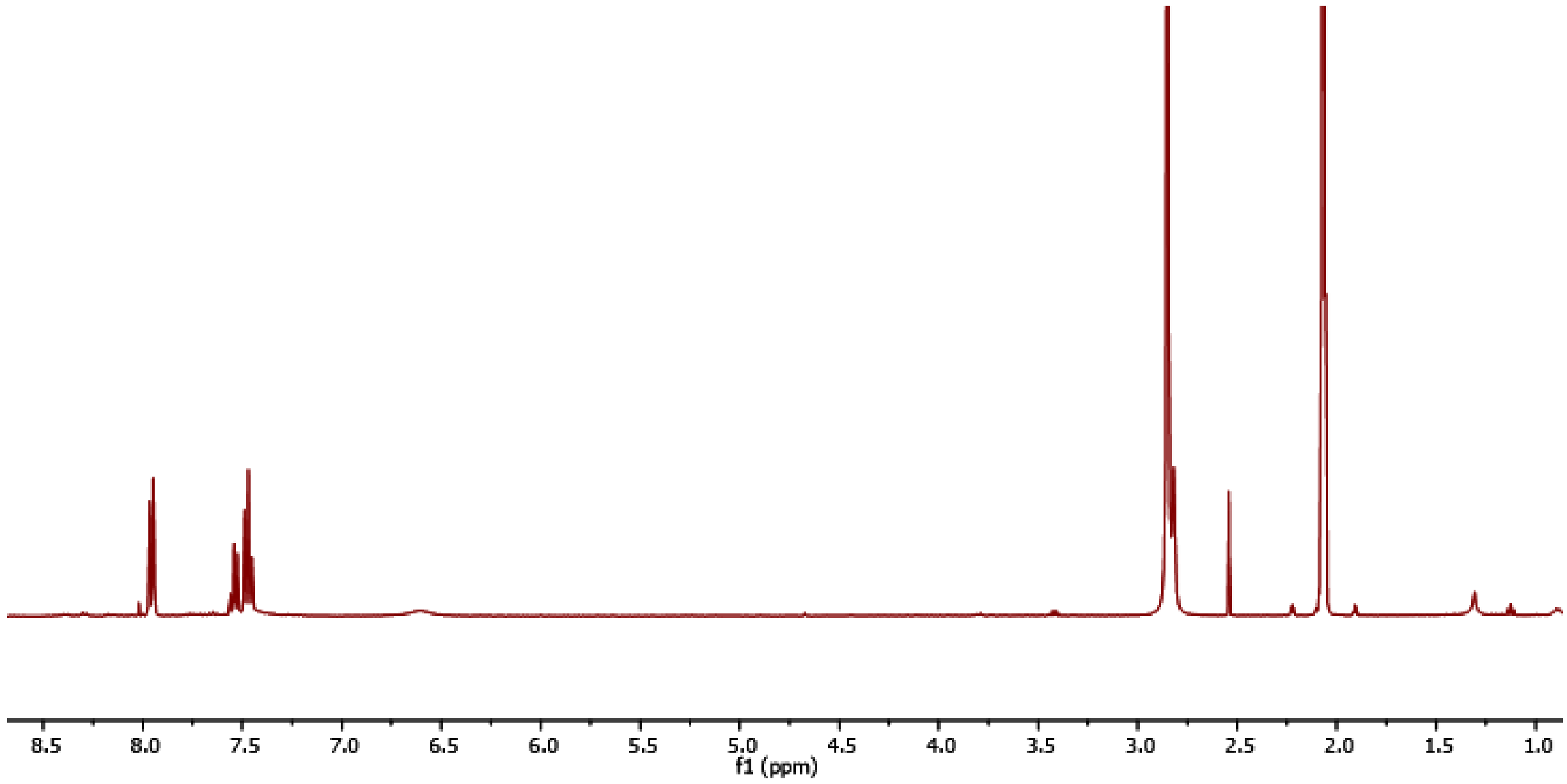

b)

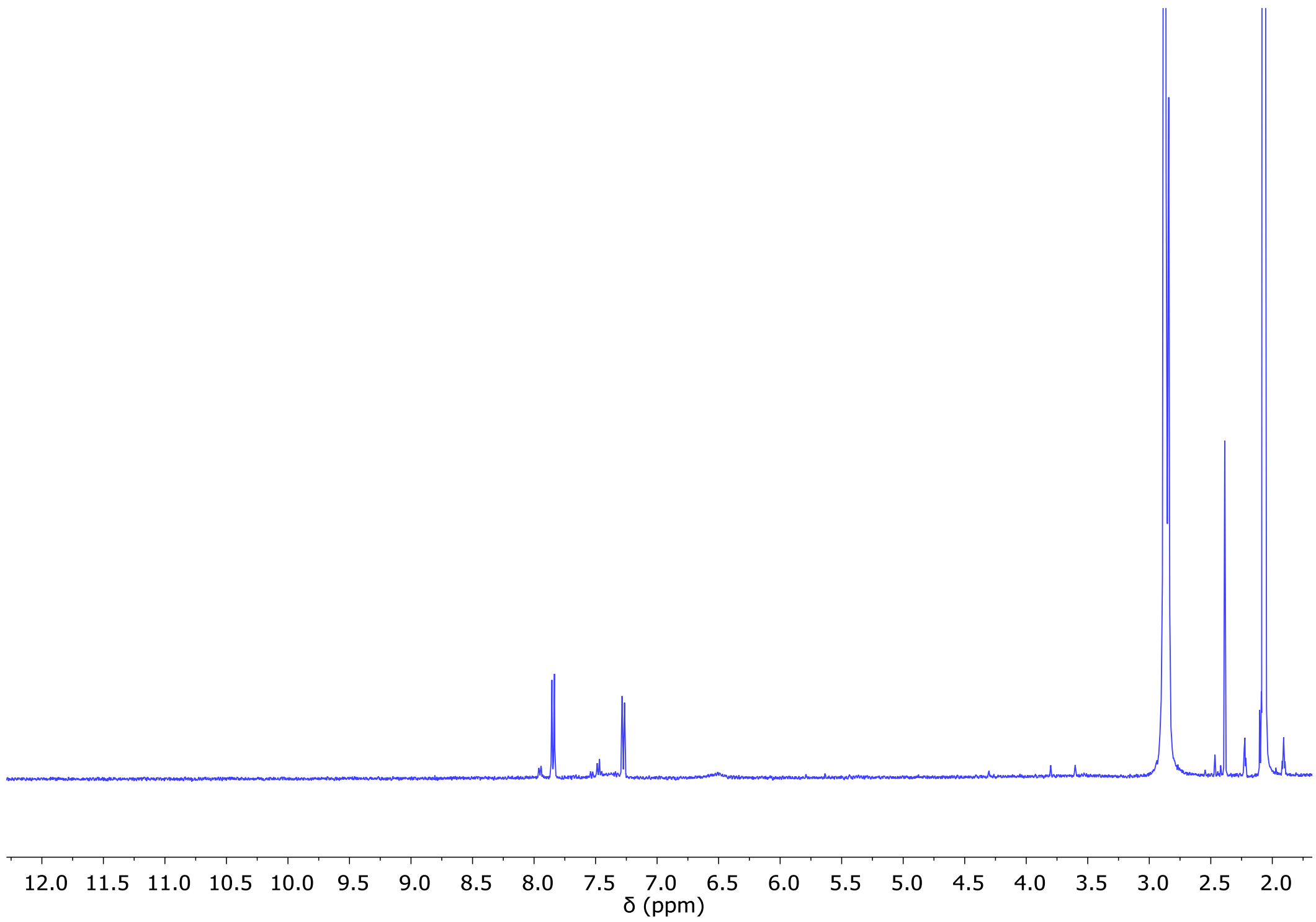

c)

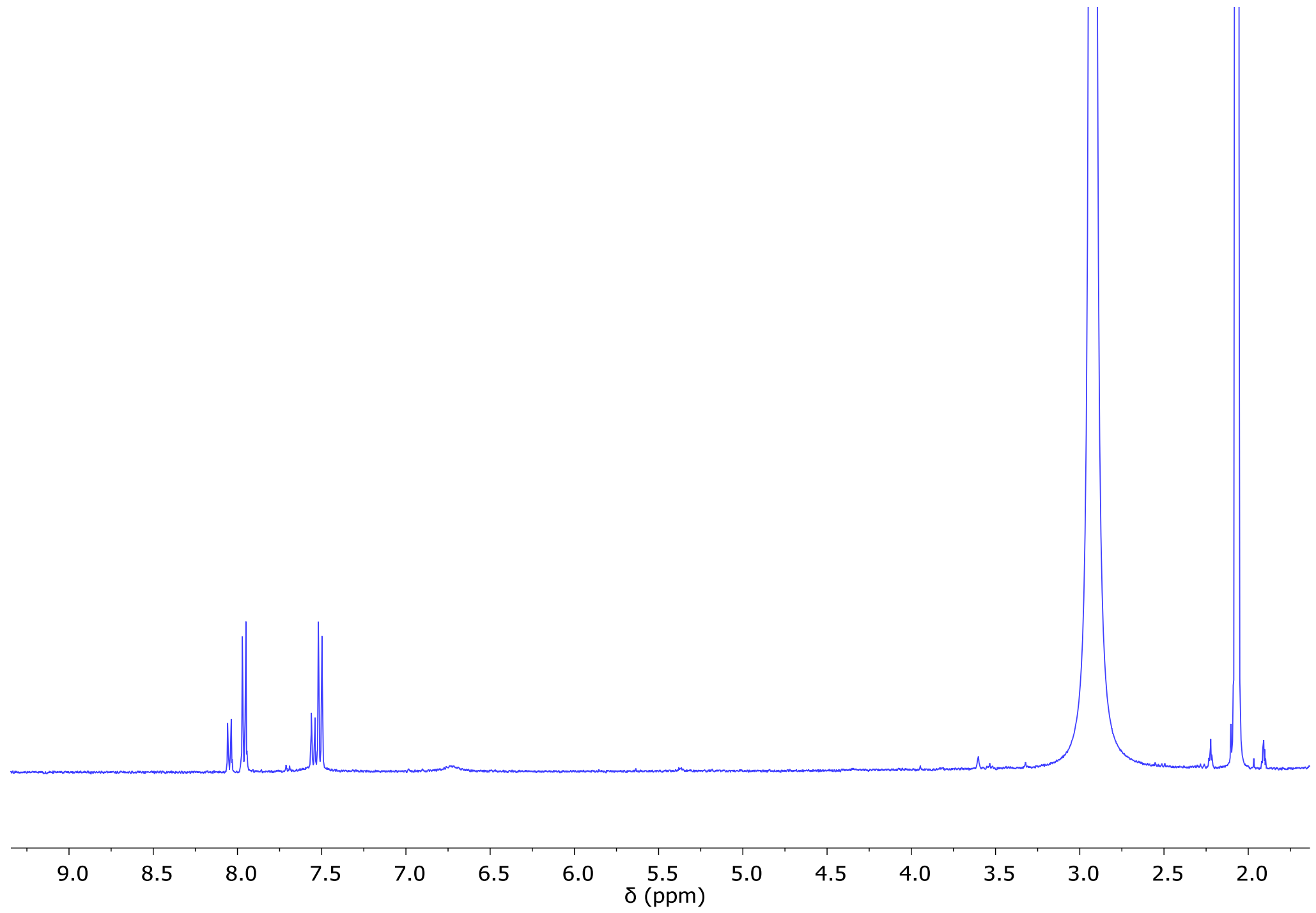
9.0 8.5 8.0 7.5 7.0 6.5 6.0 5.5 5.0 4.5 4.0 3.5 3.0 2.5 2.0
δ (ppm)

d)

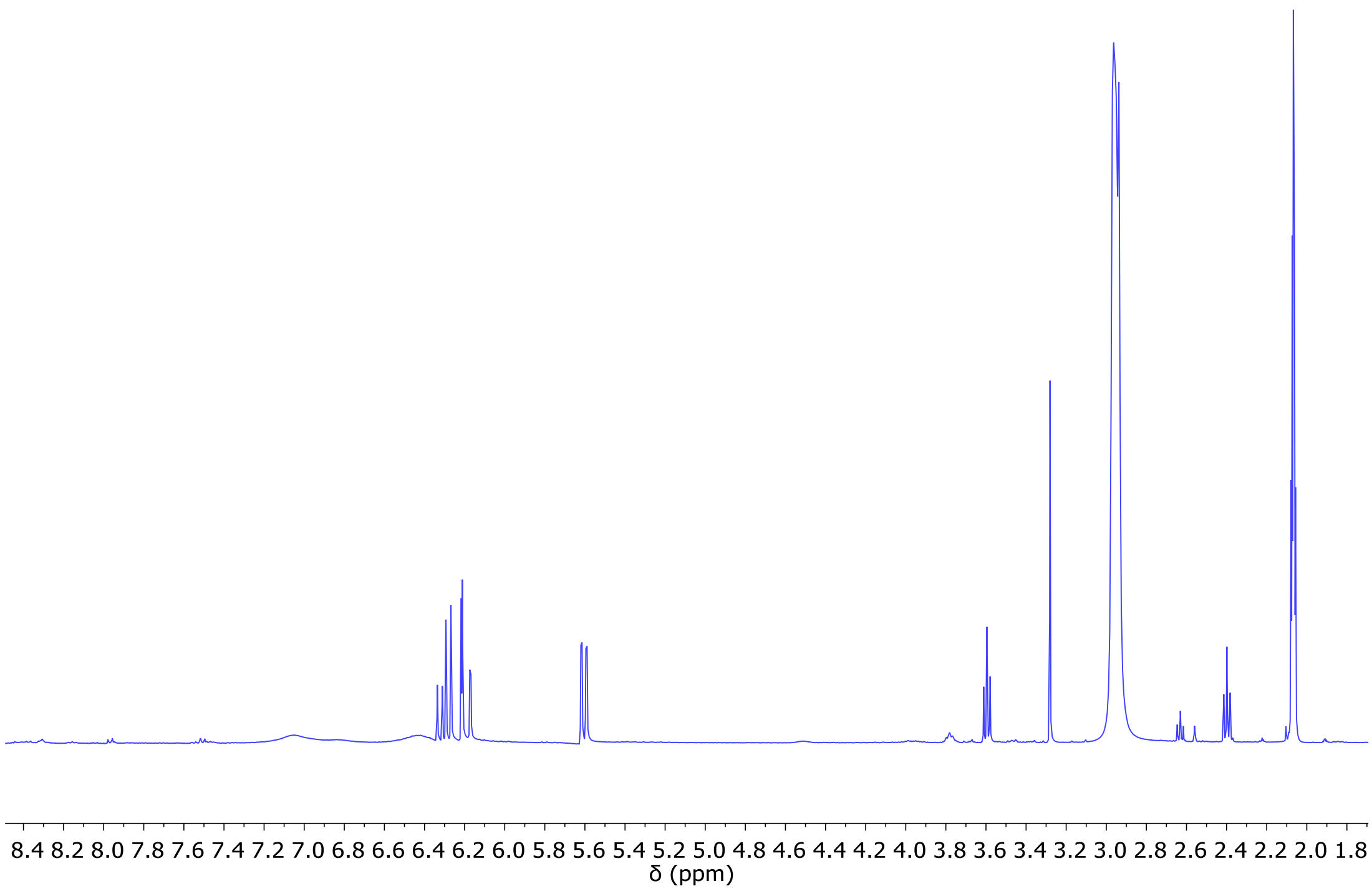
8.4 8.2 8.0 7.8 7.6 7.4 7.2 7.0 6.8 6.6 6.4 6.2 6.0 5.8 5.6 5.4 5.2 5.0 4.8 4.6 4.4 4.2 4.0 3.8 3.6 3.4 3.2 3.0 2.8 2.6 2.4 2.2 2.0 1.8
δ (ppm)

e)

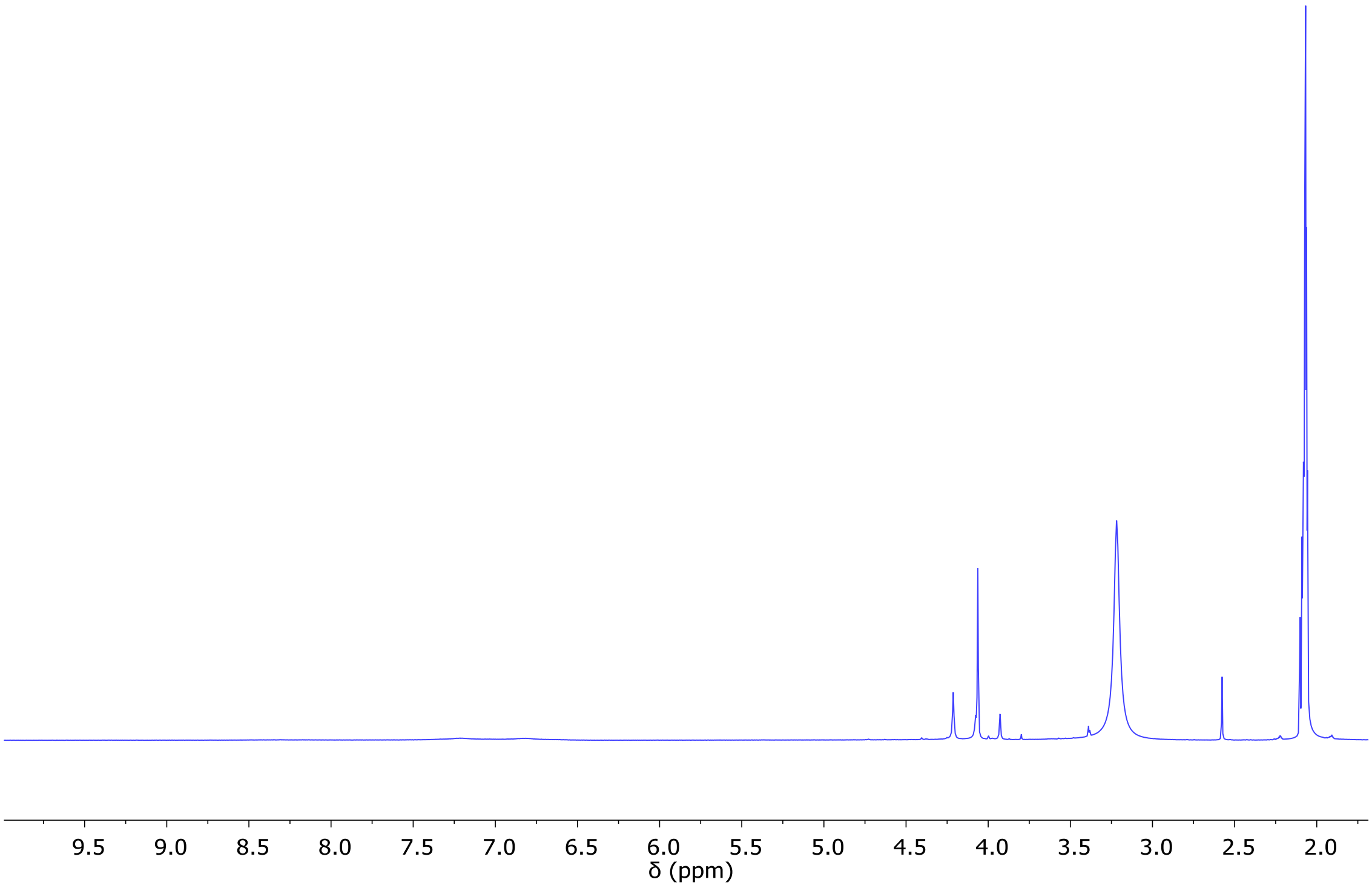

References

1. Mola, J.; Romero, I.; Rodríguez, M.; Bozoglian, F.; Poater, A.; Solà, M.; Parella, T.; Benet-Buchholz, J.; Fontrodona X.; Llobet, A.Mechanistic Insights into the Chemistry of Ru(II) Complexes Containing Cl and DMSO Ligands, *Inorg. Chem.*, **2007**, *46*, 10707–10716.
2. Romero, I.; Rodríguez, M.; Llobet, A.; Collomb-Dunand-Sauthier, M.-N.; Deronzier, A.; Parella T.; Stoeckli-Evans, H. Synthesis, structure and redox properties of a new ruthenium(II) complex containing the flexible tridentate ligand N,N-bis(2-pyridylmethyl)ethylamine, cis-fac-Ru(bpea)22+, and its homologue attached covalently to a polypyrrole film, *J. Chem. Soc., Dalton Trans.*, **2000**, 1689–1694.
